# Allele-specific regulatory effects on the pig transcriptome

**DOI:** 10.1093/gigascience/giad076

**Published:** 2023-09-30

**Authors:** Yu Lin, Jing Li, Li Chen, Jingyi Bai, Jiaman Zhang, Yujie Wang, Pengliang Liu, Keren Long, Liangpeng Ge, Long Jin, Yiren Gu, Mingzhou Li

**Affiliations:** Livestock and Poultry Multi-omics Key Laboratory of Ministry of Agriculture and Rural Affairs, College of Animal Science and Technology, Sichuan Agricultural University, Chengdu 611130, China; Livestock and Poultry Multi-omics Key Laboratory of Ministry of Agriculture and Rural Affairs, College of Animal Science and Technology, Sichuan Agricultural University, Chengdu 611130, China; Animal Breeding and Genetics Key Laboratory of Sichuan Province, Institute of Animal Genetics and Breeding, Sichuan Agricultural University, Chengdu 611130, China; Pig Industry Sciences Key Laboratory of Ministry of Agriculture and Rural Affairs, Chongqing Academy of Animal Sciences, Chongqing 402460, China; National Center of Technology Innovation for Pigs, Chongqing 402460, China; Livestock and Poultry Multi-omics Key Laboratory of Ministry of Agriculture and Rural Affairs, College of Animal Science and Technology, Sichuan Agricultural University, Chengdu 611130, China; Livestock and Poultry Multi-omics Key Laboratory of Ministry of Agriculture and Rural Affairs, College of Animal Science and Technology, Sichuan Agricultural University, Chengdu 611130, China; Livestock and Poultry Multi-omics Key Laboratory of Ministry of Agriculture and Rural Affairs, College of Animal Science and Technology, Sichuan Agricultural University, Chengdu 611130, China; Livestock and Poultry Multi-omics Key Laboratory of Ministry of Agriculture and Rural Affairs, College of Animal Science and Technology, Sichuan Agricultural University, Chengdu 611130, China; Animal Breeding and Genetics Key Laboratory of Sichuan Province, Institute of Animal Genetics and Breeding, Sichuan Agricultural University, Chengdu 611130, China; Pig Industry Sciences Key Laboratory of Ministry of Agriculture and Rural Affairs, Chongqing Academy of Animal Sciences, Chongqing 402460, China; National Center of Technology Innovation for Pigs, Chongqing 402460, China; Animal Breeding and Genetics Key Laboratory of Sichuan Province, Institute of Animal Genetics and Breeding, Sichuan Agricultural University, Chengdu 611130, China; College of Animal and Veterinary Sciences, Southwest Minzu University, Chengdu 610041, China; Animal Breeding and Genetics Key Laboratory of Sichuan Province, Sichuan Animal Science Academy, Chengdu 610066, China; Livestock and Poultry Multi-omics Key Laboratory of Ministry of Agriculture and Rural Affairs, College of Animal Science and Technology, Sichuan Agricultural University, Chengdu 611130, China

**Keywords:** allele-specific expression, imprinting, *cis-* and *trans-*regulatory effects, pig breeding

## Abstract

**Background:**

Allele-specific expression (ASE) refers to the preferential expression of one allele over the other and contributes to adaptive phenotypic plasticity. Here, we used a reciprocal cross-model between phenotypically divergent European Berkshire and Asian Tibetan pigs to characterize 2 ASE classes: imprinting (i.e., the unequal expression between parental alleles) and sequence dependent (i.e., unequal expression between breed-specific alleles). We examined 3 transcript types, including protein-coding genes (PCGs), long noncoding RNAs, and transcripts of unknown coding potential, across 7 representative somatic tissues from hybrid pigs generated by reciprocal crosses.

**Results:**

We identified a total of 92 putative imprinted transcripts, 69 (75.00%) of which are described here for the first time. By combining the transcriptome from purebred Berkshire and Tibetan pigs, we found ∼6.59% of PCGs are differentially expressed between breeds that are regulated by *trans-*elements (e.g., transcriptional factors), while only ∼1.35% are attributable to *cis* (e.g., promoters). The higher prevalence of *trans-*PCGs indicates the dominated effects of *trans*-regulation in driving expression differences and shaping adaptive phenotypic plasticity between breeds, which were supported by functional enrichment analysis. We also found strong evidence that expression changes mediated by *cis-*effects were associated with accumulated variants in promoters.

**Conclusions:**

Our study provides a comprehensive map of expression regulation that constitutes a valuable resource for the agricultural improvement of pig breeds.

## Background

Mammalian genomes are diploid and consist of 2 parental copies for each gene locus [1]. Allele-specific expression (ASE) occurs in diploid genomes when one allele is preferentially expressed over the other [2, 3]. Genetic and epigenetic differences between alleles are frequently associated with ASEs occurring at different developmental stages and leading to functional consequences for a variety of biological processes [2, 3].

ASEs can be classified into 2 distinct classes: imprinting and sequence dependent [2]. The former refers to the broader class of epigenetic phenomena that depend on the preferential expression of either paternal or maternal alleles and is closely associated with epigenetic markers, such as DNA methylation and histone modifications [2, 4]. To date, imprinted genes constitute approximately 1% of the human and mouse genomes [5]. In contrast, sequence-dependent ASEs represent the differential expression between alleles based on nucleotide similarity [2]. In most cases, they manifest as the expression difference between breeds or strains within a species and are most likely driven through the action of *cis-*regulatory elements (typically, promoters and enhancers), which are functional noncoding DNA sequences that modulate the expression of nearby genes [6, 7]. In diploid individuals, genetic variants at *cis-*regulatory elements may induce differential expression between breeds or strains and can be inherited by F1 progenies [7, 8]. However, expression difference can also occur due to *trans-*acting factors, which are diffusible components (e.g., transcription factors) that affect the expression of distal genes by interacting with their target sequences [6, 7]. *Trans-*regulatory effects have an equal opportunity to influence both alleles and thus eliminate differential expression in F1 progenies [8].

Both *cis-* and *trans-*regulatory effects are responsible for adaptive phenotypic plasticity between breeds or strains. Recent studies used RNA sequencing (RNA-seq) data and an allele-specific expression strategy to evaluate *cis-* and *trans-*regulatory effects in a wide range of species, including yeast [9], fruit fly [10, 11], chicken [12], and mouse [13, 14]. These studies identified a large number of single-nucleotide variants (SNVs) in hybrid F1 progenies generated from reciprocal crosses between 2 genetically and phenotypically divergent breeds or strains. These heterozygous SNVs were then used to distinguish allele-specific RNA-seq reads and evaluate the expression difference between breeds or strains. By measuring the expression levels of the 2 parental breeds or strains, the authors identified different mechanisms of expression regulation, revealing the extensive adaptive evolutionary differences between them.

In this study, we conducted a comprehensive investigation of allele-specific regulatory effects on the pig transcriptome using a reciprocal cross-model between European Berkshire and Asian Tibetan pigs. Our analysis identified 92 putative imprinted transcripts and revealed a substantial number of both *cis-* and *trans-*regulated effects. These findings shed light on the underlying regulatory mechanisms driving expression changes between different pig breeds and offer valuable insights for genetic breeding strategies.

## Materials and Methods

### Sample collection

**Figure 1:**
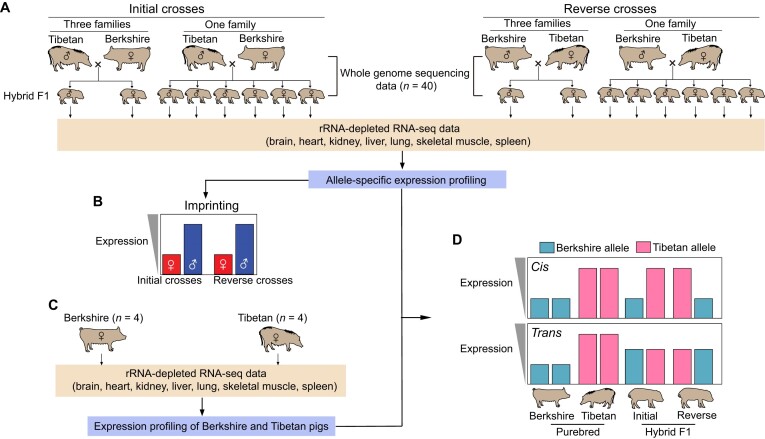
Study design. (A) Schematic diagram of reciprocal crosses between genetically distinct Berkshire and Tibetan pig breeds. We collected a total of 163 rRNA-depleted RNA-seq libraries across 7 tissues (brain, heart, kidney, liver, lung, skeletal muscle, and spleen) from 24 F1 progenies, including 145 libraries newly generated and 18 libraries downloaded. Whole-genome sequencing data (*n* = 40) were collected, including 22 newly generated and 18 downloaded. (B) Schematic representation of imprinting. (C) A total of 54 rRNA-depleted RNA-seq libraries across 7 tissues from purebred Berkshire (*n* = 4) and Tibetan pigs (*n* = 4) were generated. (D) The schematic diagram illustrates example expression patterns for different regulatory categories between breeds. *Cis*: concordant significant expression divergence between breeds was observed in the F0 and F1 groups. *Trans*: significant expression divergence between breeds was observed in the F0 but not the F1 group. See detailed information in Materials and Methods.

Reciprocal crosses were performed between Berkshire and Tibetan pigs, generating a total of 8 families (4 initial crosses and 4 reverse crosses; Fig. 1A and Supplementary Fig. S1A). We downloaded whole-genome sequencing data for 18 individuals (6 parent–child trios; 3 initial and 3 reverse crosses [15]), along with ribosomal RNA (rRNA)–depleted RNA-seq data for 3 tissues of the 6 newly born F1 females (including brain, liver, and skeletal muscle, *n* = 18 libraries; Supplementary Fig. S1A and Supplementary Data S1). We collected another 4 tissues (heart, kidney, lung, and spleen) from the above F1 progenies to produce rRNA-depleted RNA-seq data. We produced 10 newly born F1 progenies from parents of the aforementioned 6 families and collected samples of blood and the other 7 tissues (brain, heart, kidney, liver, lung, skeletal muscle, and spleen) for each individual to generate whole-genome sequencing and rRNA-depleted RNA-seq data (Supplementary Fig. S1A and Supplementary Data S1). In addition, we independently performed 1 initial cross (2 parents with 2 F1 progenies) and 1 reverse cross (2 parents with 6 F1 progenies) and again collected samples of blood and each of the aforementioned 7 tissues from individuals of 8 F1 progenies (Supplementary Fig. S1A and Supplementary Data S1). To evaluate the expression levels of the parental breeds, we collected the aforementioned 7 tissues from purebred Berkshire (*n* = 4) and Tibetan (*n* = 4) females. Finally, the skeletal muscle from additional purebred Berkshire and Tibetan pigs was collected to perform single-nucleus RNA-seq.

### rRNA-depleted RNA-seq and data processing

We generated a total of 145 rRNA-depleted RNA-seq libraries across 7 tissues of F1 progenies, as well as 54 rRNA-depleted RNA-seq libraries across 7 tissues for purebred Berkshire and Tibetan pigs. Total RNA was extracted using the RNeasy Mini Kit (Qiagen). The rRNA depletion protocol (Ribo-Zero kit; Epicentre) and the Illumina TruSeq RNA-seq library protocol were then implemented to construct strand-specific RNA-seq libraries. The libraries were quantified using the Qubit dsDNA High Sensitivity Assay Kit (Invitrogen) and sequenced with 150-bp paired-end reads using the Illumina HiSeq X Ten platform (RRID:SCR_016385) or 100-bp paired-end reads using the BGISEQ-500 platform (RRID:SCR_017979). The annotations of protein-coding genes (PCGs; Ensembl website, version *Sus scrofa* 107) and 2 transcript types (long noncoding RNAs [lncRNAs] and transcripts of unknown coding potential [TUCPs] [16]) were integrated to build a complete transcript annotation in the pig genome. The Kallisto software (RRID:SCR_016582) (version 0.44.0) [17] was used to quantify the expression and count the read number of PCGs, lncRNAs, and TUCPs using the 217 RNA-seq libraries (including 18 downloaded and 199 newly generated data).

### Whole-genome sequencing and variants calling

The genomic DNA was exacted from blood samples (*n* = 22) using the TIANamp Genomic DNA Kit (TIANGEN, DP304). Sequencing libraries were constructed and sequenced on the Illumina HiSeq X Ten platform with 150-bp paired-end reads or on the BGISEQ-500 platform with 100-bp paired-end reads. Low-quality and adapter sequencing data were removed, and the filtered sequences were mapped to the reference pig genome (version *S. scrofa* 11.1) using the Burrows–Wheeler Aligner software (RRID:SCR_010910) (version 0.7.8) with default parameters [18]. Potential PCR duplicates were removed using the module “MarkDuplicates” of the Picard software (RRID:SCR_006525) (version 2.0.1). The module “HaplotypeCaller” in the Genome Analysis Toolkit (GATK; version 4.2.6) [19] was used to detect SNVs and insertions and deletions (indels) for each individual. For individuals within each family, the genetic variants were aggregated into a multisample VCF file using the module “GVCFGenotyper” of GATK. Low-quality SNVs and indels were discarded using the following conditions on GATK: “QD < 10.0 || FS > 60.0 || MQ < 40.0 || MQRankSum <–12.5 || ReadPosRankSum <–8.0 || GQ < 30.” Unmapped scaffolds, sex chromosomes, and the mitochondrial genome were also removed from further analyses. In addition, SNVs and indels with very high or low coverage (defined as the top and bottom ∼1% of the distribution, respectively, or a depth-based *z* score <–2.58 and >2.58) were also discarded. The final SNV dataset was used to perform principal component analysis using GCTA (version 1.93.2) [20] and infer genetic structure using the Structure software (RRID:SCR_002151) (version 2.3.4) [21].

### Assignment of allele-specific RNA-seq reads

The heterozygous SNVs of each F1 progeny were phased using a trio-based strategy, when at least one of the parents was homozygous [22]. To mitigate the potential bias introduced by mapping, we employed a strategy to address SNVs in the F1 progeny. Specifically, we substituted the nucleotide (A/T/G/C) of each heterozygous SNV with “N” to create an N-masked genome [23, 24]. This N-masked genome was then utilized for subsequent analysis of allele-specific expression, reducing the impact of mapping biases. The high-quality RNA-seq reads of hybrid F1 progenies (*n* = 163; including 18 downloaded and 145 newly generated) were aligned to the “N”-masked reference genome using STAR (RRID:SCR_004463) (version 2.6.0c) [25] with default parameters. The software SNPsplit (version 0.3.4) [26] was used to distinguish the allele-specific RNA-seq reads using the phased heterozygous SNVs. The read counts of allele-specific RNA-seq reads were quantified using the software Kallisto (version 0.44.0) [17].

### Identification of imprinted transcripts

For each tissue, the allele-specific RNA-seq reads of each PCG, lncRNA, and TUCP were classified into 2 groups according to their respective parental origins (i.e., maternal or paternal allele). The R package DEseq2 (RRID:SCR_015687) [27] was employed to calculate the *P* values and estimate significant differences in expression between parental alleles using the allele-specific RNA-seq read counts. The *P* values were further corrected for multiple testing using the Benjamini–Hochberg procedure, and the putative imprinted PCGs, lncRNAs, and TUCPs were obtained under a strict corrected *P* value cutoff of 0.05.

### Classification of expression regulatory categories between breeds

We investigated the regulatory categories associated with expression differences between breeds for each tissue according to a previous study [12]. Specifically, we used the R package DEseq2 [27] to identify significant differential expression for each PCG, lncRNA, and TUCP between breeds in both the F0 (**P**) and F1 (**F**) groups under a corrected *P* value of 0.05. Fisher's exact test was used to evaluate breed-specific expression ratio differences between the **P** and **F** groups under a corrected *P* value of 0.05 to detect *trans-*regulatory effects (**T**). The nonimprinted PCGs, lncRNAs, and TUCPs with detectable expression were classified into 7 categories according to the following criteria:

1. *Cis*: significant differences detected in **P** and **F**, but not in **T**.
2. *Trans*: significant differences detected in **P** and **T**, but not in **F**.
3. *Cis + trans*: significant differences detected in **P, F**, and **T**; the expression ratios between breeds were concordant between **P** and **F**.
4. *Cis × trans*: significant differences detected in **P, F**, and **T**; the expression ratios between breeds were opposite between **P** and **F**.
5. Compensatory: significant differences detected in **F** and **T**, but not in **P**.
6. Conserved: no significant differences detected in **P, F**, and **T**.
7. Ambiguous: All other patterns.

See detailed information of biological significance for each regulatory category in Supplementary Fig. S2.

### Single-nucleus RNA-seq and data processing

The nuclei of skeletal muscle obtained from purebred Berkshire (*n* = 2) and Tibetan pigs (*n* = 2) were purified and cut into pieces and then digested in the GEXSCOPE Tissue Dissociation Solution (Singleron Biotechnologies) at 37°C for 15 minutes. After digestion, a 40-micron sterile strainer was used to isolate the nucleus and remove other material. The resulting nuclei were centrifuged, and nucleus-containing pellets were resuspended in phosphate-buffered saline (PBS) (HyClone). The mixture was centrifuged and again resuspended in PBS. Single-nucleus RNA-seq libraries were constructed following the Singleron GEXSCOPE protocol (Singleron Biotechnologies). The individual libraries were diluted to 4 ng/mL and pooled for sequencing on a HiSeq X Ten platform (Illumina) with 150-bp paired-end reads. Low-quality reads and adaptor sequences were removed before generating gene expression matrices using the software CeleScope (RRID:SCR_023553). The Seurat package (RRID:SCR_007322) (version 4.0.4) [28] available in R (version 4.0.5) was employed to filter cells. The independent samples were merged into a single object and clustered without supervision using the harmony package (RRID:SCR_023543) (version 0.1.0) [29]. Uniform Manifold Approximation and Projection (UMAP, RRID:SCR_018217) [30] was applied to project all cells onto a 2-dimensional map. The cell types were annotated based on the expression of canonical markers. The cell type–specific highly expressed PCGs were identified using the “FindAllMarkers” function available in the Seurat package with the following parameters: log_2_ fold-change > 0.25 and corrected *P* value <0.05.

### Measurement of sequence conservation

PhastCons and PhyloP scores calculated by multiple alignment of 46 mammalian genomes were converted to the reference pig genome using the UCSC LiftOver tool (RRID:SCR_018160). Average PhastCons and PhyloP scores were calculated for exonic regions of PCGs to compare sequence conservation across different regulatory categories. To calculate nonsynonymous/synonymous mutations (dN/dS) ratios between pig versus human and pig versus mouse, we downloaded single-copy ortholog information from the Ensembl website (version 107). The protein sequences of these orthologs were aligned between pig versus human and pig versus mouse using the software MUSCLE (RRID:SCR_011812) (version 3.8.31) [31]. The aligned files of protein sequences were then converted into nucleotide sequences, which were then used to calculate dN/dS ratios for each ortholog pair using the software KaKs_Calculator (RRID:SCR_022068) (version 3.0) [32].

### Functional enrichment analysis

Functional enrichment analysis was performed using the Metascape tool (RRID:SCR_016620) (version 3.5.20211218) [33]. Briefly, the PCGs in the pig genome were converted to human symbols according to pig–human orthologs and then used as input data for Metascape. For the enrichment analysis in each tissue, we utilized PCGs that demonstrated expression evidence (average transcripts per million [TPM] >0.5). The respective numbers of PCGs meeting this criterion were as follows: brain (*n* = 13,573), heart (*n* = 12,360), kidney (*n* = 13,434), liver (*n* = 12,141), lung (*n* = 13,494), skeletal muscle (*n* = 11,995), and spleen (*n* = 12,624). These PCGs constituted the background for the enrichment analysis in their respective tissues.

## Results

### Allele-specific expression profiling across pig tissues

To investigate allele-specific expression profiling in pigs, we conducted an analysis using a comprehensive set of 163 RNA-seq libraries. These libraries were derived from 7 somatic tissues obtained from a total of 24 F1 progenies, which were generated through reciprocal crosses between 2 pig breeds that were genetically and phenotypically distinct (Fig. 1A, Supplementary Fig. S1 and Supplementary Data S1; see details in Materials and Methods). To build a complete transcriptome for further analysis, we combined the annotations of PCGs and 2 transcript types (lncRNAs and TUCPs) from a previous study [16]. Considering random X-inactivation of expression in mammalian genomes [3], we analyzed 19,990 PCGs, 17,770 lncRNAs, and 2,205 TUCPs that encoded in autosomes.

The phasing of allele-specific RNA-seq reads in F1 progenies depends on the genotyping information of heterozygous SNVs [12, 14]. For this purpose, we obtained whole-genome sequencing data for 22 individuals and downloaded the publicly available sequencing data for another 18 individuals (Berkshire pigs [*n* = 8], Tibetan pigs [*n* = 8], and F1 progenies [*n* = 24]) with an average of 126.25× coverage (315.63 Gb) per individual (Fig. 1A, Supplementary Figs. S1A and S3A; see details in Materials and Methods). We identified ∼11.02 million (M) heterozygous SNVs for each F1 progeny, of which ∼95.35% were successfully phased using a trio-based strategy [22] (Supplementary Figs. S3B–D and S4A, B). This relatively high density of phased heterozygous SNVs (∼4.64 SNVs per kb) allowed us to assign the expression of ∼11,880 PCGs (72.61% of expressed [TPM >0.5]), ∼2,982 lncRNAs (28.34% of expressed [TPM >0.1]), and ∼513 TUCPs (36.23% of expressed [TPM >0.1]) to their parental alleles (see Materials and Methods; Supplementary Fig. S4C).

Taken together, we successfully constructed allele-specific expression profiles for 3 transcript types across 7 tissues based on a reciprocal cross-model between Berkshire and Tibetan pigs, considering the high genetic divergence between families, the similar genetic background within families, and differences between sexes (Supplementary Fig. S2E). This strategy allowed us to systematically explore regulatory differences responsible for expression changes between parental and breed-specific alleles.

### The landscape of imprinted transcripts in the pig genome

**Figure 2:**
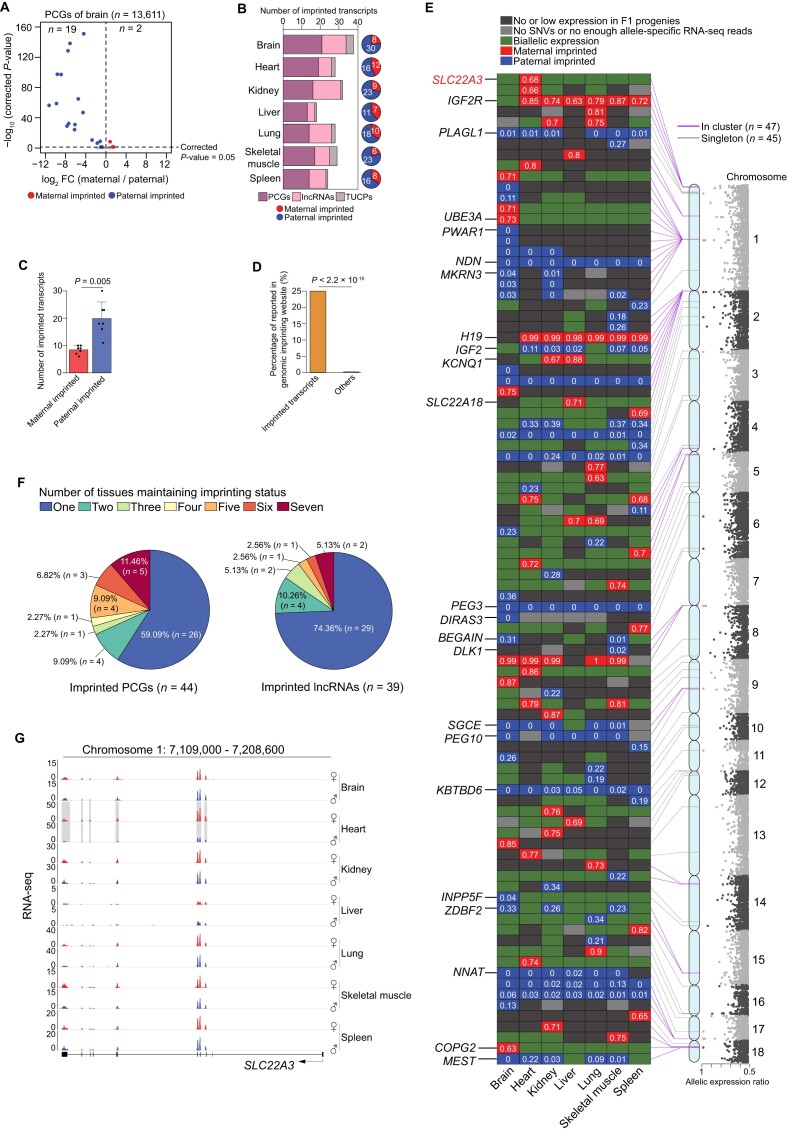
The landscape of imprinted transcripts across tissues. (A) Identification of imprinted PCGs in the brain. See the results of others tissues in Supplementary Figs. S5–S7. (B) The number of putative imprinted PCGs, lncRNAs, and TUCPs (bars, left) for each tissue. The pie chart on the right shows the number and proportion of maternal and paternal imprinted transcripts. (C) The bar plots show the significantly higher number of paternal imprinted transcripts (blue, right) compared to maternal (red, left). *P* value was calculated using a paired Student's *t* test. (D) The comparison of the proportion reported in the genomic imprinting website for putative imprinted transcripts (left) and others (right). The *P* value was calculated using a χ^2^ test. (E) The heatmap shows the allele-specific expression patterns for the union set of putative imprinted PCGs (*n* = 44), lncRNAs (*n* = 39), and TUCPs (*n* = 9) that were detected in at least 1 tissue. The cluster (i.e., 2 adjacent imprinted transcripts were located with 1 Mb genomic distance, purple lines) and chromosome information are shown on the right. Manhattan plot showing the allelic expression ratio (i.e., the ratio of preferential expressed allele, the maximal values across 7 tissues were used to display). The symbols of putative imprinted transcripts that were reported in the genomic imprinting website are shown on the left. The allele-specific expression ratio is shown for each putative imprinted transcript across 7 tissues (0 means 100% expression from the paternal allele while 1 means 100% expression from the maternal allele). (F) The pie charts show the number of tissues that maintained the imprinting status in PCGs (left) and lncRNAs (right). TUCPs were ignored due to smaller number. (G) Expression levels for maternal and paternal alleles across 7 tissues for the heart-specific maternally imprinted PCG *SLC22A3*. The expression disparity between maternal and paternal alleles in the heart is highlighted in gray.

Imprinting is a form of epigenetic regulation that causes the preferential expression of either maternally or paternally inherited alleles [34] (Fig. 1B). To construct a comprehensive category of imprinted transcripts in the pig genome, we examined significant expression differences between parental alleles for each of 7 tissues under a strictly corrected *P* value of 0.05 (see Materials and Methods). We identified 13 to 22 (∼16.43) putative imprinted PCGs, 4 to 15 (∼9.71) lncRNAs, and 1 to 4 (∼2.14) TUCPs in each tissue, with a significantly higher number of paternal versus maternal alleles when combining the 3 transcript types (∼19.57 vs. ∼8.57, *P* = 0.005, paired Student's *t* test; Fig. 2A–C and Supplementary Figs. S5–S7). This observation is in accordance with previous observations in human and mouse genomes [3, 5].

Among the union set of putative imprinted PCGs (*n* = 44), lncRNAs (*n* = 39), and TUCPs (*n* = 9) that were detected in at least 1 tissue, 69 (75.00%) were here first described in vertebrates, while the remaining 23 (25.00%) were reported accessed online (Fig. 2D and Supplementary Data S2). Moreover, more than 50% (47 of 92) were located into 13 clusters (i.e., 2 imprinted transcripts located within 1 Mb genomic distance; Fig. 2E and Supplementary Data S2), which is also typical of known imprinted genes [3] and further demonstrate the reliability of imprinting identification.

Consistent with previous findings in hybrid mouse [3], we observed a tissue-dependent manner of imprinting, with 59.09% of PCGs and 74.36% of lncRNAs identified in a single tissue (Fig. 2F), suggesting imprinting impacts tissue-specific function. As an example, *SLC22A3* (solute carrier family 22 member 3) displayed exclusive maternal imprinting specifically in the heart tissue (Fig. 2E and G). Mutations in this gene have been linked to heart pathogenesis [35].

### Extensive *trans-*regulatory divergence between two pig breeds

**Figure 3:**
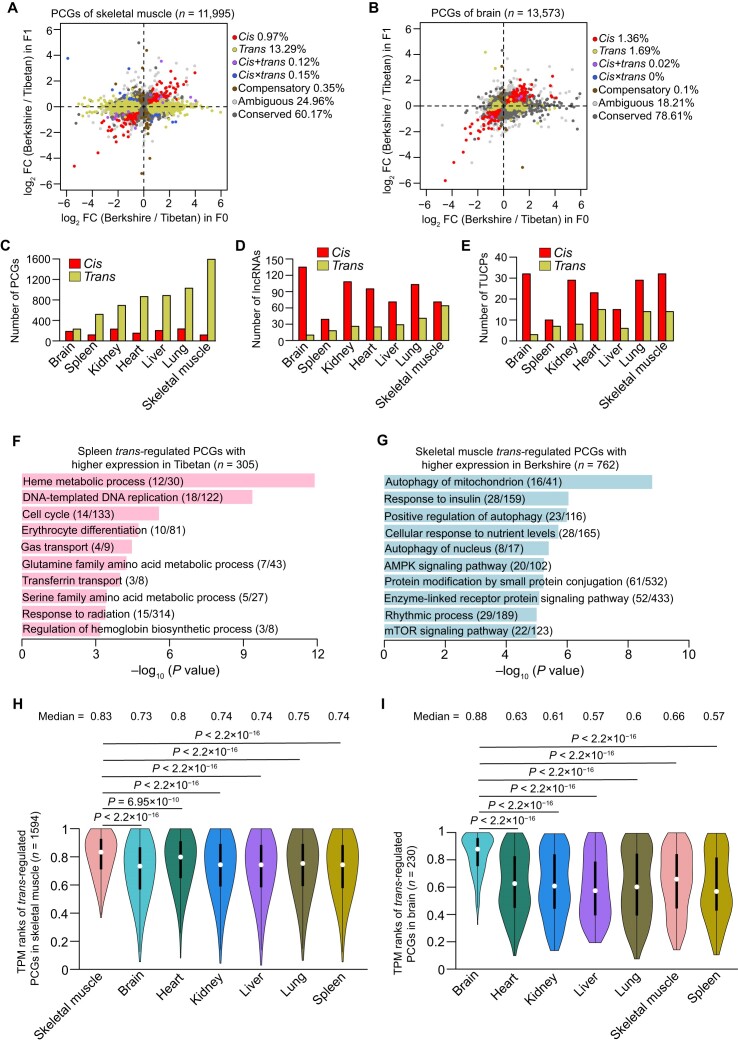
Classification of expression regulatory categories between pig breeds. (A, B) Classification of *cis-* and *trans-*regulatory effects of PCGs in skeletal muscle (A) and brain (B). See the results obtained in other tissues in Supplementary Figs. S9–S11. (C–E) The number of *cis-* (bars, red) and *trans-*regulatory (bars, yellow) effects in PCGs (C), lncRNAs (D), and TUCPs (E). (F, G) The enriched GO and KEGG pathways for *trans-*regulatory PCGs with higher expression in the spleen of Tibetan pigs (F) and the skeletal muscle of Berkshire pigs (G). (H, I) The TPM ranks of each tissue for *trans-*regulatory PCGs in the skeletal muscle (H) and the brain (I). The ranks represent the normalized expression levels in each tissue, from lower to higher. The *P* values were calculated using the Wilcoxon rank-sum test.

The observed phenotypic differences can be explained by both *cis-* and *trans-*regulatory effects changing gene expression between breeds. To identify *cis-* and *trans-*regulatory effects, we performed rRNA-depleted RNA-seq for purebred Berkshire (*n* = 4) and Tibetan pigs (*n* = 4) across the aforementioned 7 tissues (*n* = 54), generating ∼15.84 Gb of high-quality data per library (Fig. 1C and Supplementary Fig. S8). For each tissue, we examined the expression differences between breeds in both F0 (i.e., the expression of purebred Berkshire and Tibetan pigs) and F1 groups (i.e., the expression of Berkshire and Tibetan alleles in F1 progenies from reciprocal crosses) using 11,995 to 13,573 (∼12,803) nonimprinted PCGs, 2,050 to 5,255 (∼3,535) nonimprinted lncRNAs, and 383 to 809 (∼577) nonimprinted TUCPs. These transcripts were expressed in both groups and included phased SNVs in F1 progenies to make it possible to distinguish breed-specific alleles (see Materials and Methods). We classified these transcripts into different categories based on the regulatory type of expression differences (see Materials and Methods; Figs. 1D, 3A, B and Supplementary Figs. S9–S11). Consistent with previous findings in hybrid mouse and chicken [12–14], the majority of expressed transcripts showed evidence in conserved (60.17%–74.85% of PCGs, 74.68%–84.22% of lncRNAs, and 67.63%–77.5% of TUCPs for each tissue) or ambiguous (18.21%–24.96% of PCGs, 12.96%–20.58% of lncRNAs, and 18.17%–25.19% of TUCPs for each tissue) regulatory patterns (Fig. 3A, B and Supplementary Figs. S9–S11).

Notably, we observed a significantly higher number of *trans-*regulatory PCGs (ranging from 230 to 1,594, ∼831) compared to *cis*-regulatory PCGs (ranging from 116 to 235, ∼177) (*P* = 0.008, paired Student's *t* test; Fig. 3C), suggesting the dominant contribution of the former to PCG expression changes between breeds. However, an opposite trend was found in lncRNAs (∼88.86 of *cis* vs. ∼30.42 of *trans, P* = 0.008, paired Student's *t* test; Fig. 3D) and TUCPs (∼21.29 of *cis* vs. 9.57 of *trans, P* = 0.03, paired Student's *t* test; Fig. 3E), indicating the mechanisms responsible for expression changes differ across transcript types.

### *Trans-*regulatory effects underline adaptive phenotypic divergence between 2 pig breeds

The higher number of *trans-*regulated PCGs (Fig. 3C) suggests their significant contribution to shaping the observed phenotypic differences between pig breeds. These *trans*-regulated PCGs may be subject to strong selective pressures. Notably, Tibetan pigs exhibited remarkable adaptations in the spleen tissue to overcome hypoxia in highland environments, as evidenced by enhanced erythrocyte functions. Enriched pathways associated with this adaptation include “heme metabolic process,” “erythrocyte differentiation,” “transferrin transport,” and “regulation of hemoglobin biosynthetic process” (Fig. 3F). Additionally, the effects of hypoxia extend beyond erythrocyte functions and impact mitochondria and endoplasmic reticulum functions [36], as reflected by changes in “protein folding,” “protein folding in endoplasmic reticulum,” “protein targeting to ER,” “protein maturation,” “protein export,” and “mitochondrial translation” (Supplementary Fig. S12).

In contrast, Berkshire pigs exhibit evidence of long-term artificial selection. Notably, in the skeletal muscle tissue, which is associated with major commercial traits such as pork yield and quality, enriched pathways are primarily involved in muscle growth and hypertrophy, including “response to insulin,” “cellular response to nutrient levels,” “AMPK signaling pathway,” and “mTOR signaling pathway” (Fig. 3G). In addition, industrially farmed Berkshire pigs are consistently exposed to environmental xenobiotics (e.g., chemicals and drugs) through food, water, and air. As a result, they have developed a heightened immune response, as indicated by enriched pathways such as “regulation of defense response,” “response to type I interferon,” “response to dexamethasone,” “response to bacterium,” and “regulation of viral entry into host cell” (Supplementary Fig. S12).

In addition, we also found that the *trans-*regulatory PCGs showed significantly higher expression levels in the testable tissues than others (Fig. 3H, I and Supplementary Fig. S13), indicating their important role in tissue function.

### *Trans-*regulatory effects in skeletal muscle were associated with different cell types

**Figure 4:**
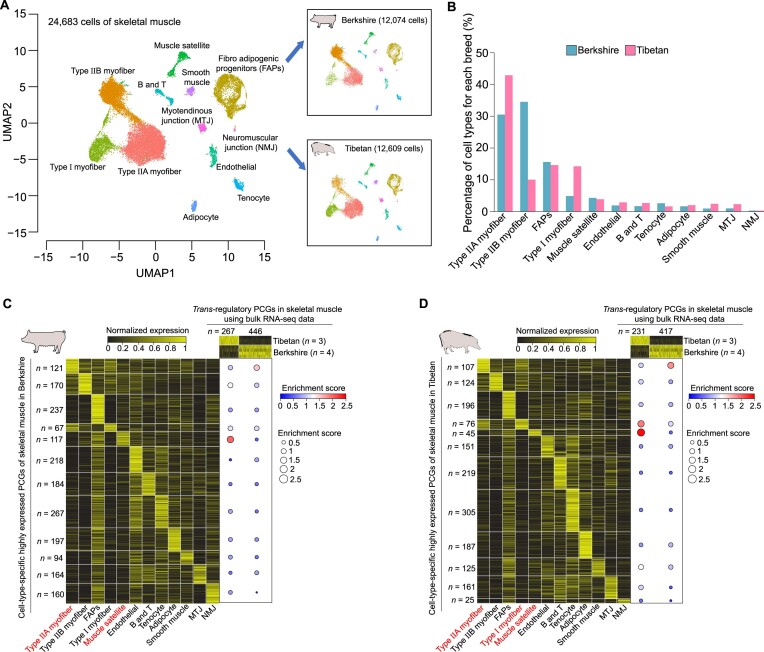
The correlation between *trans-*regulatory effects and cell type composition in skeletal muscle. (A) Cell type identification using UMAP clustering (left) and the projected coordination in Berkshire pigs (right, top) and Tibetan pigs (right, bottom). (B) The proportion of cells for each cell type in Berkshire pigs (blue, bars) and Tibetan pigs (pink, bars). (C, D) The relationship between cell type–specific highly expressed PCGs and *trans-*regulatory effects in skeletal muscle. An enrichment score was calculated to evaluate whether the *trans-*regulatory PCGs are enriched in cell type–specific highly expressed PCGs in Berkshire pigs (C) or Tibetan pigs (D).

Mammalian skeletal muscles are heterogeneous, composed of different myofibers and other cell types [37]. To query whether certain cell types contribute more to *trans-*regulatory effects in skeletal muscle, we performed single-nucleus RNA-seq from this tissue on purebred Berkshire and Tibetan pigs and harvested the transcriptome of 24,683 cells (with 12,074 from Berkshire pigs and 12,609 from Tibetan pigs; Fig. 4A). These cells were then subdivided into 12 distinct cell types, which exhibited different composition between breeds, especially for the 3 myofibers (types Ⅰ, ⅡA, and ⅡB; Fig. 4A, B and Supplementary Fig. S14).

We next sought to investigate whether distinct cell type composition was associated with *trans-*regulatory differences in skeletal muscle between breeds. We detected 67 to 267 (∼166.33) and 25 to 305 (∼143.42) cell type–specific highly expressed PCGs in Berkshire and Tibetan pigs, respectively (see Materials and Methods; Fig. 4C, D). Notably, we found the *trans-*regulatory PCGs of skeletal muscle with higher expression in Tibetan pigs were specifically enriched in cell type–specific highly expressed PCGs of type Ⅰ myofibers and muscle satellite (Fig. 4C, D). These observations suggest a major contribution of the 2 cell types to the enhanced *trans-*regulatory effects in skeletal muscle of Tibetan pigs. Type Ⅰ (slow-twitch or oxidative) myofibers can accelerate lipid accumulation in skeletal muscle [38, 39], providing enhanced athletic performance and a substantial energy source to survive in high-altitude environments characteristic of Tibet. Muscle satellite cells are resident stem cells from skeletal muscle that are activated by hypoxia niches to regulate proliferation and differentiation of myofibers during muscle regeneration [40, 41], and they are beneficial for the survival of Tibetan pigs. In contrast, the higher expression of *trans-*regulatory PCGs of skeletal muscle in Berkshire pigs was specifically enriched in cell type–specific highly expressed PCGs of type ⅡA myofibers (fast-twitch or oxidative/glycolytic; Fig. 4C, D). These myofibers can increase the ability to metabolize carbohydrates for rapid growth while reducing pH, intramuscular fat content, and water-holding capacity that impairs pork quality [38, 39].

Taken together, these results demonstrate that *trans-*regulatory effects in skeletal muscle result from different cell type composition and reveal the potential contribution of different cell types to expression differences between breeds.

### *Cis-*regulatory effects exhibited greater sequence divergence

**Figure 5:**
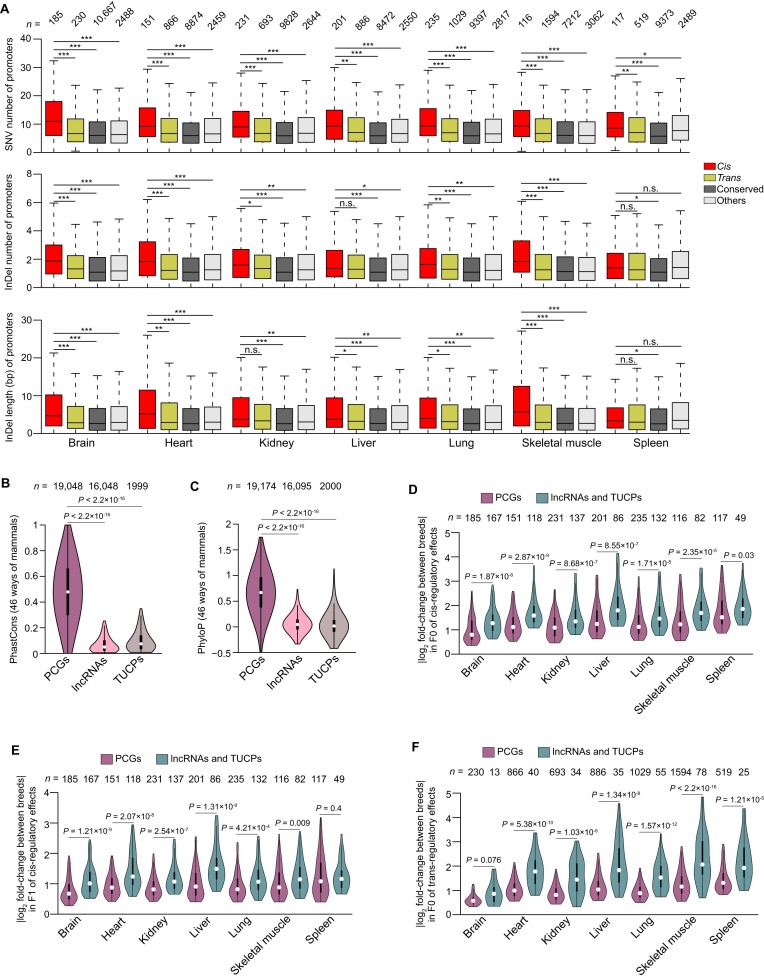
*Cis*-regulatory effects showing greater sequence divergence. (A) Boxplots showing the distribution of SNV number (top), indel number (middle), and indel length (bottom) for each promoter of PCG among the different regulatory categories of *cis* (red), *trans* (yellow), conserved (gray), and others (white). Median of 9.25 SNVs in *cis* vs. 6.83 in *trans*, 5.92 in conserved and 6.58 in others, *P* < 0.05 for all tissues; median of 1.63 indels in *cis* vs. 1.29 in *trans*, 1.08 in conserved and 1.25 in others, *P* < 0.05 except for liver and spleen; median of 4.17 bp indels in *cis* vs. 3.04 in *trans*, 2.58 in conserved and 2.96 in others, *P* < 0.05 except for kidney and spleen. (B, C) Violin plots showing the conservation level of PhastCons (B) and PhyloP (C) for the exonic regions of PCGs, lncRNAs, and TUCPs. PhastCons: median of 0.05 and 0.07 in lncRNAs and TUCPs vs. 0.48 in PCGs, *P* < 2.2 × 10^−16^; PhyloP: median of 0.05 and 0.02 in lncRNAs and TUCPs vs. 0.67 in PCGs, *P* < 2.2 × 10^−16^. (D–F) Violin plots showing the degree of expression divergence between breeds (i.e., the absolute log_2_ fold-change of expression between breeds) between PCGs and combined lncRNAs and TUCPs for *cis*-regulatory effects in F0 (D) and F1 groups (E), as well as for *trans*-regulatory effects in F0 (F). Median of 1.51 in combined lncRNAs and TUCPs vs. 1.17 in PCGs for F0 group, *P* < 0.03 for all tissues; median of 1.14 in combined lncRNAs and TUCPs vs. 0.83 in PCGs for F1 group, *P* < 0.009 except for spleen; median of 1.79 in combined lncRNAs and TUCPs vs. 1.01 in PCGs for F0 group, *P* < 1.21 × 10^−5^ except for brain. The number of testable transcripts for each category is shown (top) and the *P* values were calculated by Wilcoxon rank-sum test (n.s., *P* ≥ 0.05; *0.01 < *P* < 0.05; **0.001 < *P* < 0.01; ***P < 0.001).

While *trans-*regulatory effects are caused by mutations in diffusible components (e.g., transcription factors), *cis-*regulatory effects mainly result from genetic variants at *cis-*regulatory elements (typically, promoters and enhancers) [7, 12]. To investigate the association between genetic variants and *cis-*regulatory effects, we evaluated the interbreed sequence divergence in promoter sequences (i.e., 2-kb upstream regions of transcription start sites of PCGs, lncRNAs, and TUCPs) using the heterozygous SNVs and indels detected in each F1 progeny. As expected, we found a significantly higher occurrence of genetic variants in the promoter regions of *cis-*regulatory PCGs compared to the other regulatory categories considered (e.g., median of 9.25 SNVs in *cis* vs. 6.83 in *trans, P* < 0.05, Wilcoxon rank-sum test; Fig. 5A). A similar but weaker distribution was also found in lncRNAs and TUCPs (Supplementary Fig. S15). Furthermore, our findings were further supported by the observation of a similar distribution of variants when utilizing the H3K4me3 peak-annotated promoters in the pig genome (Supplementary Fig. S16) [42]. This additional analysis provides further validation and reinforces the robustness of our results.

Previous studies in mouse [13] and chicken [12] have reported that *cis*-regulated genes exhibit lower conservation levels. Consistent with these findings, we also observed similar results in our study. Specifically, we found significantly lower conservation scores for genes regulated by *cis*-elements (e.g., median PhastCons of 0.42 for exonic regions in *cis* vs. 0.5 in *trans, P* < 0.001, Wilcoxon rank-sum test; Supplementary Fig. S17A, B). Additionally, we observed larger dN/dS ratios between pig orthologs and their human and mouse counterparts for *cis*-regulated genes (e.g., median ratio of 0.14 in *cis* vs. 0.11 in *trans, P* < 0.05, Wilcoxon rank-sum test; Supplementary Fig. S17C, D).

Since the 3 transcript types showed distinct conservation levels (Fig. 5B, C), we next compared the degree of *cis-*regulatory effects on expression differences (i.e., the absolute log_2_ fold-change of expression between breeds) between transcript types. As expected, lncRNAs and TUCPs showed much lower sequence conservation (e.g., for PhastCons, median of 0.05 and 0.07 in lncRNAs and TUCPs vs. 0.48 in PCGs, *P* < 2.2 × 10^−16^, Wilcoxon rank-sum test; Fig. 5B, C) and exhibited a significantly greater expression divergence between breeds compared to PCGs (e.g., median of 1.51 in combined of lncRNAs and TUCPs vs. 1.17 in PCGs for F0 group, *P* < 0.03, Wilcoxon rank-sum test; Fig. 5D, E). This was also observed in *trans-*regulatory effects (Fig. 5F) despite small lncRNA and TUCP numbers.

### Most *cis-* and *trans-*regulatory effects are tissue specific

**Figure 6:**
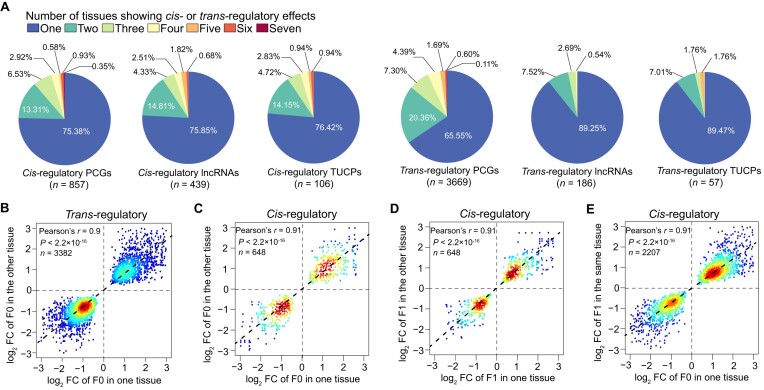
The tissue-specific patterns of *cis-* and *trans-*regulatory effects. (A) Pie charts showing the number of tissues with *cis-* or *trans-*regulatory effects for PCGs, lncRNAs, and TUCPs. (B–D) Scatter heatmaps showing the Pearson's correlation of log_2_ expression fold-change (Berkshire/Tibetan) for the same regulatory status between tissue pairs, for *trans-*regulatory effects in the F0 group (B) and *cis-*regulatory effects in the F0 (C) and F1 (D) groups. (E) Scatter heatmap showing the Pearson's correlation of log_2_ expression fold-change (Berkshire/Tibetan) between the F0 and F1 groups within the same tissue in *cis-*regulatory effects.

Recent studies have suggested that regulatory effects on gene expression vary across different tissues [43]. Similarly, we found that most *cis-*regulatory effects (75.38% of PCGs, 75.85% of lncRNAs, and 76.42% of TUCPs) and *trans-*regulatory effects (65.55% of PCGs, 89.25% of lncRNAs, and 89.47% of TUCPs) were detected in a single tissue (Fig. 6A), highlighting a tissue-dependent response to the same regulatory variants. Interestingly, transcripts with the same regulatory pattern in 2 or more tissues exhibited similar levels of expression differences between breeds (i.e., the log_2_ fold-change expression of Berkshire to Tibetan), as shown by the high correlation coefficient of *trans-*regulatory effects in the F0 group (Pearson's *r* = 0.9, *n* = 3,382 tissue pairs, *P* < 2.2 × 10^−16^; Fig. 6B), as well as *cis-*regulatory effects in both the F0 (Pearson's *r* = 0.91, *n* = 648 tissue pairs, *P* < 2.2 × 10^−16^; Fig. 6C) and F1 (Pearson's *r* = 0.91, *n* = 648 tissue pairs, *P* < 2.2 × 10^−16^; Fig. 6D) groups. This is similar to the correlation observed in *cis-*regulatory effects between F0 and F1 groups within the same tissue (Pearson's *r* = 0.91, *n* = 2,007 F0–F1 pairs, *P* < 2.2 × 10^−16^; Fig. 6E), indicating that the same sequence variants in pleiotropic regulatory elements may cause similar differential expression between breeds among different tissues [44].

## Discussion

A reciprocal cross-strategy has been widely used to analyze allele-specific expression patterns [3, 6, 12, 13]. Using Berkshire and Tibetan pigs, we generated a reciprocal cross-model to investigate the allele-specific regulatory effects on the pig transcriptome. We detected a relatively high ratio of heterozygous SNVs in F1 progenies that reached ∼4.64 heterozygous SNVs per kb. This is still lower than hybrid mouse (∼7.7 per kb) but comparable to hybrid fruit flies (∼5 per kb) and higher than humans (∼1 per kb) [45, 46]. In general, this high heterozygous ratio demonstrates European and Asian pigs, like Berkshire and Tibetan, are ideal to build reciprocal cross-models to study allele-specific expression differences between breeds. Berkshire pigs represent the typical European commercial pig breed that underwent strong artificial selection for greater pork yield, larger body size, and higher growth rate than Tibetan pigs.

Imprinted genes play important roles in viability, development, growth, and various other physiological functions [5]. Previously, approximately 200 imprinted PCGs and lncRNAs were identified in the human and mouse genomes [5]. Despite several studies being conducted to identify imprinted genes in the pig genome [47–49], until now, only 36 had been reported. In addition, no imprinted noncoding transcripts (except H19) had been detected due to the poor annotation of the reference pig genome. This study aims to offer a comprehensive annotation of putative imprinted PCGs, lncRNAs, and TUCPs in the pig genome.

The occurrence of *trans-*regulatory variants influences the expression of a large number of genes [7]. To maintain stable gene expression, other regulatory components may act in a compensatory way to eliminate the most deleterious *trans-*acting variants [7, 14]. Hence, *trans-*regulatory expression divergence between breeds may have evolved under strong selective pressures, especially for agricultural domesticated animals [12, 13]. We found a notable disparity in the proportion of *trans*-regulated effects compared to *cis*-regulated effects in PCGs (Fig. 3C). This observation indicates that *trans-*regulated effects may have a more prominent role in shaping the distinct phenotypes observed between breeds. The enrichment of functional pathways further supports this notion, highlighting the significance of *trans*-regulated effects in driving breed-specific traits and characteristics.

## Supplementary Material

giad076_GIGA-D-23-00059_Original_Submission

giad076_GIGA-D-23-00059_Revision_1

giad076_Response_to_Reviewer_Comments_Original_Submission

giad076_Reviewer_1_Report_Original_Submission Xuewen Xu -- 4/8/2023 Reviewed

giad076_Reviewer_1_Report_Revision_1 Xuewen Xu -- 7/15/2023 Reviewed

giad076_Reviewer_2_Report_Original_Submission Martien Groenen -- 4/11/2023 Reviewed

giad076_Supplemental_File

## Data Availability

The whole-genome sequencing data (*n* = 22) and rRNA-depleted RNA-seq data (*n* = 199) are available at the Genome Sequence Archive with GSA accession CRA009370. The sequence data associated with this study are deposited in NCBI BioProject PRJNA998604. All additional supporting data are available in the *GigaScience* GigaDB database [50].

## Additional Files

**Supplementary Fig. S1**. Summary of rRNA-depleted RNA-seq data of F1 progenies.

**Supplementary Fig. S2**. Classification of expression regulatory categories between breeds.

**Supplementary Fig. S3**. Whole-genome sequencing and SNV calling.

**Supplementary Fig. S4**. Phasing of heterozygous SNVs and assignment of expression.

**Supplementary Fig. S5**. Identification of imprinted PCGs.

**Supplementary Fig. S6**. Identification of imprinted lncRNAs.

**Supplementary Fig. S7**. Identification of imprinted TUCPs.

**Supplementary Fig. S8**. Summary of rRNA-depleted RNA-seq data of purebred Berkshire and Tibetan pigs.

**Supplementary Fig. S9**. Classification of *cis-* and *trans-*regulatory effects of PCGs.

**Supplementary Fig. S10**. Classification of *cis-* and *trans-*regulatory effects of lncRNAs.

**Supplementary Fig. S11**. Classification of *cis-* and *trans-*regulatory effects of TUCPs.

**Supplementary Fig. S12**. GO and KEGG enrichment for *trans*-regulatory PCGs with higher expression in Berkshire (blue) and Tibetan (pink) pigs for each tissue.

**Supplementary Fig. S13**. The TPM ranks of each tissue for *trans*-regulatory PCGs.

**Supplementary Fig. S14**. Classification of different cell types.

**Supplementary Fig. S15**. Distribution of SNV number, indel number, and indel length of promoters in combined lncRNAs and TUCPs.

**Supplementary Fig. S16**. Distribution of the density for SNV number (top), indel number (middle), and indel length (bp, bottom) of the peak-annotated promoters in PCGs.

**Supplementary Fig. S17**. The conservation comparison between different regulatory categories for PCGs.

**Supplementary Data S1**. Sample description.

**Supplementary Data S2**. Putative imprinted transcripts.

## Abbreviations

ASE: allele-specific expression; bp: base pair; GATK: Genome Analysis Toolkit; Gb: gigabase; indel: insertion and deletion; GO: Gene Ontology; kb: kilobase; KEGG: Kyoto Encyclopedia of Genes and Genomes; lncRNA: long noncoding RNA; PBS: phospahte-buffered saline; PCG: protein-coding gene; rRNA: ribosomal RNA; SNV: single-nucleotide variant; TPM: transcripts per million; TUCP: transcript of unknown coding potential; UMAP: Uniform Manifold Approximation and Projection.

## References

[1] Andergassen D, Dotter CP, Kulinski TM, et al. Allelome.PRO, a pipeline to define allele-specific genomic features from high-throughput sequencing data. Nucleic Acids Res. 2015;43(21):gkv727. 10.1093/nar/gkv727. PMC4666383 26202974

[2] St Pierre CL, Macias-Velasco JF, Wayhart JP, et al. Genetic, epigenetic, and environmental mechanisms govern allele-specific gene expression. Genome Res. 2022;32(6):1042–57.. 10.1101/gr.276193.121. 35501130 PMC9248887

[3] Andergassen D, Dotter CP, Wenzel D, et al. Mapping the mouse Allelome reveals tissue-specific regulation of allelic expression. eLife. 2017;6:e25125. 10.7554/eLife.25125. 28806168 PMC5555720

[4] Santini L, Halbritter F, Titz-Teixeira F, et al. Genomic imprinting in mouse blastocysts is predominantly associated with H3K27me3. Nat Commun. 2021;12(1):3804. 10.1038/s41467-021-23510-4. 34155196 PMC8217501

[5] Tucci V, Isles AR, Kelsey G, et al. Genomic imprinting and physiological processes in mammals. Cell. 2019;176(5):952–65.. 10.1016/j.cell.2019.01.043. 30794780

[6] Metzger BPH, Duveau F, Yuan DC, et al. Contrasting frequencies and effects of cis- and trans-regulatory mutations affecting gene expression. Mol Biol Evol. 2016;33(5):1131–46.. 10.1093/molbev/msw011. 26782996 PMC4909133

[7] Signor SA, Nuzhdin SV. The evolution of gene expression in cis and trans. Trends Genet. 2018;34(7):532–44.. 10.1016/j.tig.2018.03.007. 29680748 PMC6094946

[8] Crowley JJ, Zhabotynsky V, Sun W, et al. Analyses of allele-specific gene expression in highly divergent mouse crosses identifies pervasive allelic imbalance. Nat Genet. 2015;47(4):353–60.. 10.1038/ng.3222. 25730764 PMC4380817

[9] Schaefke B, Emerson JJ, Wang TY, et al. Inheritance of gene expression level and selective constraints on trans- and cis-regulatory changes in yeast. Mol Biol Evol. 2013;30(9):2121–33.. 10.1093/molbev/mst114. 23793114

[10] Fear JM, Leon-Novelo LG, Morse AM, et al. Buffering of genetic regulatory networks in Drosophila melanogaster. Genetics. 2016;203(3):1177–90.. 10.1534/genetics.116.188797. 27194752 PMC4937466

[11] McManus CJ, Coolon JD, Duff MO, et al. Regulatory divergence in Drosophila revealed by mRNA-seq (vol 20, pg 816, 2010). Genome Res. 2014;24(6):1051. 10.1101/gr.102491.109. PMC2877578 20354124

[12] Wang Q, Jia YX, Wang Y, et al. Evolution of cis- and trans-regulatory divergence in the chicken genome between two contrasting breeds analyzed using three tissue types at one-day-old. BMC Genomics. 2019;20(1):933. 10.1186/s12864-019-6342-5. 31805870 PMC6896592

[13] Goncalves A, Leigh-Brown S, Thybert D, et al. Extensive compensatory cis-trans regulation in the evolution of mouse gene expression. Genome Res. 2012;22(12):2376–84.. 10.1101/gr.142281.112. 22919075 PMC3514667

[14] Mack KL, Campbell P, Nachman MW. Gene regulation and speciation in house mice. Genome Res. 2016;26(4):451–61.. 10.1101/gr.195743.115. 26833790 PMC4817769

[15] Lin Y, Li J, Gu YR, et al. Allele-specific effects of three-dimensional genome architecture in hybrid pigs. 2022. Preprint available at Research Square. 10.21203/rs.3.rs-2392032/v2.

[16] Jin L, Tang QZ, Hu SL, et al. A pig BodyMap transcriptome reveals diverse tissue physiologies and evolutionary dynamics of transcription. Nat Commun. 2021;12(1):3715. 10.1038/s41467-021-23560-8. 34140474 PMC8211698

[17] Bray NL, Pimentel H, Melsted P, et al. Near-optimal probabilistic RNA-seq quantification. Nat Biotechnol. 2016;34(5):525–7.. 10.1038/nbt.3519. 27043002

[18] Li H, Durbin R. Fast and accurate short read alignment with Burrows-Wheeler transform. Bioinformatics. 2009;25(14):1754–60.. 10.1093/bioinformatics/btp324. 19451168 PMC2705234

[19] McKenna A, Hanna M, Banks E, et al. The Genome Analysis Toolkit: a MapReduce framework for analyzing next-generation DNA sequencing data. Genome Res. 2010;20(9):1297–303.. 10.1101/gr.107524.110. 20644199 PMC2928508

[20] Yang J, Lee SH, Goddard ME, et al. GCTA: a tool for genome-wide complex trait analysis. Am Hum Genet. 2011;88(1):76–82.. 10.1016/j.ajhg.2010.11.011. PMC3014363 21167468

[21] Pritchard JK, Stephens M, Donnelly P. Inference of population structure using multilocus genotype data. Genetics. 2000;155(2):945–59.. 10.1093/genetics/155.2.945. 10835412 PMC1461096

[22] Choi Y, Chan AP, Kirkness E, et al. Comparison of phasing strategies for whole human genomes. PLoS Genet. 2018;14(4):e1007308. 10.1371/journal.pgen.1007308. 29621242 PMC5903673

[23] Liang Z, Myers ZA, Petrella D, et al. Mapping responsive genomic elements to heat stress in a maize diversity panel. Genome Biol. 2022;23(1):234. 10.1186/s13059-022-02807-7. 36345007 PMC9639295

[24] Wang W, Yang Y, Tan S, et al. Genomic imprinting-like monoallelic paternal expression determines sex of channel catfish. Sci Adv. 2022;8(51):eadc8786. 10.1126/sciadv.adc8786. 36542716 PMC9770954

[25] Dobin A, Davis CA, Schlesinger F, et al. STAR: ultrafast universal RNA-seq aligner. Bioinformatics. 2013;29(1):15–21.. 10.1093/bioinformatics/bts635. 23104886 PMC3530905

[26] Krueger F, Andrews SR. SNPsplit: allele-specific splitting of alignments between genomes with known SNP genotypes. F1000Res. 2016;5:1479. 10.12688/f1000research.9037.2. 27429743 PMC4934512

[27] Love MI, Huber W, Anders S. Moderated estimation of fold change and dispersion for RNA-seq data with DESeq2. Genome Biol. 2014;15(12):550. 10.1186/s13059-014-0550-8. 25516281 PMC4302049

[28] Hao Y, Hao S, Andersen-Nissen E, et al. Integrated analysis of multimodal single-cell data. Cell. 2021;184(13):3573–87..e29. 10.1016/j.cell.2021.04.048. 34062119 PMC8238499

[29] Korsunsky I, Millard N, Fan J, et al. Fast, sensitive and accurate integration of single-cell data with Harmony. Nat Methods. 2019;16(12):1289–96.. 10.1038/s41592-019-0619-0. 31740819 PMC6884693

[30] Becht E, McInnes L, Healy J, et al. Dimensionality reduction for visualizing single-cell data using UMAP. Nat Biotechnol. 2019;37(1):38–44.. 10.1038/nbt.4314. 30531897

[31] Edgar RC . MUSCLE: multiple sequence alignment with high accuracy and high throughput. Nucleic Acids Res. 2004;32(5):1792–7.. 10.1093/nar/gkh340. 15034147 PMC390337

[32] Zhang Z . KaKs_Calculator 3.0: calculating selective pressure on coding and non-coding sequences. Genomics Proteomics Bioinformatics. 2022;20(3):536–40.. https://doi.10.1016/j.gpb.2021.12.002. 34990803 10.1016/j.gpb.2021.12.002 PMC9801026

[33] Zhou Y, Zhou B, Pache L, et al. Metascape provides a biologist-oriented resource for the analysis of systems-level datasets. Nat Commun. 2019;10(1):1523. 10.1038/s41467-019-09234-6. 30944313 PMC6447622

[34] Weinberg-Shukron A, Ben-Yair R, Takahashi N, et al. Balanced gene dosage control rather than parental origin underpins genomic imprinting. Nat Commun. 2022;13(1):12. 10.1038/s41467-022-32144-z. 35906226 PMC9338321

[35] Lautem A, Heise M, Gräsel A, et al. Downregulation of organic cation transporter 1 (*SLC22A1*) is associated with tumor progression and reduced patient survival in human cholangiocellular carcinoma. Int J Oncol. 2013;42(4):1297–304.. 10.3892/ijo.2013.1840. 23440379

[36] Bartoszewska S, Collawn JF. Unfolded protein response (UPR) integrated signaling networks determine cell fate during hypoxia. Cell Mol Biol Lett. 2020;25:18. https://doi.10.1186/s11658-020-00212-1. 32190062 10.1186/s11658-020-00212-1 PMC7071609

[37] Petrany MJ, Swoboda CO, Sun CY, et al. Single-nucleus RNA-seq identifies transcriptional heterogeneity in multinucleated skeletal myofibers. Nat Commun. 2020;11(1):6374. 10.1038/s41467-020-20063-w. 33311464 PMC7733460

[38] Ryu YC, Kim BC. The relationship between muscle fiber characteristics, postmortem metabolic rate, and meat quality of pig longissimus dorsi muscle. Meat Sci. 2005;71(2):351–7.. 10.1016/j.meatsci.2005.04.015. 22064236

[39] Zhao L, Huang Y, Du M. Farm animals for studying muscle development and metabolism: dual purposes for animal production and human health. Anim Front. 2019;9(3):21–7.. 10.1093/af/vfz015. 32002259 PMC6952012

[40] Pircher T, Wackerhage H, Aszodi A, et al. Hypoxic signaling in skeletal muscle maintenance and regeneration: a systematic review. Front Physiol. 2021;12:684899. 10.3389/fphys.2021.684899. 34248671 PMC8260947

[41] Liu WY, Wen YF, Bi PP, et al. Hypoxia promotes satellite cell self-renewal and enhances the efficiency of myoblast transplantation. Development. 2012;139(16):2857–65.. 10.1242/dev.079665. 22764051 PMC3403098

[42] Pan Z, Yao Y, Yin H, et al. Pig genome functional annotation enhances the biological interpretation of complex traits and human disease. Nat Commun. 2021;12(1):5848. 10.1038/s41467-021-26153-7. 34615879 PMC8494738

[43] Mugal CF, Wang M, Backstrom N, et al. Tissue-specific patterns of regulatory changes underlying gene expression differences among Ficedula flycatchers and their naturally occurring F 1 hybrids. Genome Res. 2020;30(12):1727–39.. 10.1101/gr.254508.119. 33144405 PMC7706733

[44] Shen SQ, Turro E, Corbo JC. Hybrid mice reveal parent-of-origin and cis- and trans-regulatory effects in the retina. PLoS One. 2014;9(10):e109382. 10.1371/journal.pone.0109382. 25340786 PMC4207689

[45] de Wit E . Capturing heterogeneity: single-cell structures of the 3D genome. Nat Struct Mol Biol. 2017;24(5):437–8.. https://doi.10.1038/nsmb.3404. 28471429 10.1038/nsmb.3404

[46] Abed JA, Erceg J, Goloborodko A, et al. Highly structured homolog pairing reflects functional organization of the Drosophila genome. Nat Commun. 2019;10(1):4485. https://doi.10.1038/s41467-019-12208-3. 31582763 10.1038/s41467-019-12208-3 PMC6776532

[47] Hou X, Wang Z, Shi L, et al. Identification of imprinted genes in the skeletal muscle of newborn piglets by high-throughput sequencing. Anim Genet. 2022;53(4):479–86.. 10.1111/age.13212. 35481679

[48] Yu D, Wang J, Zou H, et al. Silencing of retrotransposon-derived imprinted gene RTL1 is the main cause for postimplantational failures in mammalian cloning. Proc Natl Acad Sci USA. 2018;115(47):E11071–e80.. 10.1073/pnas.1814514115. 30381455 PMC6255163

[49] Bischoff SR, Tsai S, Hardison N, et al. Characterization of conserved and nonconserved imprinted genes in swine. Biol Reprod. 2009;81(5):906–20.. 10.1095/biolreprod.109.078139. 19571260 PMC2770020

[50] Lin Y, Li J, Chen L, et al. Supporting data for “Allele-Specific Regulatory Effects on the Pig Transcriptome.”. GigaScience Database. 2023. 10.5524/102427. PMC10541795 37776365

