# Supplementary material for: Allele-specific regulatory effects on the pig transcriptome
Source: Gigascience. 2023 Sep 30;12:giad076. doi: 10.1093/gigascience/giad076 (PMC10541795; doi:10.1093/gigascience/giad076)
Supplement: giad076_GIGA-D-23-00059_Original_Submission [file giad076_giga-d-23-00059_original_submission.pdf]

GigaScience

Allele-specific regulatory effects on pig transcriptome

--Manuscript Draft--

**Manuscript Number:**	GIGA-D-23-00059
**Full Title:**	Allele-specific regulatory effects on pig transcriptome
**Article Type:**	Research

Funding Information:

National Key R & D Program of China (2022YFF1000100)	Dr Jing Li
National Key R & D Program of China (2021YFD1301102)	Dr Li Chen
Tackling Project for Agricultural Key Core Technologies of China (NK2022110602)	Prof Long Jin
National Natural Science Foundation of China (32102507)	Dr Jing Li
National Natural Science Foundation of China (U22A20507)	Dr Jing Li
National Natural Science Foundation of China (32072687)	Prof Liangpeng Ge
National Natural Science Foundation of China (32225046)	Prof. Mingzhou Li
Sichuan Science and Technology Program (2022NSFSC1618)	Dr Jing Li
Sichuan Science and Technology Program (2021ZDZX0008)	Dr Keren Long
Major Science and Technology Projects of Tibet Autonomous Region (XZ202101ZD0005N)	Prof. Mingzhou Li

Abstract:

Background: Allele-specific expression (ASE) refers to the preferentially expression of one allele over the other and contributes to adaptive phenotypic plasticity. Here, we used a reciprocal cross model between phenotypically divergent European Berkshire and Asian Tibetan pigs to characterize two ASE classes: imprinting (i.e., the unequal expression between parental alleles) and sequence-dependent (i.e., unequal expression between breed-specific alleles). We examined three transcript types, including protein-coding genes (PCGs), long non-coding RNAs (lncRNAs) and transcripts of unknown coding potential (TUCPs) across seven representative somatic tissues from hybrid pigs generated by reciprocal crosses.

Results: We identified a total of 92 putative imprinted transcripts, 69 (75.00%) of which are described here for the first time. By combining the transcriptome from purebred Berkshire and Tibetan pigs, we found ~6.59% of PCGs are differentially expressed between breeds that regulated by trans-elements (e.g., transcriptional factors) while only ~1.35% are attributable to cis (e.g., promoters). The higher prevalence of trans-PCGs indicate the dominated effects of trans-regulation in driving expression differences and shaping adaptive phenotypic plasticity between breeds, which were supported by functional enrichment analysis. We also found strong evidence that expression changes mediated by cis-effects were associated with accumulated variants in promotes.

Conclusions: Our study provides comprehensive map of expression regulation that constitutes a valuable resource for the agricultural improvement of pig breeds.

Corresponding Author:

Mingzhou Li, Ph.D.
Sichuan Agricultural University
Chengdu, Sichuan CHINA

Corresponding Author Secondary Information:

**Corresponding Author's Institution:**	Sichuan Agricultural University
**Corresponding Author's Secondary Institution:**	
**First Author:**	Yu Lin
**First Author Secondary Information:**	
**Order of Authors:**	Yu Lin
	Jing Li
	Li Chen
	Jingyi Bai
	Jiaman Zhang
	Yujie Wang
	Pengliang Liu
	Keren Long
	Liangpeng Ge
	Long Jin
	Yiren Gu
	Mingzhou Li, Ph.D.
**Order of Authors Secondary Information:**	
**Additional Information:**	
**Question**	**Response**
Are you submitting this manuscript to a special series or article collection?	No
**Experimental design and statistics**<br><br>Full details of the experimental design and statistical methods used should be given in the Methods section, as detailed in our Minimum Standards Reporting Checklist. Information essential to interpreting the data presented should be made available in the figure legends.<br><br>Have you included all the information requested in your manuscript?	Yes
**Resources**<br><br>A description of all resources used, including antibodies, cell lines, animals and software tools, with enough information to allow them to be uniquely identified, should be included in the	Yes

Powered by Editorial Manager® and ProduXion Manager® from Aries Systems Corporation

Methods section. Authors are strongly encouraged to cite Research Resource Identifiers (RRIDs) for antibodies, model organisms and tools, where possible.<br><br>Have you included the information requested as detailed in our Minimum Standards Reporting Checklist?	
**Availability of data and materials**<br><br>All datasets and code on which the conclusions of the paper rely must be either included in your submission or deposited in publicly available repositories (where available and ethically appropriate), referencing such data using a unique identifier in the references and in the "Availability of Data and Materials" section of your manuscript.<br><br>Have you have met the above requirement as detailed in our Minimum Standards Reporting Checklist?	Yes

Manuscript Click here to access/download;Manuscript;1. Lin et al. Manuscript.docx

Allele-specific regulatory effects on pig transcriptome

Yu Lin[1,†], Jing Li[1,2,†,*], Li Chen[3,4,†], Jingyi Bai[1], Jiaman Zhang[1], Yujie Wang[1], Pengliang Liu[1], Keren Long[2], Liangpeng Ge[3,4], Long Jin[2], Yiren Gu[5,6] and Mingzhou Li[1,*]

[1]Livestock and Poultry Multi-omics Key Laboratory of Ministry of Agriculture and Rural Affairs, College of Animal Science and Technology, Sichuan Agricultural University, Chengdu, 611130, China.

[2]Animal Breeding and Genetics Key Laboratory of Sichuan Province, Institute of Animal Genetics and Breeding, Sichuan Agricultural University, Chengdu, 611130, China.

[3]Pig Industry Sciences Key Laboratory of Ministry of Agriculture and Rural Affairs, Chongqing Academy of Animal Sciences, Chongqing, 402460, China.

[4]National Center of Technology Innovation for Pigs, Chongqing, 402460, China.

[5]College of Animal and Veterinary Sciences, Southwest Minzu University, Chengdu, 610041, China.

[6]Animal Breeding and Genetics Key Laboratory of Sichuan Province, Sichuan Animal Science Academy, Chengdu, 610066, China.

[†]These authors contributed equally.

[*]Correspondence address: Jing Li. Animal Breeding and Genetics Key Laboratory of Sichuan Province, Institute of Animal Genetics and Breeding, Sichuan Agricultural University, Chengdu, 611130, China. E-mail: lijing_2020@sicau.edu.cn; Mingzhou Li. Livestock and Poultry Multi-omics Key Laboratory of Ministry of Agriculture and Rural Affairs, College of Animal Science and Technology, Sichuan Agricultural University, Chengdu, 611130, China. E-mail: mingzhou.li@sicau.edu.cn.

Abstract

Background: Allele-specific expression (ASE) refers to the preferentially expression of one allele over the other and contributes to adaptive phenotypic plasticity. Here, we used a reciprocal cross model between phenotypically divergent European Berkshire and Asian Tibetan pigs to characterize two ASE classes: imprinting (i.e., the unequal expression between parental alleles) and sequence-dependent (i.e., unequal expression between breed-specific alleles). We examined three transcript types, including protein-coding genes

(PCGs), long non-coding RNAs (lncRNAs) and transcripts of unknown coding potential (TUCPs) across seven representative somatic tissues from hybrid pigs generated by reciprocal crosses.

Results: We identified a total of 92 putative imprinted transcripts, 69 (75.00%) of which are described here for the first time. By combining the transcriptome from purebred Berkshire and Tibetan pigs, we found ~6.59% of PCGs are differentially expressed between breeds that regulated by *trans*-elements (e.g., transcriptional factors) while only ~1.35% are attributable to *cis* (e.g., promoters). The higher prevalence of *trans*-PCGs indicate the dominated effects of *trans*-regulation in driving expression differences and shaping adaptive phenotypic plasticity between breeds, which were supported by functional enrichment analysis. We also found strong evidence that expression changes mediated by *cis*-effects were associated with accumulated variants in promotes.

Conclusions: Our study provides comprehensive map of expression regulation that constitutes a valuable resource for the agricultural improvement of pig breeds.

Keywords: Allele-specific expression, imprinting, *cis*- and *trans*-regulatory effects, pig breeding

Background

Mammalian genomes are diploid and consist of two parental copies for each gene locus [1]. Allele-specific expression (ASE) occurs in diploid genomes when one allele is preferentially expressed over the other [2, 3]. Genetic and epigenetic differences between alleles are frequently associated with ASEs occurring at different developmental stages and leading to functional consequences on a variety of biological processes [2, 3].

ASEs can be classified into two distinct classes, namely imprinting and sequence-dependent [2]. The former refers to the broader class of epigenetic phenomena that depend on the preferential expression of either paternal or maternal alleles, and is closely associated with epigenetic markers, such as DNA methylation and histone modifications [2, 4]. To date, imprinted genes constitute approximately 1% of the human and mouse genomes [5]. In contrast, sequence-dependent ASEs represent the differential expression between alleles based on nucleotide similarity [2]. In most cases, they manifest as the

expression difference between breeds or strains within a species and are most likely driven through the action of *cis*-regulatory elements (typically, promoters and enhancers), which are functional non-coding DNA sequences that modulate the expression of nearby genes [6, 7]. In diploid individuals, genetic variants at *cis*-regulatory elements may induce differential expression between breeds or strains and can be inherited by F1 progenies [7, 8]. However, expression difference can also occur due to *trans*-acting factors, which are diffusible components (e.g., transcription factors) that affect the expression of distal genes by interacting with their target sequences [6, 7]. *Trans*-regulatory effects have an equal opportunity to influence both alleles and thus eliminate differential expression in F1 progenies [8].

Both *cis*- and *trans*-regulatory effects are responsible for adaptive phenotypic plasticity between breeds or strains. Recent studies used RNA-seq data and an allele-specific expression strategy to evaluate *cis*- and *trans*-regulatory effects in a wide range of species, including yeast [9], fruit fly [10, 11], chicken [12] and mouse [13, 14]. These studies identified a large number of single nucleotide variants (SNVs) in hybrid F1 progenies generated from reciprocal crosses between two genetically and phenotypically divergent breeds or strains. These heterozygous SNVs were then used to distinguish allele-specific RNA-seq reads and evaluate the expression difference between breeds or strains. By measuring the expression levels of the two parental breeds or strains, the authors identified different mechanisms of expression regulation, revealing the extensive adaptive evolutionary differences between them.

To investigate the allele-specific regulatory effects on pig transcriptome, we analyzed rRNA-depleted RNA-seq data across seven representative somatic tissues (brain, heart, kidney, liver, lung, skeletal muscle and spleen; n = 163) from hybrid F1 progenies (n = 24) generated by reciprocal crosses between Berkshire (originated in Europe) and Tibetan pigs (originated in China). We constructed allele-specific expression profiles for three transcript types (PCGs, lncRNAs and TUCPs) and identified 92 putative imprinted transcripts, 69 (75.00%) of which were novel. We also analyzed rRNA-depleted RNA-seq data for these seven tissues (n = 54) from purebred Berkshire (n = 4) and Tibetan pigs (n = 4), and uncovered a high number of *cis*- and *trans*-regulatory effects for each transcript type.

These results illustrate the regulatory mechanisms responsible for expression changes between pig breeds and provide new insights into their genetic breeding.

Materials and Methods

Sample collection

Reciprocal crosses were performed between Berkshire and Tibetan pigs, generating a total of eight families (four initial crosses and four reverse crosses; Fig. 1A and Supplementary Fig. S1A). We downloaded whole-genome sequencing data for 18 individuals (six parent-child trios; three initial and three reverse crosses [15]), along with rRNA-depleted RNA-seq data for three tissues of the six newly-born F1 females (including brain, liver and skeletal muscle, n = 18 libraries; Supplementary Fig. S1A and Data S1). We collected another four tissues (heart, kidney, lung and spleen) from the above F1 progenies to produce rRNA-depleted RNA-seq data. We produced 10 newly-born F1 progenies from parents of the aforementioned six families and collected samples of blood and other seven tissues (brain, heart, kidney, liver, lung, skeletal muscle and spleen) for each individual to generate whole-genome sequencing and rRNA-depleted RNA-seq data (Supplementary Fig. S1A and Data S1). In addition, we independently performed one initial cross (two parents with two F1 progenies) and one reverse cross (two parents with six F1 progenies), and again collected samples of blood and each of the aforementioned seven tissues from individuals of eight F1 progenies (Supplementary Fig. S1A and Data S1). To evaluate the expression levels of the parental breeds, we collected the aforementioned seven tissues from purebred Berkshire (n = 4) and Tibetan (n = 4) females. Finally, the skeletal muscle from additional purebred Berkshire and Tibetan pigs were collected to perform single-nucleus RNA-seq.

rRNA-depleted RNA-seq and data processing

We generated a total of 145 rRNA-depleted RNA-seq libraries across seven tissues of F1 progenies, as well as 54 rRNA-depleted RNA-seq libraries across seven tissues for purebred Berkshire and Tibetan pigs. Total RNA was extracted using the RNeasy Mini Kit (Qiagen). The rRNA depletion protocol (Ribo-Zero kit, Epicentre) and the Illumina TruSeq RNA-seq library protocol were then implemented to construct strand-specific RNA-seq libraries. The libraries were quantified using the Qubit dsDNA High Sensitivity Assay Kit (Invitrogen) and sequenced with 150-bp paired-end reads using the Illumina HiSeq X Ten

platform, or 100-bp paired-end reads using the BGISEQ-500 platform. The annotations of PCGs (Ensembl website, version *S.scrofa* 107) and two transcript types (lncRNAs and TUCPs, [16]) were integrated to build a complete transcript annotation in the pig genome. The Kallisto (version 0.44.0) software [17] was used to quantify the expression and count the read number of PCGs, lncRNAs and TUCPs using the 217 RNA-seq libraries (including 18 downloaded and 199 newly generated data).

Whole genome sequencing and variants calling

The genomic DNA was exacted from blood samples (n = 22) using the TIANamp Genomic DNA Kit (TIANGEN, DP304). Sequencing libraries were constructed and sequenced on the Illumina HiSeq X ten platform with 150-bp paired-end reads, or on the BGISEQ-500 platform with 100-bp paired-end reads. Low-quality and adapter sequencing data were removed, and the filtered sequences were mapped to the reference pig genome (version *S.scrofa* 11.1) using the Burrows-Wheeler Aligner software (BWA, version 0.7.8) with default parameters [18]. Potential PCR duplicates were removed using the module 'MarkDuplicates' of the Picard software (version 2.0.1; http://broadinstitute.github.io/picard). The module 'HaplotypeCaller' in software Genome Analysis Toolkit (GATK; version 4.2.6) [19] was used to detect SNVs, insertions and deletions (InDels) for each individual. For individuals within each family, the genetic variants were aggregated into a multi-sample VCF file using the module 'GVCFGenotyper' of GATK. Low-quality SNVs and InDels were discarded using the following conditions on GATK: 'QD < 10.0 || FS > 60.0 || MQ < 40.0 || MQRankSum <–12.5 || ReadPosRankSum <–8.0 || GQ < 30'. Unmapped scaffolds, sex chromosomes and the mitochondrial genome were also removed from further analyses. In addition, SNVs and InDels with very high or low coverage (defined as the top and bottom ~1% of the distribution, respectively, or a depth-based Z-score < –2.58 and > 2.58) were also discarded. The final SNV dataset was used to perform principal component analysis (PCA) using the software GCTA (version 1.93.2) [20] and infer genetic structure using the Structure software (version 2.3.4) [21].

Assignment of allele-specific RNA-seq reads

The heterozygous SNVs of each F1 progeny were phased using a trio-based strategy, when at least one of the parents was homozygous [22]. The phased SNVs were then used

to mask the reference pig genome and thus reduce mapping bias. The high-quality RNA-seq reads of hybrid F1 progenies (*n* = 163; including 18 downloaded and 145 newly generated) were aligned to the 'N'-masked reference genome using STAR (version 2.6.0c) [23] with default parameters. The software SNPsplit (version 0.3.4) [24] was used to distinguish the allele-specific RNA-seq reads using the phased heterozygous SNVs. The read counts of allele-specific RNA-seq reads were quantified using the software Kallisto (version 0.44.0) [17].

Identification of imprinted transcripts

For each tissue, the allele-specific RNA-seq reads of each PCG, lncRNA and TUCP were classified into two groups according to their respective parental origins, i.e., maternal or paternal allele. The R package DEseq2 [25] was employed to calculate the *P*-values and estimate significant differences in expression between parental alleles using the allele-specific RNA-seq read counts. The *P*-values were further corrected for multiple testing using the Benjamini-Hochberg procedure and the putative imprinted PCGs, lncRNAs and TUCPs were obtained under a strict corrected *P*-value cutoff of 0.05.

Classification of expression regulatory categories between breeds

We investigated the regulatory categories associated with expression differences between breeds for each tissue according to a previous study [12]. Specifically, we used the R package DEseq2 [25] to identify significant differential expression for each PCG, lncRNA and TUCP between breeds in both the F0 (**P**) and F1 (**F**) groups under a corrected *P*-value of 0.05. Fisher's exact test was used to evaluate breed-specific expression ratio differences between the **P** and **F** groups under a corrected *P*-value of 0.05 to detect *trans*-regulatory effects (**T**). The non-imprinted PCGs, lncRNAs and TUCPs with detectable expression were classified into seven categories according to the following criteria:

(1) *Cis*: significant differences detected in **P** and **F**, but not in **T**.

(2) *Trans*: significant differences detected in **P** and **T**, but not in **F**.

(3) *Cis+trans*: significant differences detected in **P**, **F** and **T**; the expression ratios between breeds were concordant between **P** and **F**.

(4) *Cis×trans*: significant differences detected in **P**, **F** and **T**; the expression ratios between breeds were opposite between **P** and **F**.

(5) Compensatory: significant differences detected in **F** and **T**, but not in **P**.

(6) Conserved: no significant differences detected in **P**, **F** and **T**.

(7) Ambiguous: All other patterns.

Single-nucleus RNA-seq and data processing

The nuclei of skeletal muscle obtained from purebred Berkshire and Tibetan pigs were purified and cut into pieces, and then digested in the GEXSCOPE Tissue Dissociation Solution (Singleron Biotechnologies) at 37℃ for 15 minutes. After digestion, a 40-micron sterile strainer was used to isolate the nucleus and remove other material. The resulting nuclei were centrifuged, and nucleus-containing pellets were resuspended in PBS (HyClone). The mixture was centrifuged and again resuspended in PBS. Single-nucleus RNA-seq libraries were constructed following the Singleron GEXSCOPETM protocol (Singleron Biotechnologies). The individual libraries were diluted to 4 ng/ml and pooled for sequencing on a HiSeq X ten platform (Illumina) with 150-bp paired-end reads. Low-quality reads and adaptor sequences were removed before generating gene expression matrices using the software CeleScope (https://github.com/singleron-RD/CeleScope/). The Seurat package (version 4.0.4) [26] available in R (version 4.0.5) was employed to filter cells. The independent samples were merged into a single object and clustered without supervision using the harmony package (version 0.1.0) [27]. Uniform Manifold Approximation and Projection (UMAP) [28] was applied to project all cells onto a two-dimensional map. The cell types were annotated based on the expression of canonical markers. The cell-type-specific highly expressed PCGs were identified using the 'FindAllMarkers' function available in the Seurat package with the following parameters: $\log_2$ fold-change > 0.25 and corrected P-value < 0.05.

Measurement of sequence conservation

PhastCons and PhyloP scores calculated by multiple alignment of 46 mammalian genomes were converted to the reference pig genome using the UCSC LiftOver tool (https://genome.ucsc.edu/cgi-bin/hgLiftOver). Average PhastCons and PhyloP scores were calculated for exonic regions of PCGs to compare sequence conservation across different regulatory categories. To calculate dN/dS ratios between pig versus human and pig versus mouse, we downloaded single-copy ortholog information from the Ensembl website

(version 107). The protein sequences of these orthologs were aligned between pig versus human and pig versus mouse using the software MUSCLE (version 3.8.31) [29]. The aligned files of protein sequences were then converted into nucleotide sequences, which were then used to calculate dN/dS ratios for each ortholog pair using the software KaKs_Calculator (version 3.0) [30].

Functional enrichment analysis

Functional enrichment analysis was performed using the Metascape tool [31]. Briefly, the PCGs in the pig genome were converted to human symbols according to pig-human orthologs and then used as input data for Metascape. Human (*Homo sapiens*) was chosen as the target species, and the enrichment analysis was performed against all PCGs in the reference pig genome with known human orthologs.

Results

Allele-specific expression profiling across pig tissues

To delineate the allele-specific expression profiling in pigs, we analyzed the rRNA-depleted RNA-seq data from a total of 24 F1 progenies generated by reciprocal crosses between two genetically and phenotypically distinct pig breeds: four families of Tibetan [♂] × Berkshire [♀], denoted as initial crosses; and four families of Berkshire [♂] × Tibetan [♀], denoted as reverse crosses (Fig. 1A and Supplementary Fig. S1). We then generated 145 RNA-seq libraries across seven tissues (brain [n = 18], heart [n = 23], kidney [n = 24], liver [n = 17], lung [n = 23], skeletal muscle [n = 18] and spleen [n = 22]) and downloaded 18 publicly available libraries for three tissues (brain [n = 6], liver [n = 6] and skeletal muscle [n = 6]) from a previous study [15]. This resulted in a total of 163 RNA-seq libraries with ~15.84 Gb high-quality data (Fig. 1A, Supplementary Fig. S1 and Data S1). To build a complete transcriptome for further analysis, we combined the annotations of PCGs and two transcript types (lncRNAs and TUCPs) from a previous study [16]. Considering random X-inactivation of expression in mammalian genomes [3], we analyzed 19,990 PCGs, 17,770 lncRNAs and 2205 TUCPs that encoded in autosomes.

The phasing of allele-specific RNA-seq reads in F1 progenies depends on the genotyping information of heterozygous SNVs [12, 14]. To that end, we sequenced whole-genome sequencing data for 22 individuals and downloaded the publicly available

sequencing data for another 18 individuals [15] (Berkshire pigs [*n* = 8], Tibetan pigs [*n* = 8] and F1 progenies [*n* = 24]) with a total coverage of ~126.25-fold (315.63 Gb) (Fig. 1A, Supplementary Figs. S1A and S2A). We identified ~11.02 million (M) heterozygous SNVs for each F1 progeny, of which ~95.35% were successfully phased using a trio-based strategy [22] (Supplementary Figs. S2B-D and S3A, B). This relatively high density of phased heterozygous SNVs (~4.64 SNVs per kilobase [kb]) allowed us to assign the expression of ~11,880 PCGs (72.61% of expressed [transcripts per million (TPM) > 0.5]), ~2982 lncRNAs (28.34% of expressed [TPM > 0.1]) and ~513 TUCPs (36.23% of expressed [TPM > 0.1]) to their parental alleles (see Materials and Methods; Supplementary Fig. S3C).

Taken together, we successfully constructed allele-specific expression profiles for three transcript types across seven tissues based on a reciprocal cross model between Berkshire and Tibetan pigs, considering the high genetic divergence between families, the similar genetic background within families, and differences between sexes (Supplementary Fig. S2E). This strategy allowed us to systematically explore regulatory differences responsible for expression changes between parental and breed-specific alleles.

The landscape of imprinted transcripts in the pig genome

Imprinting is a form of epigenetic regulation that causes the preferential expression of either maternally or paternally inherited alleles [32] (Fig. 1B). To construct a comprehensive category of imprinted transcripts in the pig genome, we examined significant expression differences between parental alleles for each of seven tissues under a strictly corrected *P*-value of 0.05 (see Materials and Methods). We identified 13-22 (~16.43) putative imprinted PCGs, 4-15 (~9.71) lncRNAs and 1-4 (~2.14) TUCPs in each tissue, with a significantly higher number of paternal versus maternal alleles when combining the three transcript types (~19.57 vs. ~8.57, *P* = 0.005, paired Student's *t*-test; Fig. 2A-C and Supplementary Figs. S4-6). This observation is in accordance with previous observations in human and mouse genomes [3, 5].

Among the union set of putative imprinted PCGs (*n* = 44), lncRNAs (*n* = 39) and TUCPs (*n* = 9) that were detected in at least one tissue, 69 (75.00%) were here first described in vertebrates, while the remaining 23 (25.00%) were reported accessed online

(Fig. 2D and Supplementary Data S2). Moreover, more than 50% (47 of 92) were located into 13 clusters (i.e., two imprinted transcripts located within 1 Mb genomic distance; Fig. 2E and Supplementary Data S2), which is also typical of known imprinted genes [3] and further demonstrate the reliability of imprinting identification.

Consistent with previous findings in hybrid mouse [3], we observed a tissue-dependent manner of imprinting, with 59.09% of PCGs and 74.36% of lncRNAs identified in a single tissue (Fig. 2F), suggesting imprinting impacts tissue-specific function. For example, *INPP5F* (inositol polyphosphate-5-phosphatase F) plays important role in regulating synaptic strengthening [33] and is expressed across all tissues, but only maintains paternal imprinting in the brain (Fig. 2E, G). Mutations in *INPP5F* were associated with synaptic depression and Alzheimer's Disease [34, 35].

Extensive *trans*-regulatory divergence between two pig breeds

The observed phenotypic differences can be explained by both *cis*- and *trans*-regulatory effects changing gene expression between breeds. To identify *cis*- and *trans*-regulatory effects, we performed rRNA-depleted RNA-seq for purebred Berkshire (n = 4) and Tibetan pigs (n = 4) across aforementioned seven tissues (n = 54), generating ~15.84 Gb of high-quality data per library (Fig. 1C and Supplementary Fig. S7). For each tissue, we examined the expression differences between breeds in both F0 (i.e., the expression of purebred Berkshire and Tibetan pigs) and F1 groups (i.e., the expression of Berkshire and Tibetan alleles in F1 progenies from reciprocal crosses) using 11,995-13,573 (~12,803) non-imprinted PCGs, 2050-5255 (~3535) non-imprinted lncRNAs and 383-809 (~577) non-imprinted TUCPs. These transcripts were expressed in both groups and included phased SNVs in F1 progenies to make it possible to distinguish breed-specific alleles (see Materials and Methods). We classified these transcripts into different categories based on the regulatory type of expression differences (see Materials and Methods; Figs. 1D, 3A, B and Supplementary Figs. S8-10). Consistent with previous findings in hybrid mouse and chicken [12-14], the majority showed evidence in conserved (60.17-74.85% of PCGs, 74.68-84.22% of lncRNAs and 67.63-77.5% of TUCPs for each tissue) or ambiguous (18.21-24.96% of PCGs, 12.96-20.58% of lncRNAs and 18.17-25.19% of TUCPs for each tissue) regulatory patterns (Fig. 3A, B and Supplementary Figs. S8-10).

Notably, we observed a significantly higher number of *trans*-regulatory PCGs (ranging from 230 to 1594, ~831) compared to *cis*-regulatory PCGs (ranging from 116 to 235, ~177) (P = 0.008, paired Student's t-test; Fig. 3C), suggesting the dominant contribution of former to PCG expression changes between breeds. However, an opposite trend was found in lncRNAs (~88.86 of *cis* vs. ~30.42 of *trans*, P = 0.008, paired Student's t-test; Fig. 3D) and TUCPs (~21.29 of *cis* vs. 9.57 of *trans*, P = 0.03, paired Student's t-test; Fig. 3E), indicating the mechanisms responsible for expression changes differ across transcript types.

***Trans*-regulatory effects underline adaptive phenotypic divergence between two pig breeds**

The higher number of *trans*-regulatory PCGs (Fig. 3C) suggests they are major drivers shaping the phenotypic differences observed between pig breeds, which might be subject to strong selective pressions. Supporting this hypothesis, we found that the *trans*-regulatory PCGs with higher expression in Tibetan pigs were mainly enriched in 'protein folding' (heart, kidney, liver, lung, skeletal muscle and spleen; 12-42 PCGs), 'protein processing in endoplasmic reticulum' (heart, liver, lung and skeletal muscle; 17-32 PCGs), 'mitochondrion organization' and 'mitochondrial translation' (heart, kidney and liver; 14-29 PCGs) (Fig. 3F and Supplementary Fig. S11). These pathways likely reflect the general ability for special protein misfolding and energy utilization in Tibetan pigs exposed to harsher environmental conditions [36, 37]. Specifically, the spleen, which is the major organ associated with erythrocyte function and immune system [38], might be responsible for the impressive adaptive ability of Tibetan pigs to overcome hypoxia and ultraviolet radiation in highland environment, supported by the pathways of 'heme metabolic process' (12 PCGs), 'DNA-templated DNA replication' (18 PCGs), 'erythrocyte differentiation' (9 PCGs), 'cellular response to radiation' (6 PCGs), 'gas transport' (4 PCGs) and 'transferrin transport' (3 PCGs) (Fig. 3F).

Compared to Tibetan pigs, *trans*-regulatory PCGs with higher expression in Berkshire pigs were mainly associated with pathways related to artificial selection, including 'lipid catabolic process' (liver; 26 PCGs), 'insulin receptor signaling pathway' (heart; 9 PCGs), 'response to hormone' (brain, kidney and lung; 19, 31 and 18 PCGs) (Supplementary Fig. S11), which reflect higher metabolic efficiency and growth rate in this breed. Notably,

enriched pathways in the skeletal muscle (i.e., the tissue associated with major commercial traits of pork yield and quality) provide strong evidence for long-term artificial selection of muscle growth and hypertrophy, as well as higher proportion of lean meat production in Berkshire pigs, supported by ‘autophagy of mitochondrion’ (18 PCGs), ‘response to insulin’ (28 PCGs), ‘response to nutrient levels’ (26 PCGs), ‘mTOR signaling pathway’ [39] (22 PCGs), ‘AMPK signaling pathway’ [40] (20 PCGs) (Fig. 3G). We note that industrially farmed Berkshire pigs were constantly exposed to environmental xenobiotics (e.g., chemicals and drugs) through food, water and air, and thus obtained a better innate immunity [37], as shown by the pathways enriched in spleen of ‘response to interferon-alpha’ (6 PCGs), ‘cellular response to cytokine stimulus’ (22 PCGs), ‘regulation of response to biotic stimulus’ (14 PCGs), ‘regulation of type I interferon-mediated signaling pathway’ (5 PCGs) and ‘response to dexamethasone’ (5 PCGs) (Supplementary Fig. S11).

In addition, we also found that the *trans*-regulatory PCGs showed significantly higher expression levels in the testable tissues than others (Fig. 3H, I and Supplementary Fig. S12), indicating their important role in tissue function.

***Trans*-regulatory effects in skeletal muscle were associated with different cell types**

Mammalian skeletal muscles are heterogeneous, composed of different myofibers and other cell types [41]. To query whether certain cell types contribute more to *trans*-regulatory effects in skeletal muscle, we performed single-nucleus RNA-seq from this tissue on purebred Berkshire and Tibetan pigs, and harvested the transcriptome of 24,683 cells (with 12,074 from Berkshire and 12,609 from Tibetan pigs; Fig. 4A). These cells were then subdivided into 12 distinct cell types, which exhibited different composition between breeds, especially for the three myofibers (types Ⅰ, ⅡA and ⅡB; Fig. 4A, B and Supplementary Fig. S13).

We next sought to investigate whether distinct cell type composition was associated with *trans*-regulatory differences in skeletal muscle between breeds. We detected 67-267 (~166.33) and 25-305 (~143.42) cell-type-specific highly expressed PCGs in Berkshire and Tibetan pigs, respectively (see Materials and Methods; Fig. 4C, D). Notably, we found the *trans*-regulatory PCGs of skeletal muscle with higher expression in Tibetan pigs were specifically enriched in cell-type-specific highly expressed PCGs of type Ⅰ myofibers and

muscle satellite (Fig. 4C, D). These observations suggest a major contribution of the two cell types to the enhanced *trans*-regulatory effects in skeletal muscle of Tibetan pigs. Type Ⅰ (slow-twitch or oxidative) myofibers can accelerate lipid accumulation in skeletal muscle [42, 43], providing enhanced athletic performance and a substantial energy source to survive in high-altitude environments characteristic of Tibet. Muscle satellite cells are resident stem cells from skeletal muscle that are activated by hypoxia niches to regulate proliferation and differentiation of myofibers during muscle regeneration [44, 45], and beneficial for the survival of Tibetan pigs. In contrast, the higher expression of *trans*-regulatory PCGs of skeletal muscle in Berkshire pigs was specifically enriched in cell-type-specific highly expressed PCGs of type ⅡA myofibers (fast-twitch or oxidative/glycolytic; Fig. 4C, D). These myofibers can increase the ability to metabolize carbohydrates for rapid growth while reducing pH, intramuscular fat content and water holding capacity that impairs pork quality [42, 43].

Taken together, these results demonstrate that *trans*-regulatory effects in skeletal muscle result from different cell type composition and reveal the potential contribution of different cell types to expression differences between breeds.

***Cis*-regulatory effects exhibited greater sequence divergence**

While *trans*-regulatory effects are caused by mutations in diffusible components (e.g., transcription factors), *cis*-regulatory effects mainly result from genetic variants at *cis*-regulatory elements (typically, promoters and enhancers) [7, 12]. To investigate the association between genetic variants and *cis*-regulatory effects, we evaluated the inter-breed sequence divergence in promoter sequences (i.e., 2kb upstream regions of transcription start sites [TSS] of PCGs, lncRNAs and TUCPs) using the heterozygous SNVs and InDels detected in each F1 progeny. As expected, we found a significantly higher occurrence of genetic variants in the promoter regions of *cis*-regulatory PCGs compared to any other regulatory category considered (e.g., median of 9.25 SNVs in *cis* vs. 6.83 in *trans*, $P < 0.05$, Wilcoxon rank-sum test; Fig. 5A). A similar but weaker distribution was also found in lncRNAs and TUCPs (Supplementary Fig. S14). Considering the lower conservation scores (e.g., median PhastCons of 0.42 for exonic regions in *cis* vs. 0.5 in *trans*, $P < 0.001$, Wilcoxon rank-sum test; Supplementary Fig. S15A, B) and larger dN/dS

ratios between pig orthologs with human and mouse (e.g., median of 0.14 in *cis* vs. 0.11 in *trans*, $P < 0.05$, Wilcoxon rank-sum test; Supplementary Fig. S15C, D), our data confirms previous findings in hybrid mouse [13] and chicken [12] showing *cis*-regulated genes are less conserved.

Since the three transcript types showed distinct conservation levels (Fig. 5B, C), we next compared the degree of *cis*-regulatory effects on expression differences (i.e., the absolute $\log_2$ fold-change of expression between breeds) between transcript types. Interestingly, lncRNAs and TUCPs showed much lower sequence conservation (e.g., for PhastCons, median of 0.05 and 0.07 in lncRNAs and TUCPs vs. 0.48 in PCGs, $P < 2.2\times10^{-16}$, Wilcoxon rank-sum test; Fig. 5B, C) and exhibited a significantly greater expression divergence between breeds compared to PCGs (e.g., median of 1.51 in combined of lncRNAs and TUCPs vs. 1.17 in PCGs for F0 group, $P < 0.03$, Wilcoxon rank-sum test; Fig. 5D, E). This was also observed in *trans*-regulatory effects (Fig. 5F) despite small lncRNAs and TUCPs numbers.

Taken together, these results confirm the potential contribution of sequence variants in *cis*-regulatory elements to changes in expression between pig breeds.

The majority of *cis*- and *trans*-regulatory effects are tissue-specific

Recent studies suggested a tissue-dependent manner of regulatory effects on gene expression [46]. Similary, we found that the majority of *cis*- (75.38% of PCGs, 75.85% of lncRNAs and 76.42% of TUCPs) and *trans*-regulatory effects (65.55% of PCGs, 89.25% of lncRNAs and 89.47% of TUCPs) were detected in a single tissue (Fig. 6A), highlighting a tissue-dependent response to the same regulatory variants. Interestingly, transcripts with the same regulatory pattern in two or more tissues exhibited similar levels of expression differences between breeds (i.e., the $\log_2$ fold-change expression of Berkshire to Tibetan), as shown by the high correlation coefficient of *trans*-regulatory effects in the F0 group (Pearson's $r = 0.9$, $n = 3382$ tissue pairs, $P < 2.2\times10^{-16}$; Fig. 6B), as well as *cis*-regulatory in both the F0 (Pearson's $r = 0.91$, $n = 648$ tissue pairs, $P < 2.2\times10^{-16}$; Fig. 6C) and F1 (Pearson's $r = 0.91$, $n = 648$ tissue pairs, $P < 2.2\times10^{-16}$; Fig. 6D) groups. This is similar to the correlation observed in *cis*-regulatory between F0 and F1 groups within the same tissue (Pearson's $r = 0.91$, $n = 2007$ F0-F1 pairs, $P < 2.2\times10^{-16}$; Fig. 6E), indicating that the same

sequence variants in pleiotropic regulatory elements may cause similar differential expression between breeds among different tissues [47].

Discussion

A reciprocal cross strategy has been widely used to analyze allele-specific expression patterns [3, 6, 12, 13]. Using Berkshire and Tibetan pigs, we generate a reciprocal cross model to investigate the allele-specific regulatory effects on pig transcriptome. We detected a relatively high ratio of heterozygous SNVs in F1 progenies that reached ~4.64 heterozygous SNVs per kb. This is still lower than hybrid mouse (~7.7 per kb), but comparable to hybrid fruit flies (~5 per kb) and higher than humans (~1 per kb) [48, 49]. In general, this high heterozygous ratio demonstrates Berkshire and Tibetan pigs are ideal to build reciprocal cross models to study allele-specific expression differences between breeds. Berkshire pigs represent the typical European commercial pig breed that underwent strong artificial selection for greater pork yield, larger body size and higher growth rate than Tibetan pigs.

Imprinted genes play important roles in viability, development, growth and various other physiological functions [5]. Previously, approximately 200 imprinted PCGs and lncRNAs were identified in the human and mouse genomes [5]. Despite several studies were conducted to identify imprinted genes in the pig genome [50-52], until now only 36 had been reported (https://geneimprint.com/site/home). In addition, no imprinted non-coding transcripts (except H19) had been detected due to the poor annotation of the reference pig genome. In this study, we provided a comprehensive annotation for putative imprinted PCGs, lncRNAs and TUCPs, which greatly expands current understanding of genomic imprinting in this species.

The occurrence of *trans*-regulatory variants influences the expression of a large number of genes [7]. To maintain stable gene expression, other regulatory components may act in a compensatory way to eliminate the most deleterious *trans*-acting variants [7, 14]. Hence, *trans*-regulatory expression divergence between breeds may have evolved under strong selective pressures, especially for agricultural domesticated animals [12, 13]. We observed a significantly higher proportion of *trans*-regulatory effects versus *cis* in PCGs (Fig. 3C), suggesting that artificial selection primarily acted on *trans*-regulatory mutations

and associated functional pathways.

Notably, we found an extremely low proportion of PCGs regulated by both *cis* and *trans*-elements (~0.13% of *cis*+*trans* and ~0.1% of *cis*×*trans* in each tissue; Fig. 3A, B and Supplementary Fig. S8). The limited number of complex interactions between *cis* and *trans* demonstrates that artificial selection accelerated the expression divergence between breeds mainly through *trans*-regulatory variants. In contrast, *cis*-regulatory mutations with opposite effects likely served as complementary factors maintaining stable gene expression had not accumulated substantially during the short divergence period since the two breeds were established.

Additional Files

Supplementary Fig. S1. Summary of rRNA-depleted RNA-seq data of F1 progenies.

Supplementary Fig. S2. Whole genome sequencing and SNV calling.

Supplementary Fig. S3. Phasing of heterozygous SNVs and assignment of expression.

Supplementary Fig. S4. Identification of imprinted PCGs.

Supplementary Fig. S5. Identification of imprinted lncRNAs.

Supplementary Fig. S6. Identification of imprinted TUCPs.

Supplementary Fig. S7. Summary of rRNA-depleted RNA-seq data of purebred Berkshire and Tibetan pigs.

Supplementary Fig. S8. Classification of *cis*- and *trans*-regulatory effects of PCGs.

Supplementary Fig. S9. Classification of *cis*- and *trans*-regulatory effects of lncRNAs.

Supplementary Fig. S10. Classification of *cis*- and *trans*-regulatory effects of TUCPs.

Supplementary Fig. S11. GO and KEGG enrichment for trans-regulatory PCGs with higher expression in Berkshire (blue) and Tibetan (pink) for each tissue.

Supplementary Fig. S12. The TPM ranks of each tissue for trans-regulatory PCGs.

Supplementary Fig. S13. Classification of different cell types.

Supplementary Fig. S14. Distribution of SNV number, InDel number and InDel length of promoters in the combined of lncRNAs and TUCPs.

Supplementary Fig. S15. The conservation comparison between different regulatory categories for PCGs.

Supplementary Data S1. Sample description.

Supplementary Data S2. Putative imprinted transcripts.

Data accessibility

The whole genome sequencing data (n = 22) and rRNA-depleted RNA-seq data (n = 199) are available at the Genome Sequence Archive (GSA, https://bigd.big.ac.cn/gsa/). We provided reviewer access as follows: to review the datasets deposited at GSA (CRA009370), https://ngdc.cncb.ac.cn/gsa/s/nQfZe5UE.

Abbreviations.

ASE: allele-specific expression; PCG: protein-coding genes; lncRNA: long non-coding RNA; TUCP: transcripts of unknown coding potential; SNV: single nucleotide variant; InDel: insertion and deletion; Gb: gigabase; kb: kilobase; BWA: Burrows-Wheeler Aligner; GATK: Genome Analysis Toolkit; UMAP: Uniform Manifold Approximation and Projection; TPM: transcripts per million.

Competing Interests

The authors declare they have no competing interests.

Funding

This work was supported by the National Key R & D Program of China (2022YFF1000100 to J.L., and 2021YFD1301102 to L.C.), the Tackling Project for Agricultural Key Core Technologies of China (NK2022110602 to L.J.), the National Natural Science Foundation of China (32102507 and U22A20507 to J.L., 32072687 to L.G., and 32225046 to M.L.), the Sichuan Science and Technology Program (2022NSFSC1618 to J.L., 2021ZDZX0008 to L.J. and K.L.) and the Major Science and Technology Projects of Tibet Autonomous Region (XZ202101ZD0005N to M.L.).

Authors' contributions

Y.L.: conceptualization, data curation, formal analysis, investigation, methodology, validation, writing—original draft; J.L.: conceptualization, funding acquisition, investigation, supervision, writing—review and editing; L.C.: funding acquisition, resources, supervision; J.B.: investigation; J.Z.: investigation; Y.W.: investigation; P.L.: investigation; K.L.: funding acquisition, investigation; L.G.: funding acquisition, resources; L.J.: funding acquisition,

supervision; Y.G.: resources; M.L.: conceptualization, funding acquisition, project administration, supervision, writing—review and editing.

Acknowledgements

We thank the High-Performance Computing Platform of Sichuan Agricultural University for providing computing resources and support that have contributed to this research.

Figure legends

Figure 1. Study design. (**A**) Schematic diagram of reciprocal crosses between genetically distinct Berkshire and Tibetan pig breeds. We collected a total of 163 rRNA-depleted RNA-seq libraries across seven tissues (brain, heart, kidney, liver, lung, skeletal muscle and spleen) from 24 F1 progenies, including 145 libraries newly generated and 18 libraries downloaded. Whole-genome sequencing data (n = 40) were collected, including 22 newly generated and 18 downloaded. (**B**) Schematic representation of imprinting. (**C**) A total of 54 rRNA-depleted RNA-seq libraries across seven tissues from purebred Berkshire (n = 4) and Tibetan pigs (n = 4) were generated. (**D**) The schematic diagram illustrates example expression patterns for different regulatory categories between breeds. *Cis*: concordant significant expression divergence between breeds was observed in the F0 and F1 groups. *Trans*: significant expression divergence between breeds was observed in the F0 but not the F1 group. See detailed information in Material and methods.

Figure 2. The landscape of imprinted transcripts across tissues. (**A**) Identification of imprinted PCGs in the brain. See the results of others tissues in Supplementary Figs. S4-6. (**B**) The number of putative imprinted PCGs, lncRNAs and TUCPs (bars, left) for each tissue. The pie chart on the right shows the number and proportion of maternal and paternal imprinted transcripts. (**C**) The bar plots show the significantly higher number of paternal imprinted transcripts (blue, right) compared to maternal (red, left). P-values were calculated using a paired Student's t-test. (**D**) The comparison of the proportion reported in the genomic imprinting website (https://geneimprint.com/site/home) for putative imprinted transcripts (left) and others (right). The P-value was calculated using a χ-test. (**E**) The

heatmap shows the allele-specific expression patterns for the union set of putative imprinted PCGs (n = 44), lncRNAs (n = 39) and TUCPs (n = 9) that were detected in at least one tissue. The cluster (i.e., two adjacent imprinted transcripts were located with 1Mb genomic distance, purple lines) and chromosome information are shown on the right. Manhattan plot showing the allelic expression ratio (i.e., the ratio of preferential expressed allele, the maximal values across seven tissues were used to display). The symbols of putative imprinted transcripts that were reported in the genomic imprinting website are shown on the left. The allele-specific expression ratio is shown for each putative imprinted transcript across seven tissues (0 means 100% expression from the paternal allele while 1 means 100% expression from the maternal allele). (**F**) The pie charts show the number of tissues that maintained the imprinting status in PCGs (left) and lncRNAs (right). TUCPs were ignored due to smaller number. (**G**) The expression levels for maternal and paternal alleles expressed across seven tissues are shown for the brain-specific paternally imprinted PCG of *INPP5F*.

Figure 3. Classification of expression regulatory categories between pig breeds. (**A** and **B**) Classification of *cis*- and *trans*-regulatory effects of PCGs in skeletal muscle (**A**) and brain (**B**). See the results obtained in other tissues in Supplementary Figs. S8-10. (**C**-**E**) The number of *cis*- (bars, red) and *trans*-regulatory (bars, yellow) effects in PCGs (**C**), lncRNAs (**D**) and TUCPs (**E**). (**F** and **G**) The enriched GO and KEGG pathways for *trans*-regulatory PCGs with higher expression in the spleen of Tibetan (**F**) and the skeletal muscle of Berkshire pigs (**G**). (**H** and **I**) The TPM ranks of each tissue for *trans*-regulatory PCGs in the skeletal muscle (**H**) and the brain (**I**). The ranks represent the normalized expression levels in each tissue, from lower to higher. The P-values were calculated using the Wilcoxon rank-sum test.

Figure 4. The correlation between *trans*-regulatory effects and cell type composition in skeletal muscle. (**A**) Cell type identification using UMAP clustering (left) and the projected coordination in Berkshire (right, top) and Tibetan pigs (right, bottom). (**B**) The proportion of cells for each cell type in Berkshire (blue, bars) and Tibetan pigs (pink, bars). (**C** and **D**)

The relationship between cell-type-specific highly expressed PCGs and *trans*-regulatory effects in skeletal muscle. An enrichment score was calculated to evaluate whether the *trans*-regulatory PCGs are enriched in cell-type-specific highly expressed PCGs in Berkshire (**C**) or Tibetan pigs (**D**).

Figure 5. Cis-regulatory effects showing greater sequence divergence. (**A**) Boxplots showing the distribution of SNV number (top), InDel number (middle) and InDel length (bottom) for each promoter of PCG among the different regulatory categories of cis (red), trans (yellow), conserved (grey) and others (white). Median of 9.25 SNVs in *cis* vs. 6.83 in *trans*, 5.92 in conserved and6.58 in others, $P < 0.05$ for all tissues; median of 1.63 InDels in *cis* vs. 1.29 in *trans*, 1.08 in conserved and 1.25 in others, $P < 0.05$ except for liver and spleen; median of 4.17 bp InDles in *cis* vs. 3.04 in *trans*, 2.58 in conserved and 2.96 in others, $P < 0.05$ except for kidney and spleen. (**B** and **C**) Violin plots showing the conservation level of PhastCons (**B**) and PhyloP (**C**) for the exonic regions of PCGs, lncRNAs and TUCPs. PhastCons: median of 0.05 and 0.07 in lncRNAs and TUCPs vs. 0.48 in PCGs, $P < 2.2\times10^{-16}$; PhyloP: median of 0.05 and 0.02 in lncRNAs and TUCPs vs. 0.67 in PCGs, $P < 2.2\times10^{-16}$. (**D**-**F**) Violin plots showing the degree of expression divergence between breeds (i.e., the absolute log2 fold-change of expression between breeds) between PCGs and the combined of lncRNAs and TUCPs for *cis*-regulatory effects in F0 (**D**) and F1 groups (**E**), as well as for trans-regulatory effects in F0 (**F**). Median of 1.51 in combined of lncRNAs and TUCPs vs. 1.17 in PCGs for F0 group, $P < 0.03$ for all tissues; median of 1.14 in combined of lncRNAs and TUCPs vs. 0.83 in PCGs for F1 group, $P < 0.009$ except for spleen; median of 1.79 in combined of lncRNAs and TUCPs vs. 1.01 in PCGs for F0 group, $P < 1.21\times10^{-5}$ except for brain.

The number of testable transcripts for each category were shown (top) and the *P*-values were calculated by Wilcoxon rank-sum test (n.s. means: $P \geq 0.05$; *means: $0.01 < P < 0.05$; ** means: $0.001 < P < 0.01$; *** means: $P < 0.001$).

Figure 6. The tissue-specific patterns of *cis*- and *trans*-regulatory effects. (**A**) Pie charts showing the number of tissues with *cis*- or *trans*-regulatory effects for PCGs, lncRNAs and

TUCPs. (**B**-**D**) Scatter heatmaps showing the Pearson's correlation of $\log_2$ expression fold-change (Berkshire / Tibetan) for the same regulatory status between tissue pairs, for *trans*-regulatory effects in the F0 group (**B**), and *cis*-regulatory effects in the F0 (**C**) and F1 (**D**) groups. (**E**) Scatter heatmap showing the Pearson's correlation of $\log_2$ expression fold-change (Berkshire / Tibetan) between the F0 and F1 groups within the same tissue in *cis*-regulatory effects.

References

1. Andergassen D, Dotter CP, Kulinski TM, Guenzl PM, Bammer PC, Barlow DP, et al. Allelome.PRO, a pipeline to define allele-specific genomic features from high-throughput sequencing data. Nucleic acids research. 2015;43 21:e146. doi:10.1093/nar/gkv727.
2. St Pierre CL, Macias-Velasco JF, Wayhart JP, Yin L, Semenkovichf CF and Lawson HA. Genetic, epigenetic, and environmental mechanisms govern allele-specific gene expression. Genome Res. 2022;32 6:1042-57. doi:10.1101/gr.276193.121.
3. Andergassen D, Dotter CP, Wenzel D, Sigl V, Bammer PC, Muckenhuber M, et al. Mapping the mouse Allelome reveals tissue-specific regulation of allelic expression. Elife. 2017;6 doi:10.7554/eLife.25125.
4. Santini L, Halbritter F, Titz-Teixeira F, Suzuki T, Asami M, Ma X, et al. Genomic imprinting in mouse blastocysts is predominantly associated with H3K27me3. Nat Commun. 2021;12 1:3804. doi:10.1038/s41467-021-23510-4.
5. Tucci V, Isles AR, Kelsey G, Ferguson-Smith AC and Erice Imprinting G. Genomic Imprinting and Physiological Processes in Mammals. Cell. 2019;176 5:952-+. doi:10.1016/j.cell.2019.01.043.
6. Metzger BPH, Duveau F, Yuan DC, Tryban S, Yang B and Wittkopp PJ. Contrasting Frequencies and Effects of cis- and trans-Regulatory Mutations Affecting Gene Expression. Molecular Biology and Evolution. 2016;33 5:1131-46. doi:10.1093/molbev/msw011.
7. Signor SA and Nuzhdin SV. The Evolution of Gene Expression in cis and trans. Trends in Genetics. 2018;34 7:532-44. doi:10.1016/j.tig.2018.03.007.
8. Crowley JJ, Zhabotynsky V, Sun W, Huang S, Pakatci IK, Kim Y, et al. Analyses of allele-specific gene expression in highly divergent mouse crosses identifies pervasive allelic imbalance. Nat Genet. 2015;47 4:353-60. doi:10.1038/ng.3222.
9. Schaefke B, Emerson JJ, Wang TY, Lu MYJ, Hsieh LC and Li WH. Inheritance of Gene Expression Level and Selective Constraints on Trans- and Cis-Regulatory Changes in Yeast. Molecular Biology and Evolution. 2013;30 9:2121-33. doi:10.1093/molbev/mst114.
10. Fear JM, Leon-Novelo LG, Morse AM, Gerken AR, Van Lehmann K, Tower J, et al. Buffering of Genetic Regulatory Networks in Drosophila melanogaster. Genetics. 2016;203 3:1177-+. doi:10.1534/genetics.116.188797.
11. McManus CJ, Coolon JD, Duff MO, Eipper-Mains J, Graveley BR and Wittkopp PJ.

Regulatory divergence in Drosophila revealed by mRNA-seq (vol 20, pg 816, 2010). Genome Research. 2014;24 6:1051-.

12. Wang Q, Jia YX, Wang Y, Jiang ZH, Zhou X, Zhang ZB, et al. Evolution of cis- and trans-regulatory divergence in the chicken genome between two contrasting breeds analyzed using three tissue types at one-day-old. Bmc Genomics. 2019;20 1 doi:10.1186/s12864-019-6342-5.
13. Goncalves A, Leigh-Brown S, Thybert D, Stefflova K, Turro E, Flicek P, et al. Extensive compensatory cis-trans regulation in the evolution of mouse gene expression. Genome Research. 2012;22 12:2376-84. doi:10.1101/gr.142281.112.
14. Mack KL, Campbell P and Nachman MW. Gene regulation and speciation in house mice. Genome Research. 2016;26 4:451-61. doi:10.1101/gr.195743.115.
15. Lin Y, Li J, Gu YR, Jin L, Bai JY, Zhang JM, et al. Allele-specific Effects of Three-dimensional Genome Architecture in Hybrid Pigs. 2022; Preprint available at Research Square. (doi:10.21203/rs.3.rs-2392032/v2).
16. Jin L, Tang QZ, Hu SL, Chen ZX, Zhou XM, Zeng B, et al. A pig BodyMap transcriptome reveals diverse tissue physiologies and evolutionary dynamics of transcription. Nat Commun. 2021;12 1 doi:10.1038/s41467-021-23560-8.
17. Bray NL, Pimentel H, Melsted P and Pachter L. Near-optimal probabilistic RNA-seq quantification. Nature biotechnology. 2016;34 5:525-7. doi:10.1038/nbt.3519.
18. Li H and Durbin R. Fast and accurate short read alignment with Burrows-Wheeler transform. Bioinformatics. 2009;25 14:1754-60. doi:10.1093/bioinformatics/btp324.
19. McKenna A, Hanna M, Banks E, Sivachenko A, Cibulskis K, Kernytsky A, et al. The Genome Analysis Toolkit: a MapReduce framework for analyzing next-generation DNA sequencing data. Genome Res. 2010;20 9:1297-303. doi:10.1101/gr.107524.110.
20. Yang J, Lee SH, Goddard ME and Visscher PM. GCTA: a tool for genome-wide complex trait analysis. American journal of human genetics. 2011;88 1:76-82. doi:10.1016/j.ajhg.2010.11.011.
21. Pritchard JK, Stephens M and Donnelly P. Inference of population structure using multilocus genotype data. Genetics. 2000;155 2:945-59. doi:10.1093/genetics/155.2.945.
22. Choi Y, Chan AP, Kirkness E, Telenti A and Schork NJ. Comparison of phasing strategies for whole human genomes. PLoS Genet. 2018;14 4:e1007308. doi:10.1371/journal.pgen.1007308.
23. Dobin A, Davis CA, Schlesinger F, Drenkow J, Zaleski C, Jha S, et al. STAR: ultrafast universal RNA-seq aligner. Bioinformatics. 2013;29 1:15-21. doi:10.1093/bioinformatics/bts635.
24. Krueger F and Andrews SR. SNPsplit: Allele-specific splitting of alignments between genomes with known SNP genotypes. F1000Research. 2016;5:1479. doi:10.12688/f1000research.9037.2.
25. Love MI, Huber W and Anders S. Moderated estimation of fold change and dispersion for RNA-seq data with DESeq2. Genome Biology. 2014;15 12 doi:10.1186/s13059-014-0550-8.
26. Hao Y, Hao S, Andersen-Nissen E, Mauck WM, 3rd, Zheng S, Butler A, et al. Integrated analysis of multimodal single-cell data. Cell. 2021;184 13:3573-87.e29.

doi:10.1016/j.cell.2021.04.048.

27. Korsunsky I, Millard N, Fan J, Slowikowski K, Zhang F, Wei K, et al. Fast, sensitive and accurate integration of single-cell data with Harmony. Nature Methods. 2019;16 12:1289-+. doi:10.1038/s41592-019-0619-0.

28. Becht E, McInnes L, Healy J, Dutertre CA, Kwok IWH, Ng LG, et al. Dimensionality reduction for visualizing single-cell data using UMAP. Nature biotechnology. 2019;37 1:38-+. doi:10.1038/nbt.4314.

29. Edgar RC. MUSCLE: multiple sequence alignment with high accuracy and high throughput. Nucleic acids research. 2004;32 5:1792-7. doi:10.1093/nar/gkh340.

30. Zhang Z. KaKs_Calculator 3.0: Calculating Selective Pressure on Coding and Non-coding Sequences. Genomics, proteomics & bioinformatics. 2022; doi:10.1016/j.gpb.2021.12.002.

31. Zhou Y, Zhou B, Pache L, Chang M, Khodabakhshi AH, Tanaseichuk O, et al. Metascape provides a biologist-oriented resource for the analysis of systems-level datasets. Nat Commun. 2019;10 1:1523. doi:10.1038/s41467-019-09234-6.

32. Weinberg-Shukron A, Ben-Yair R, Takahashi N, Dunjic M, Shtrikman A, Edwards CA, et al. Balanced gene dosage control rather than parental origin underpins genomic imprinting. Nature Communications. 2022;13 1:12. doi:10.1038/s41467-022-32144-z.

33. Nakatsu F, Messa M, Nandez R, Czapla H, Zou YX, Strittmatter SM, et al. Sac2/INPP5F is an inositol 4-phosphatase that functions in the endocytic pathway. Journal of Cell Biology. 2015;209 1:85-95. doi:10.1083/jcb.201409064.

34. Cao MA, Park D, Wu YM and De Camilli P. Absence of Sac2/INPP5F enhances the phenotype of a Parkinson's disease mutation of synaptojanin 1. Proceedings of the National Academy of Sciences of the United States of America. 2020;117 22:12428-34. doi:10.1073/pnas.2004335117.

35. Xue F, Gao LY, Chen TT, Chen HY, Zhang HH, Wang T, et al. Parkinson's Disease rs117896735 Variant Regulates INPP5F Expression in Brain Tissues and Increases Risk of Alzheimer's Disease. Journal of Alzheimers Disease. 2022;89 1:67-77. doi:10.3233/jad-220086.

36. Bartoszewska S and Collawn JF. Unfolded protein response (UPR) integrated signaling networks determine cell fate during hypoxia. Cellular & Molecular Biology Letters. 2020;25 1 doi:10.1186/s11658-020-00212-1.

37. Li MZ, Tian SL, Jin L, Zhou GY, Li Y, Zhang Y, et al. Genomic analyses identify distinct patterns of selection in domesticated pigs and Tibetan wild boars. Nature Genetics. 2013;45 12:1431-U180. doi:10.1038/ng.2811.

38. Bronte V and Pittet MJ. The Spleen in Local and Systemic Regulation of Immunity. Immunity. 2013;39 5:806-18. doi:10.1016/j.immuni.2013.10.010.

39. Yoon MS. mTOR as a Key Regulator in Maintaining Skeletal Muscle Mass. Frontiers in Physiology. 2017;8 doi:10.3389/fphys.2017.00788.

40. Yao CG, Pang DX, Lu C, Xu AS, Huang PX, Ouyang HS, et al. Data Mining and Validation of AMPK Pathway as a Novel Candidate Role Affecting Intramuscular Fat Content in Pigs. Animals. 2019;9 4 doi:10.3390/ani9040137.

41. Petrany MJ, Swoboda CO, Sun CY, Chetal K, Chen XT, Weirauch MT, et al. Single-nucleus RNA-seq identifies transcriptional heterogeneity in multinucleated skeletal

myofibers. Nature Communications. 2020;11 1 doi:10.1038/s41467-020-20063-w.

42. Ryu YC and Kim BC. The relationship between muscle fiber characteristics, postmortem metabolic rate, and meat quality of pig longissimus dorsi muscle. Meat science. 2005;71 2:351-7. doi:10.1016/j.meatsci.2005.04.015.

43. Zhao L, Huang Y and Du M. Farm animals for studying muscle development and metabolism: dual purposes for animal production and human health. Animal Frontiers. 2019;9 3:21-7. doi:10.1093/af/vfz015.

44. Pircher T, Wackerhage H, Aszodi A, Kammerlander C, Bocker W and Saller MM. Hypoxic Signaling in Skeletal Muscle Maintenance and Regeneration: A Systematic Review. Frontiers in Physiology. 2021;12 doi:10.3389/fphys.2021.684899.

45. Liu WY, Wen YF, Bi PP, Lai XS, Liu XS, Liu XQ, et al. Hypoxia promotes satellite cell self-renewal and enhances the efficiency of myoblast transplantation. Development. 2012;139 16:2857-65. doi:10.1242/dev.079665.

46. Mugal CF, Wang M, Backstrom N, Wheatcroft D, Alund M, Semon M, et al. Tissue-specific patterns of regulatory changes underlying gene expression differences among Ficedula flycatchers and their naturally occurring F 1 hybrids. Genome Research. 2020;30 12 doi:10.1101/gr.254508.119.

47. Shen SQ, Turro E and Corbo JC. Hybrid mice reveal parent-of-origin and Cis- and trans-regulatory effects in the retina. PloS one. 2014;9 10:e109382. doi:10.1371/journal.pone.0109382.

48. de Wit E. Capturing heterogeneity: single-cell structures of the 3D genome. Nature Structural & Molecular Biology. 2017;24 5:437-8. doi:10.1038/nsmb.3404.

49. Abed JA, Erceg J, Goloborodko A, Nguyen SC, McCole RB, Saylor W, et al. Highly structured homolog pairing reflects functional organization of the Drosophila genome. Nature Communications. 2019;10 doi:10.1038/s41467-019-12208-3.

50. Hou X, Wang Z, Shi L, Wang L, Zhao F, Liu X, et al. Identification of imprinted genes in the skeletal muscle of newborn piglets by high-throughput sequencing. Animal genetics. 2022;53 4:479-86. doi:10.1111/age.13212.

51. Yu D, Wang J, Zou H, Feng T, Chen L, Li J, et al. Silencing of retrotransposon-derived imprinted gene RTL1 is the main cause for postimplantational failures in mammalian cloning. Proc Natl Acad Sci U S A. 2018;115 47:E11071-e80. doi:10.1073/pnas.1814514115.

52. Bischoff SR, Tsai S, Hardison N, Motsinger-Reif AA, Freking BA, Nonneman D, et al. Characterization of conserved and nonconserved imprinted genes in swine. Biology of reproduction. 2009;81 5:906-20. doi:10.1095/biolreprod.109.078139.

Figure 1

Click here to access/download;Figure;Figure 1.pdf

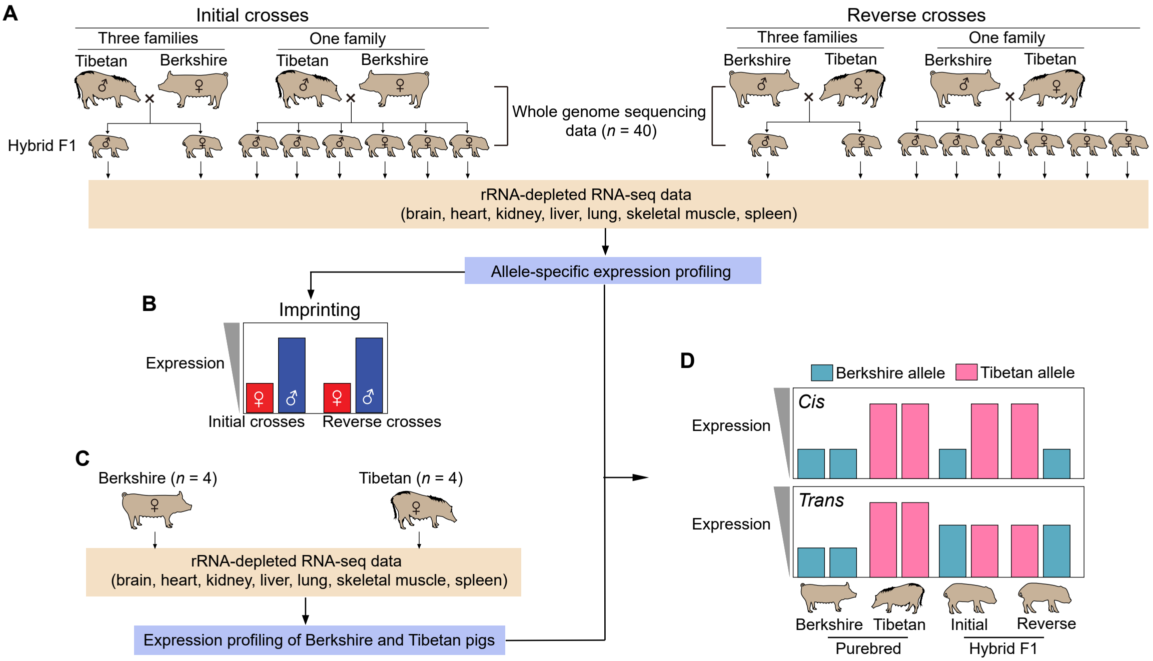

Figure 2

Click here to access/download;Figure;Figure 2.pdf

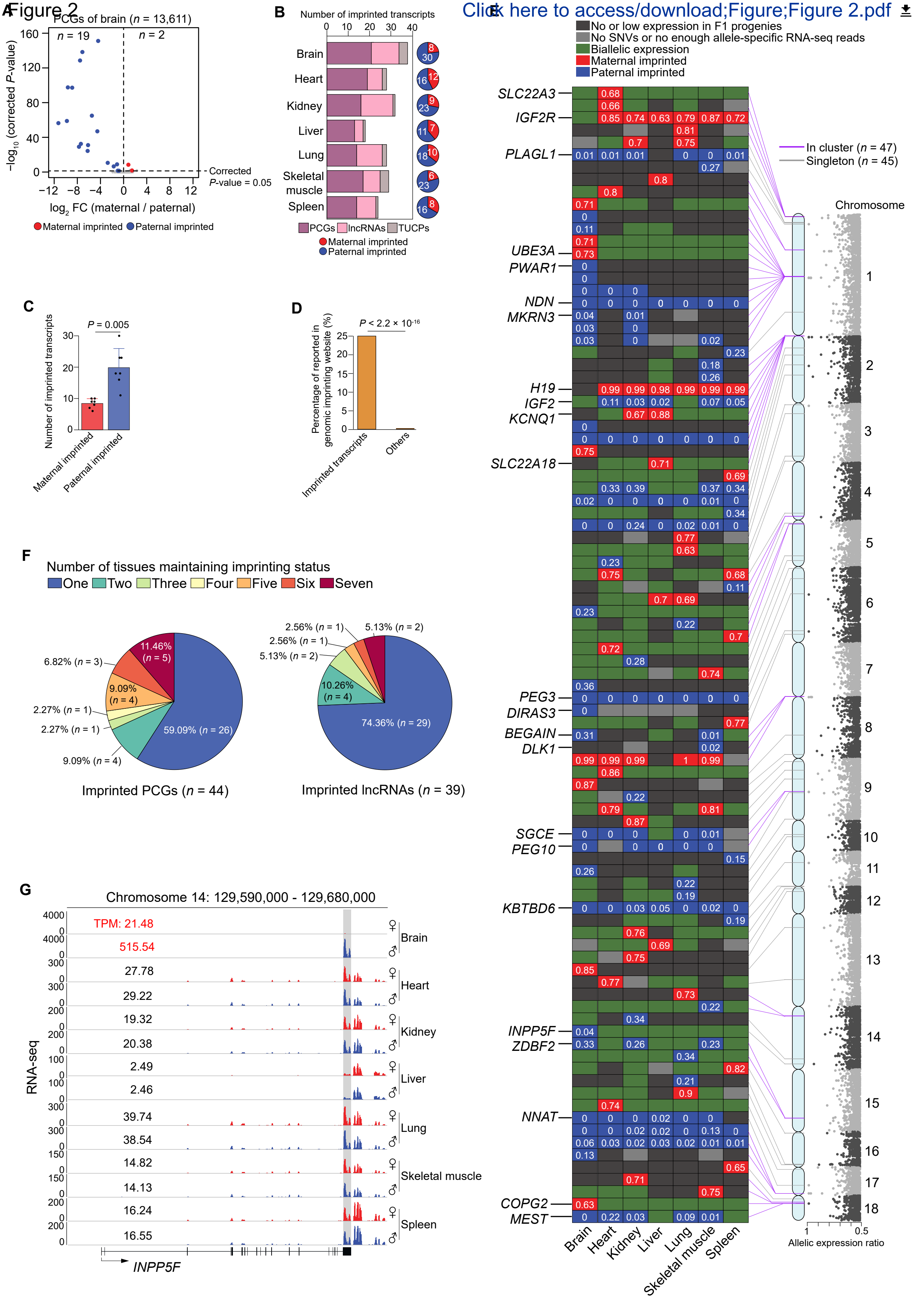

Click here to access/download;Figure;Figure 3.pdf

Figure 3

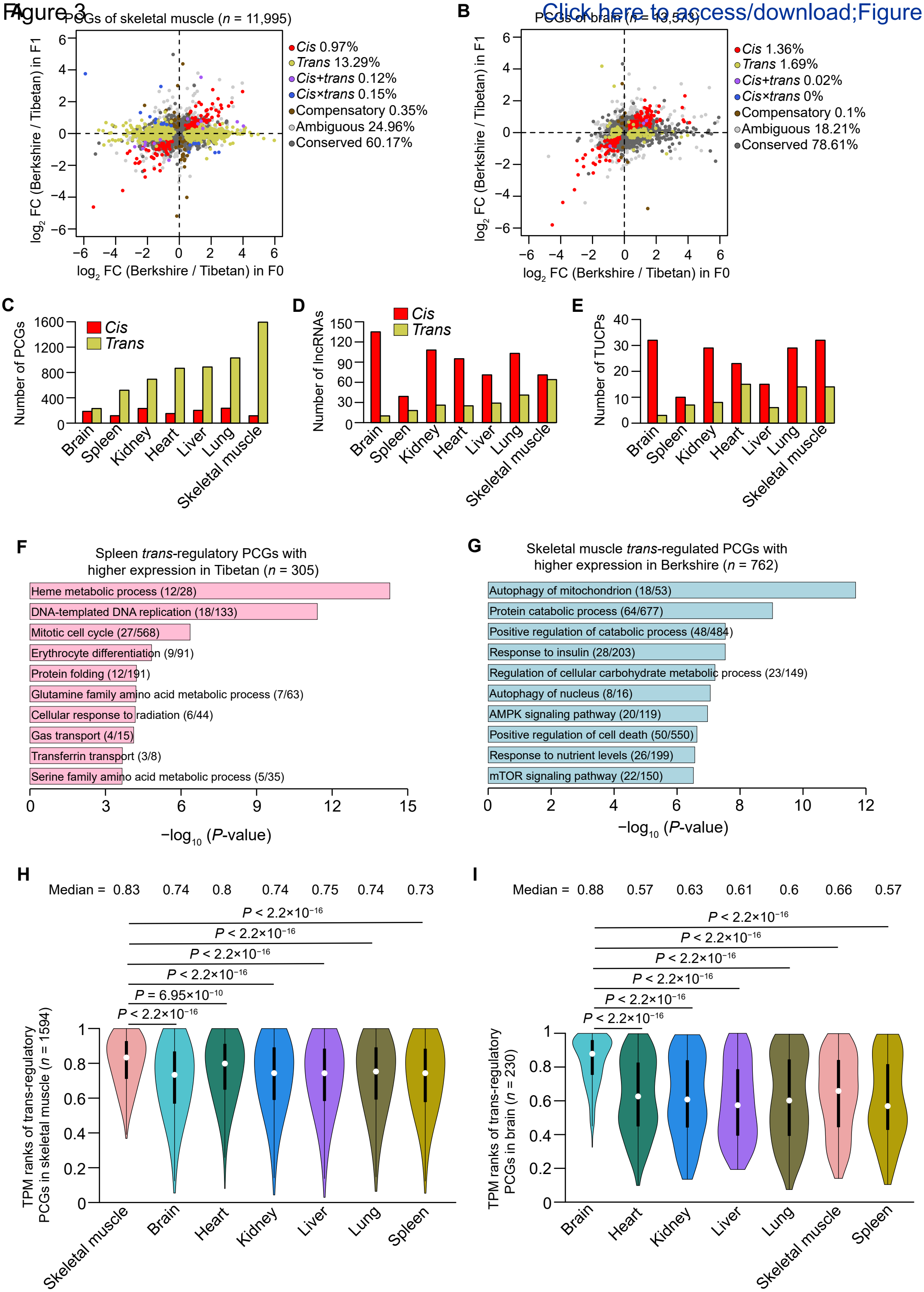

Figure 4

Click here to access/download;Figure;Figure 4.pdf

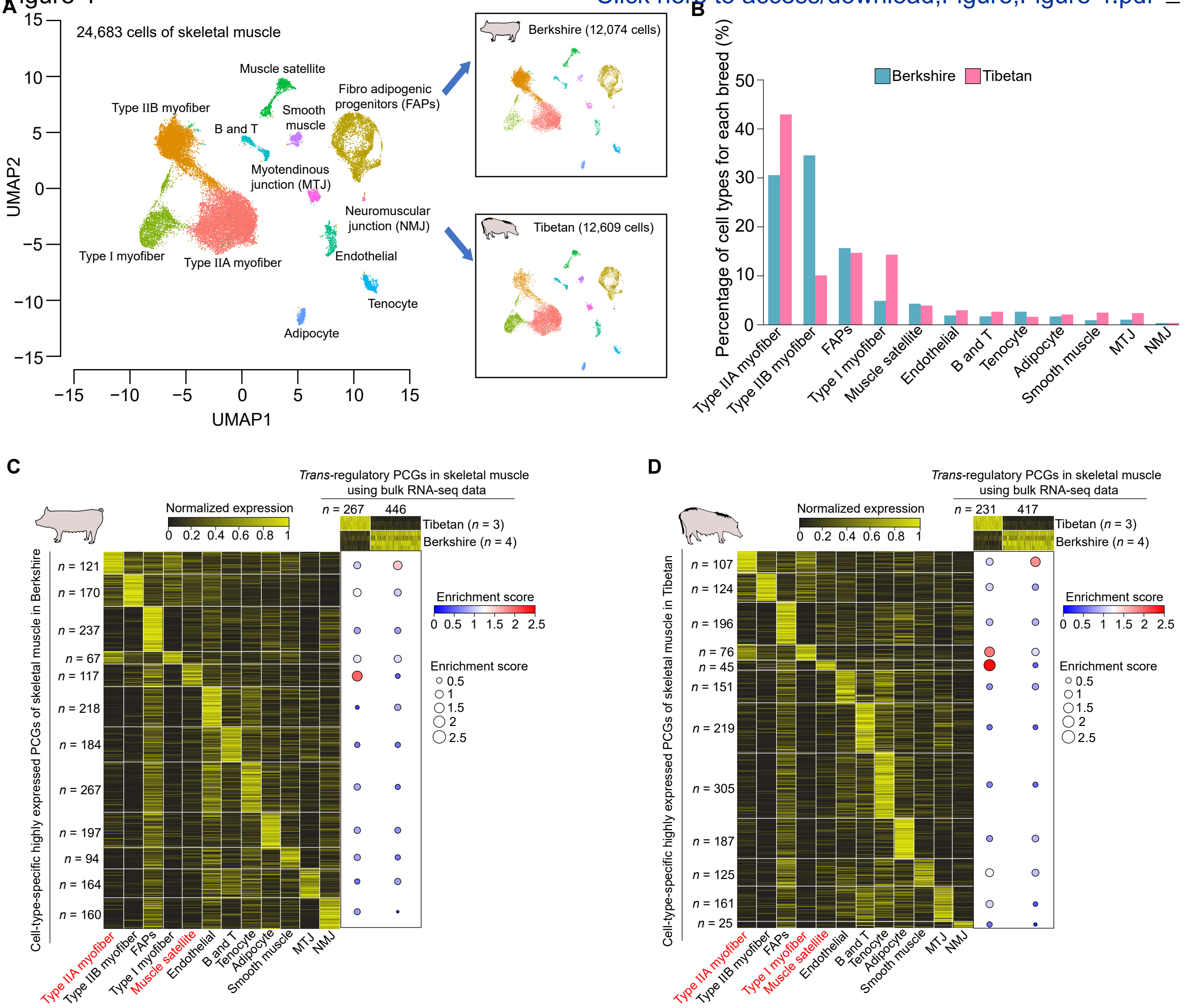

Figure 5

Click here to access/download;Figure;Figure 5.pdf

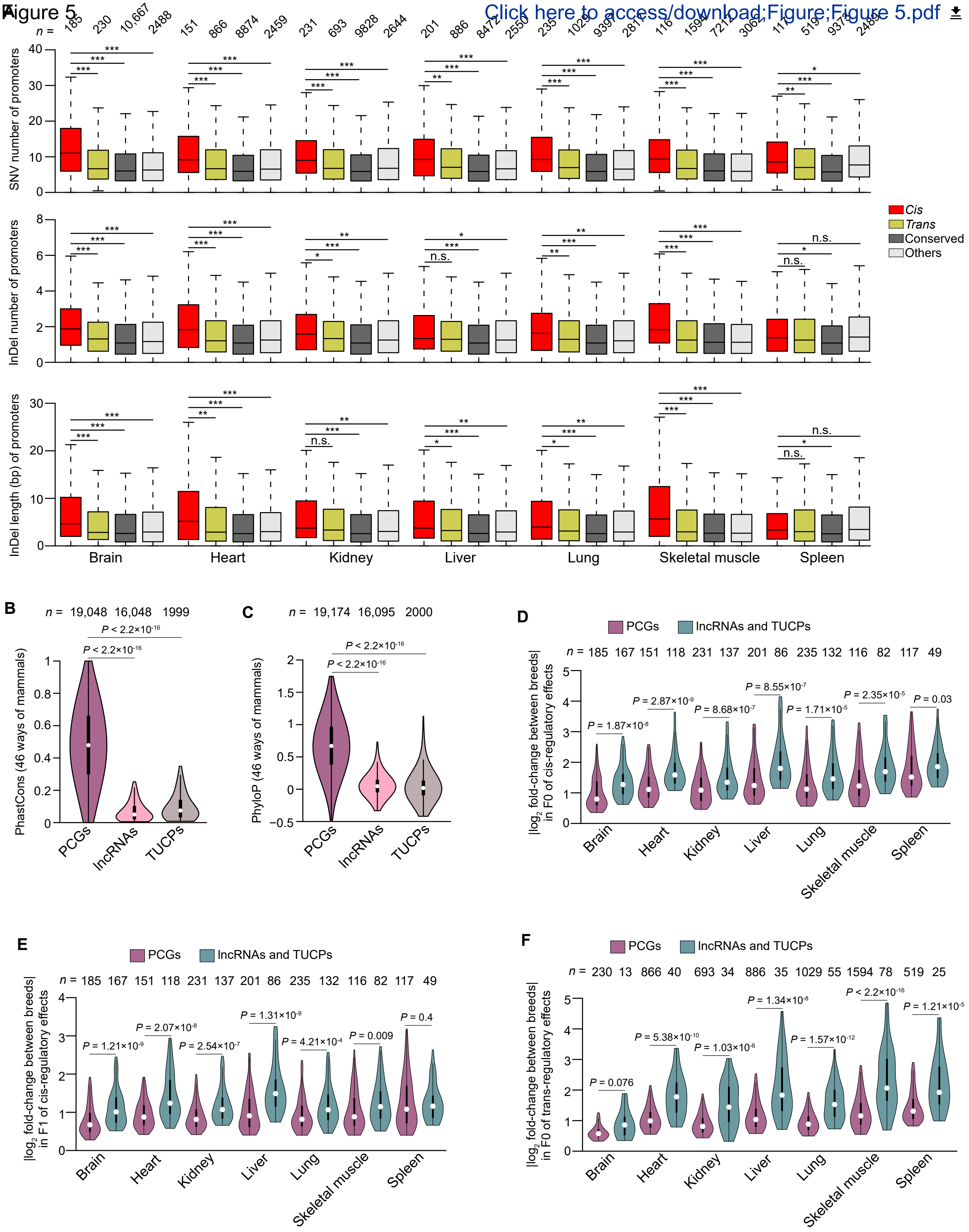

Figure 6

Click here to access/download;Figure;Figure 6.pdf

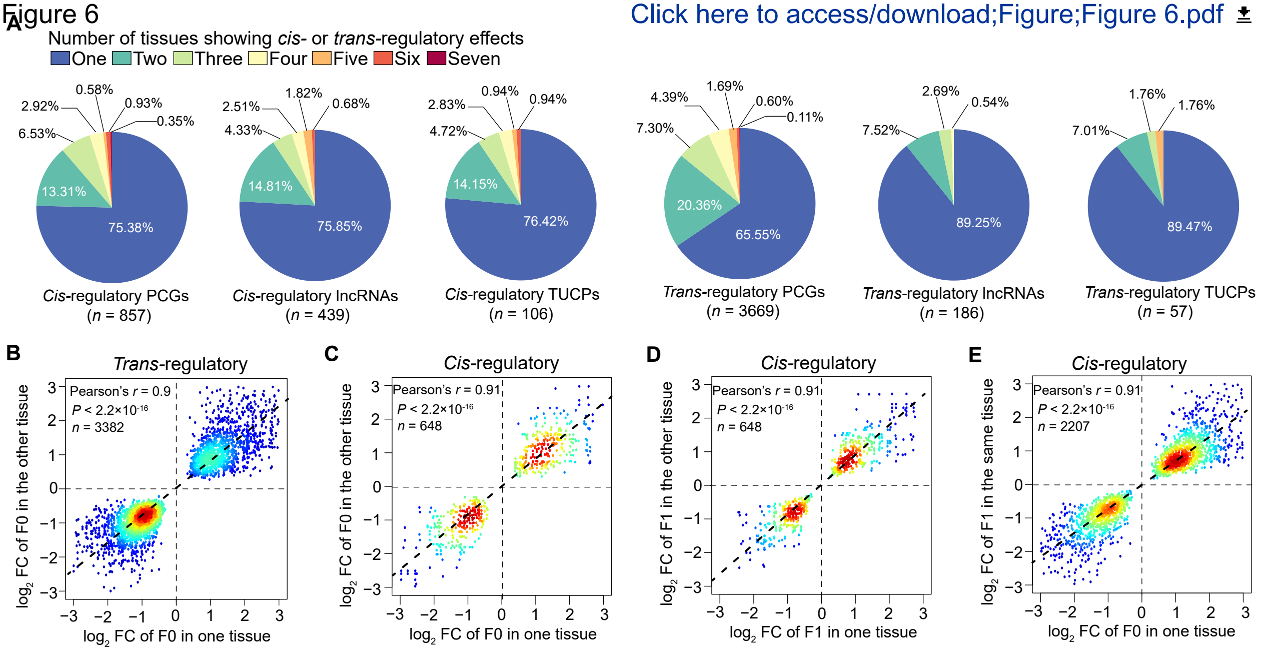

Click here to access/download
Supplementary Material
2. Lin et al. Supplementary Figures.pdf

Supplementary Data S1

Click here to access/download
Supplementary Material
3. Lin et al. Data S1.xlsx

Supplementary Data S2

Click here to access/download
Supplementary Material
4. Lin et al. Data S2.xlsx
