# Supplementary material for: Allele-specific regulatory effects on the pig transcriptome
Source: Gigascience. 2023 Sep 30;12:giad076. doi: 10.1093/gigascience/giad076 (PMC10541795; doi:10.1093/gigascience/giad076)
Supplement: giad076_Supplemental_File [file giad076_supplemental_file.pdf]

Supplementary Figures for

Allele-specific regulatory effects on the pig transcriptome

Yu Lin[1,†], Jing Li[1,2,†,*], Li Chen[3,4,†], Jingyi Bai[1], Jiaman Zhang[1], Yujie Wang[1], Pengliang Liu[1], Keren Long[2], Liangpeng Ge[3,4], Long Jin[2], Yiren Gu[5,6] and Mingzhou Li[1,*]

[1]Livestock and Poultry Multi-omics Key Laboratory of Ministry of Agriculture and Rural Affairs, College of Animal Science and Technology, Sichuan Agricultural University, Chengdu, 611130, China.

[2]Animal Breeding and Genetics Key Laboratory of Sichuan Province, Institute of Animal Genetics and Breeding, Sichuan Agricultural University, Chengdu, 611130, China.

[3]Pig Industry Sciences Key Laboratory of Ministry of Agriculture and Rural Affairs, Chongqing Academy of Animal Sciences, Chongqing, 402460, China.

[4]National Center of Technology Innovation for Pigs, Chongqing, 402460, China.

[5]College of Animal and Veterinary Sciences, Southwest Minzu University, Chengdu, 610041, China.

[6]Animal Breeding and Genetics Key Laboratory of Sichuan Province, Sichuan Animal Science Academy, Chengdu, 610066, China.

[†]These authors contributed equally to this work

[*]Author for correspondence: (Jing Li: lijing_2020@sicau.edu.cn; Mingzhou Li: mingzhou.li@sicau.edu.cn)

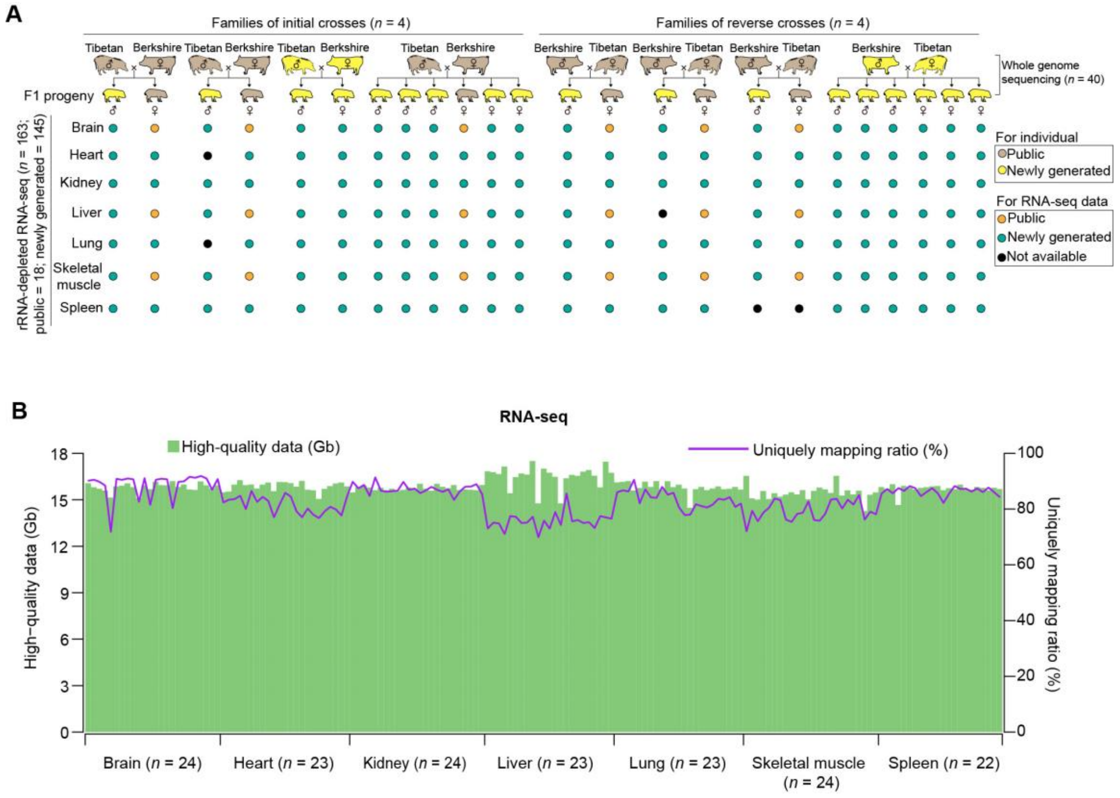

Figure S1. Summary of rRNA-depleted RNA-seq data of F1 progenies. (**A**) A total of 163 rRNA-depleted RNA-seq libraries across seven tissues (brain, heart, kidney, liver, lung, skeletal muscle and spleen) from 24 newly-born F1 progenies generated by reciprocal crosses between Berkshire and Tibetan pigs were collected. Among the 40 individuals, 18 (six parent-child trios, brown) were previously published while the remained 22 individuals (yellow) were newly generated in this study. For RNA-seq data, 18 libraries (circles, orange) were downloaded while the remained 145 (circles, green) were newly generated in this study. The whole genome sequencing data were downloaded (n = 18; brown) and newly generated (n = 22; yellow). See detailed information in Materials and Methods and Supplementary Data S1. (**B**) The high-quality data (bars, green) and uniquely mapping ratio (line, purple) for each RNA-seq library were shown.

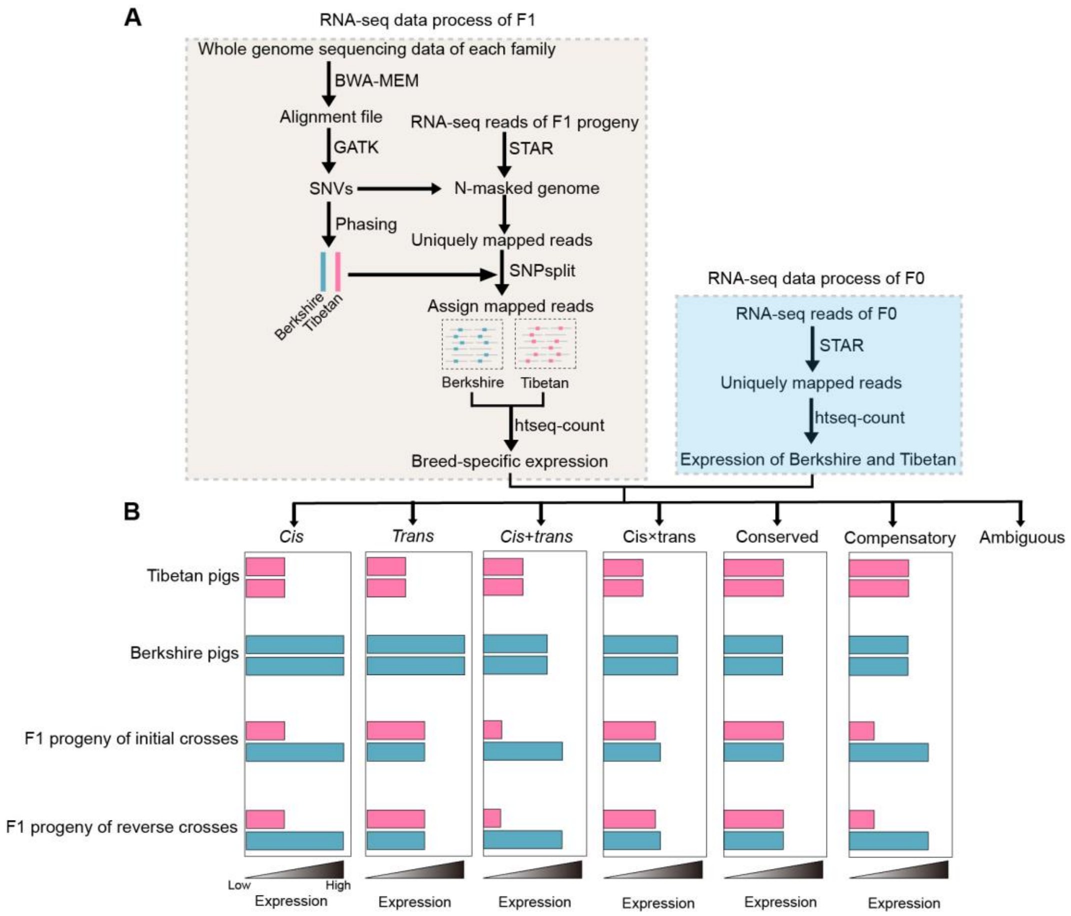

Figure S2. Classification of expression regulatory categories between breeds. (**A**) Bioinformatic pipelines used for calculating breed-specific expression in F1 groups (left) and expression in F0 groups for Berkshire and Tibetan pigs (right). (**B**) Schematic examples illustrating the classification of seven regulatory categories based on the expression difference between breeds in F0 and F1 groups. *Cis*: concordant expression difference between breeds observed in both F0 and F1 groups. *Trans*: expression difference between breeds observed only in the F0 group. *Cis*+*trans*: simultaneous observation of *cis*- and *trans*-regulated effects with the same expression direction toward one breed. *Cis*×*trans*: simultaneous observation of *cis*- and *trans*-regulated effects with distinct expression directions between breeds. Conserved: no expression difference observed between breeds in either group. Compensatory: expression difference observed only in F1 groups. Ambiguous: all other patterns not included in the above six categories.

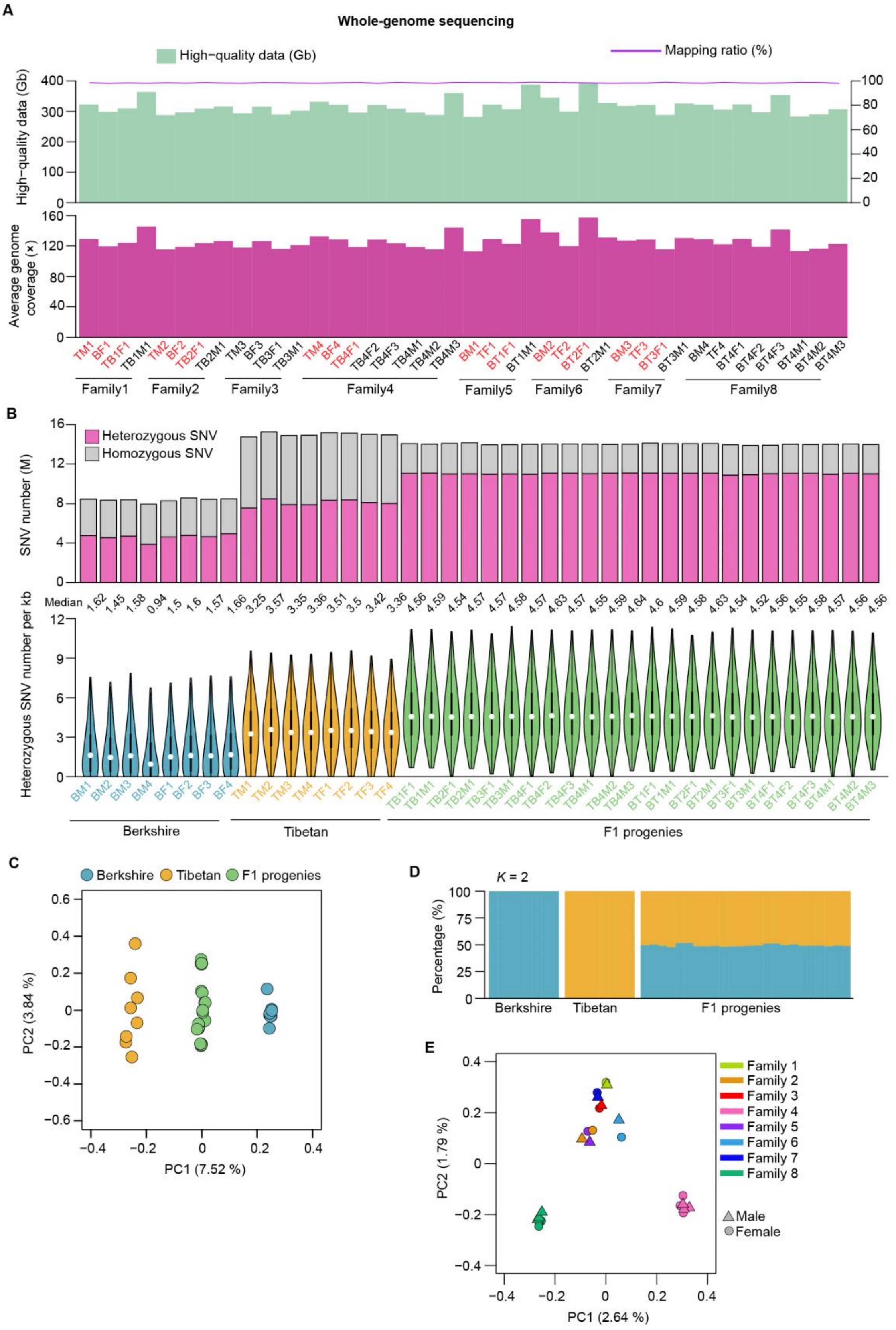

Figure S3. Whole genome sequencing and SNV calling. (**A**) The high-quality data (upper, green bars), mapping ratio (upper, purple lines) and average genome coverage (lower) are shown. The downloaded sample (*n* = 18) were

marked as red. See detailed information in Supplementary Data S1. (**B**) The number of heterozygous and homozygous SNVs (upper, bars) and heterozygous SNV ratio (lower, violins) in each individual for Berkshire pigs, Tibetan pigs and F1 progenies were shown. (**C**) PCA of Berkshire pigs, Tibetan pigs and F1 progenies using SNVs. (**D**) Genetic structure of Berkshire pigs, Tibetan pigs and F1 progenies using SNVs. The percentage of the colored segments represents the proportion of the genome of each individual that originates from the two 'ancestral populations' (*K* = 2, two parental breeds of F1 hybrid pigs, i.e., Berkshire and Tibetan). (**E**) PCA of F1 progenies using SNVs. It revealed the high genetic divergence between different families and similar genetic background for individuals within same family.

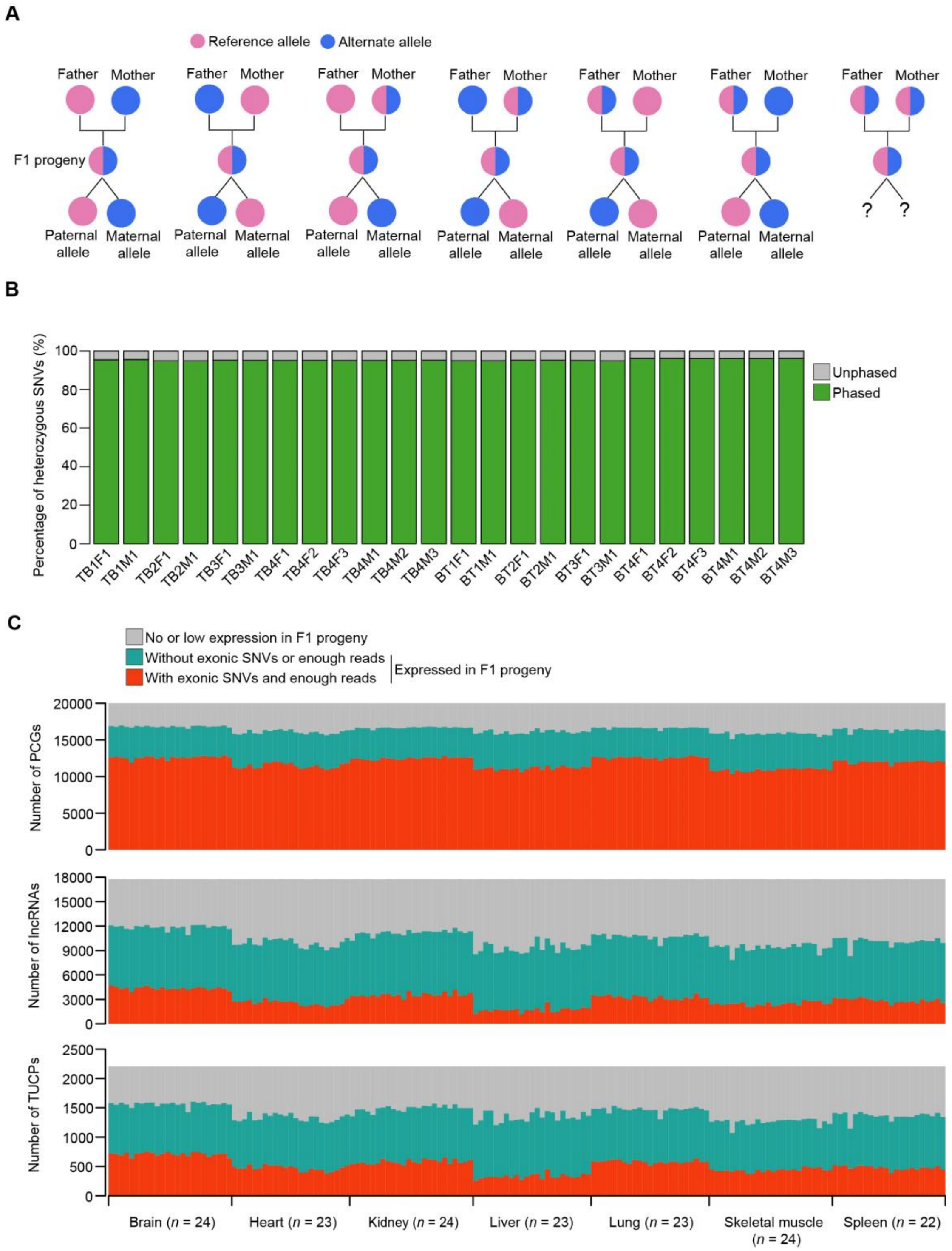

Figure S4. Phasing of heterozygous SNVs and assignment of expression. (**A**) Schematic diagram of heterozygous SNV phasing. The heterozygous SNVs in each F1 progeny can be phased when at least one of the parents is homozygous. (**B**) The proportion of heterozygous SNVs that can be phased in each F1 progeny. (**C**) The number of PCGs (upper), lncRNAs (middle) and TUCPs (lower) with assignment expression to their parental alleles.

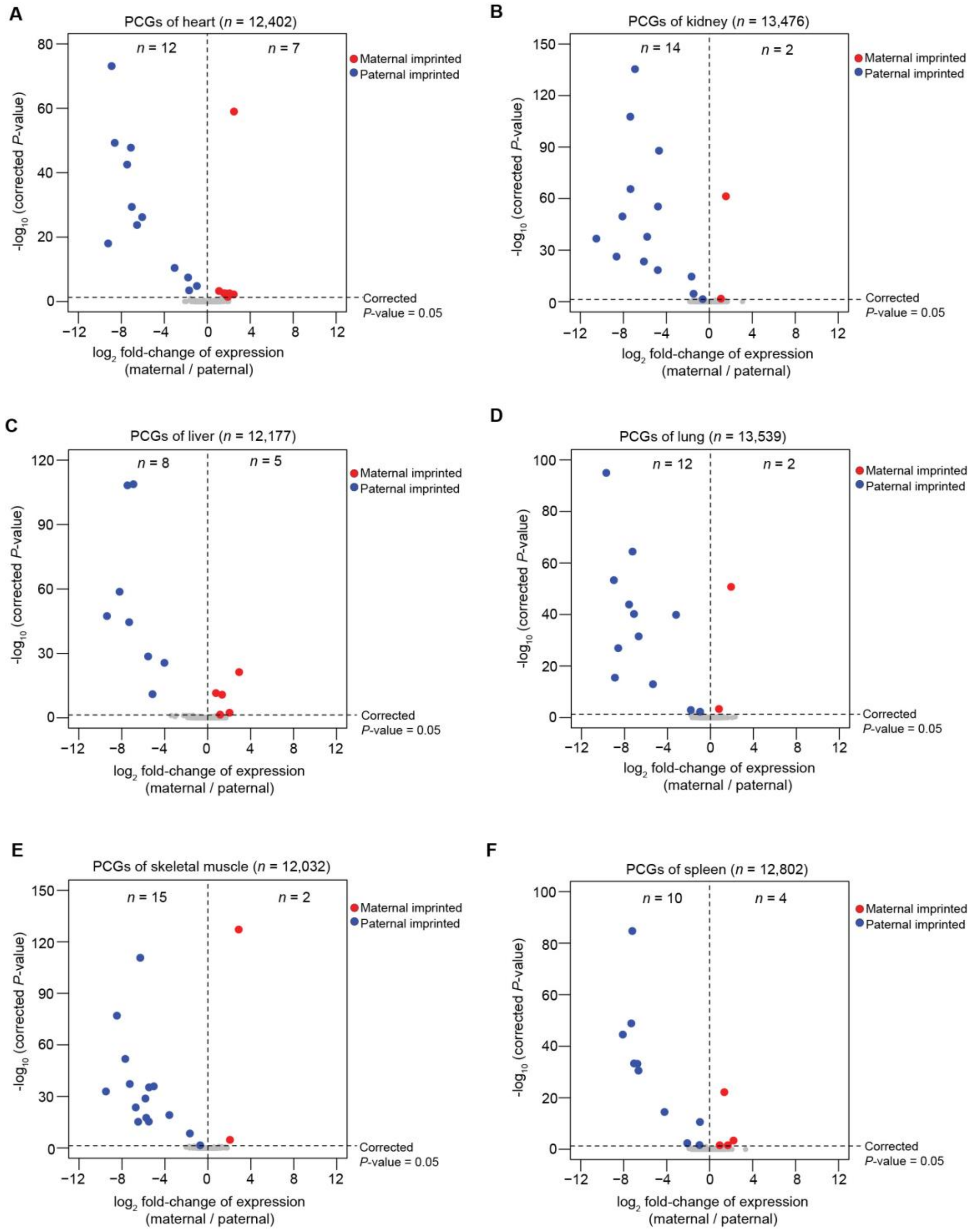

Figure S5. Identification of imprinted PCGs in heart (**A**), kidney (**B**), liver (**C**), lung (**D**), skeletal muscle (**E**) and spleen (**F**). The number of total testable (top), paternal (left) and maternal (right) imprinted PCGs were shown for each tissue.

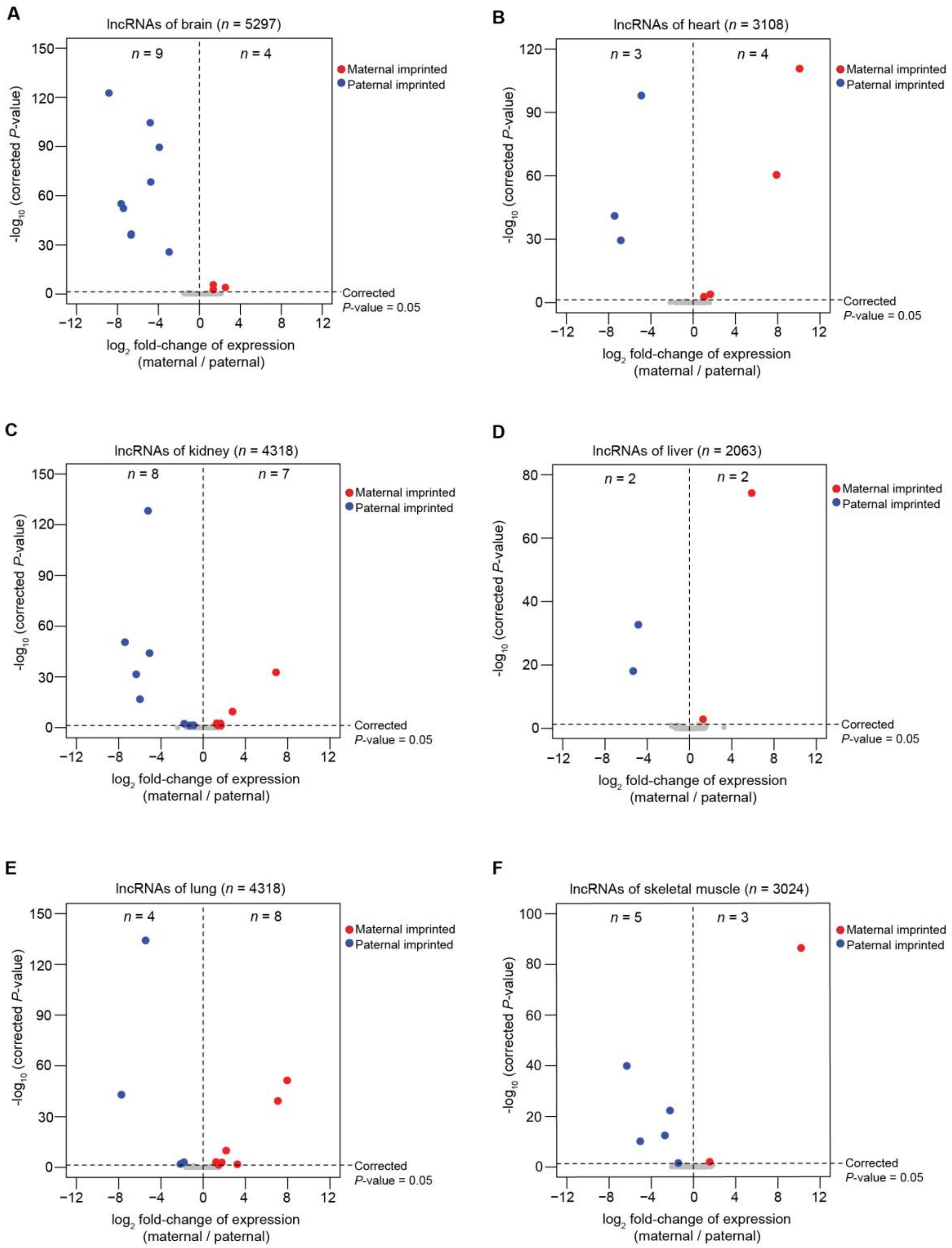
A
lncRNAs of brain (n = 5297)
n = 9
n = 4
Maternal imprinted
Paternal imprinted
-log10 (corrected P-value)
Corrected P-value = 0.05
log2 fold-change of expression (maternal / paternal)
B
lncRNAs of heart (n = 3108)
n = 3
n = 4
C
lncRNAs of kidney (n = 4318)
n = 8
n = 7
D
lncRNAs of liver (n = 2063)
n = 2
n = 2
E
lncRNAs of lung (n = 4318)
n = 4
n = 8
F
lncRNAs of skeletal muscle (n = 3024)
n = 5
n = 3

G

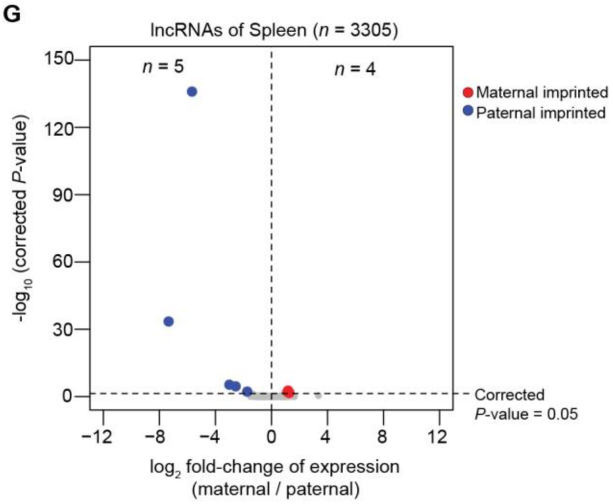

Figure S6. Identification of imprinted lncRNAs in brain (**A**), heart (**B**), kidney (**C**), liver (**D**), lung (**E**), skeletal muscle (**F**) and spleen (**G**). The number of total testable (top), paternal (left) and maternal (right) imprinted lncRNAs were shown for each tissue.

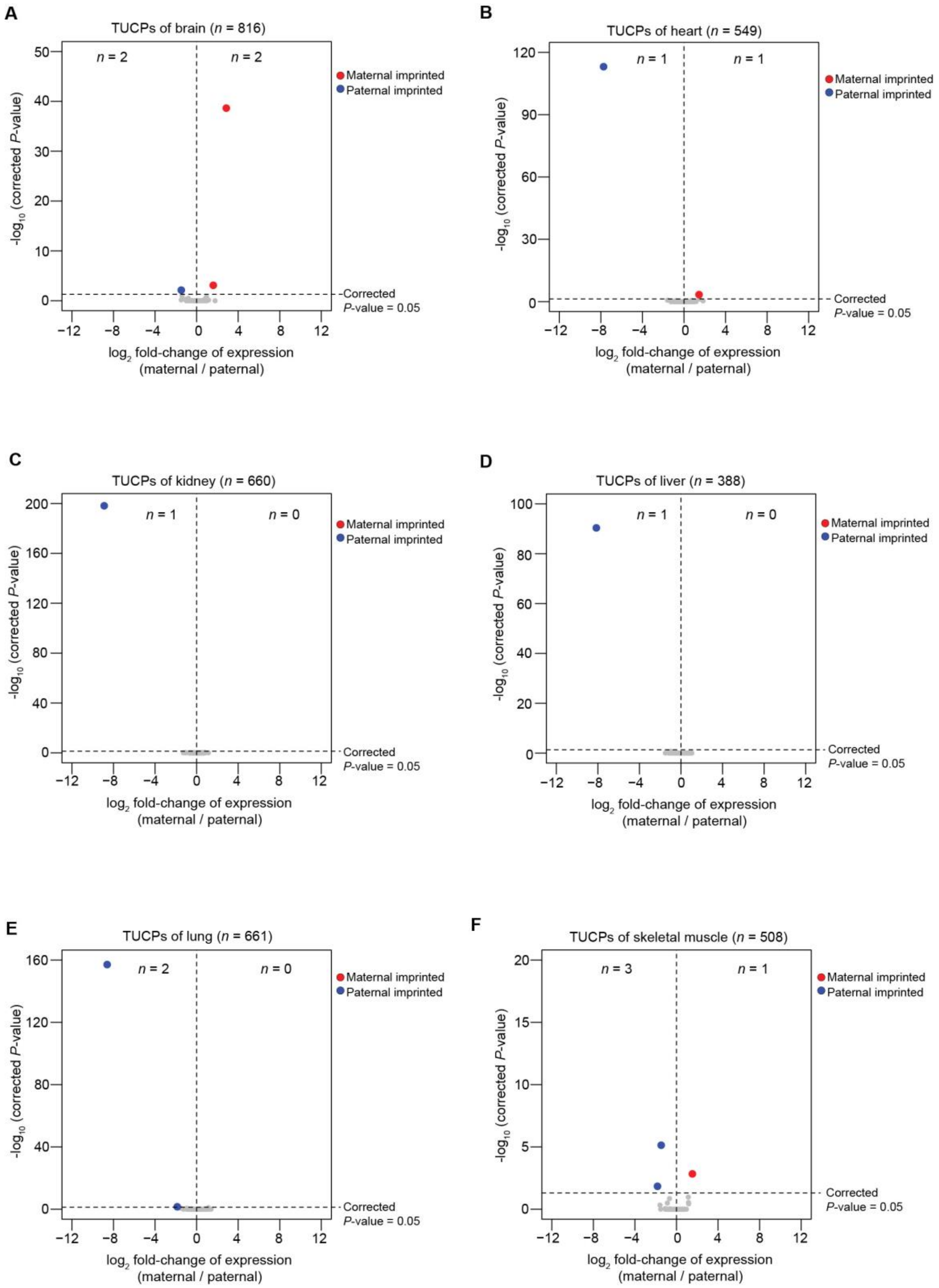
A
TUCPs of brain (n = 816)
n = 2
n = 2
Maternal imprinted
Paternal imprinted
-log10 (corrected P-value)
log2 fold-change of expression (maternal / paternal)
Corrected P-value = 0.05
B
TUCPs of heart (n = 549)
n = 1
n = 1
C
TUCPs of kidney (n = 660)
n = 1
n = 0
D
TUCPs of liver (n = 388)
n = 1
n = 0
E
TUCPs of lung (n = 661)
n = 2
n = 0
F
TUCPs of skeletal muscle (n = 508)
n = 3
n = 1

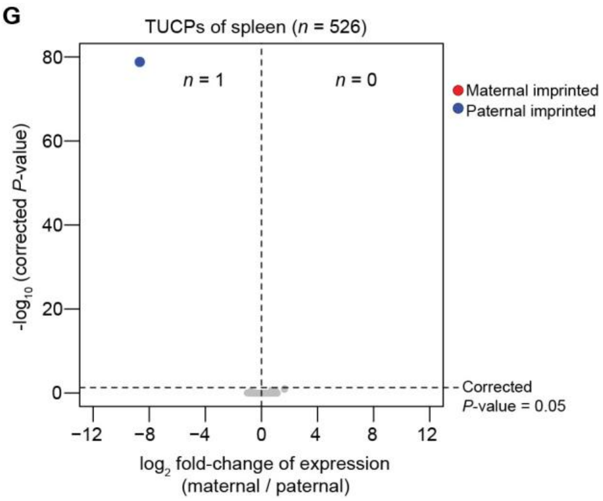

Figure S7. Identification of imprinted TUCPs in brain (**A**), heart (**B**), kidney (**C**), liver (**D**), lung (**E**), skeletal muscle (**F**) and spleen (**G**). The number of total testable (top), paternal (left) and maternal (right) imprinted TUCPs were shown for each tissue.

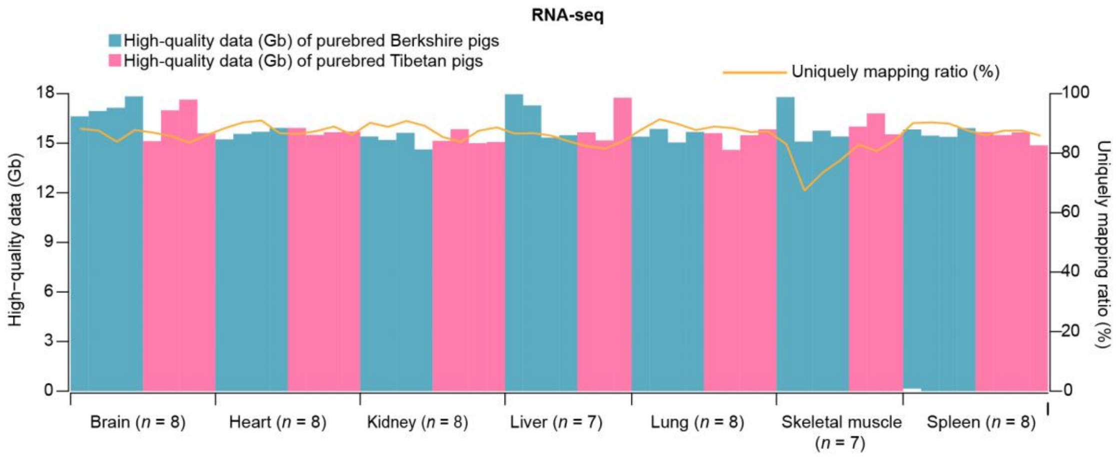

Figure S8. Summary of rRNA-depleted RNA-seq data of purebred Berkshire and Tibetan pigs. A total of 54 samples from eight newly-born pigs (Berkshire [*n* = 4] and Tibetan [*n* = 4]) were collected to perform rRNA-depleted RNA-seq, including brain (*n* = 8), heart (*n* = 8), kidney (*n* = 8), liver (*n* = 7), lung (*n* = 8), skeletal muscle (*n*= 7) and spleen (*n* = 8). The high-quality data (bars, blue and pink) and uniquely mapping ratio (line, orange) for each sample were shown.

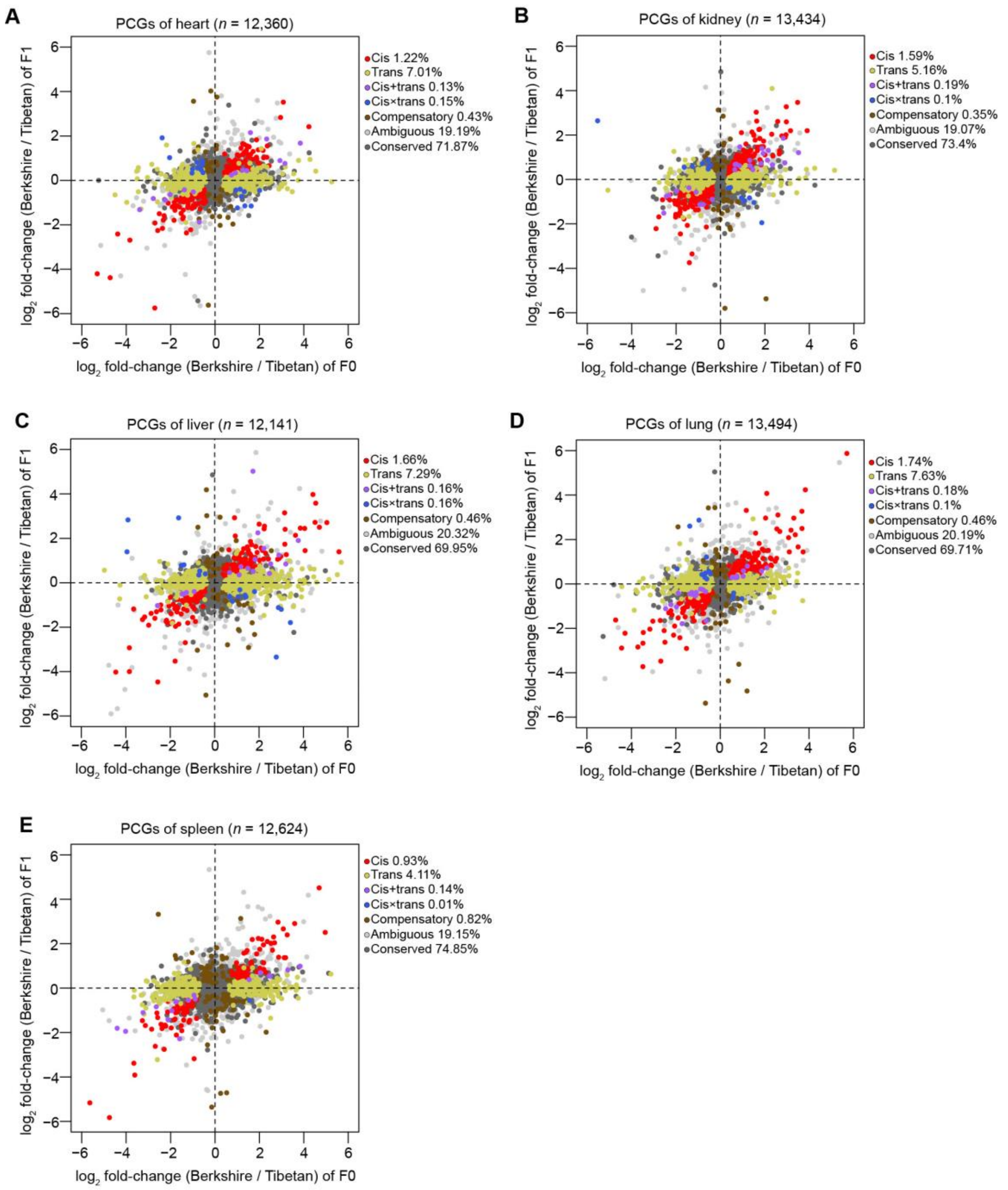

Figure S9. Classification of *cis*- and *trans*-regulated effects of PCGs in heart (**A**), kidney (**B**), liver (**C**), lung (**D**) and spleen (**E**). The X and Y axis represents the $\log_2$ fold-change of expression (Berkshire / Tibetan) in F0 and F1 groups, respectively. The number of testable PCGs (top) and the ratio of each regulatory category (right) were shown for each tissue.

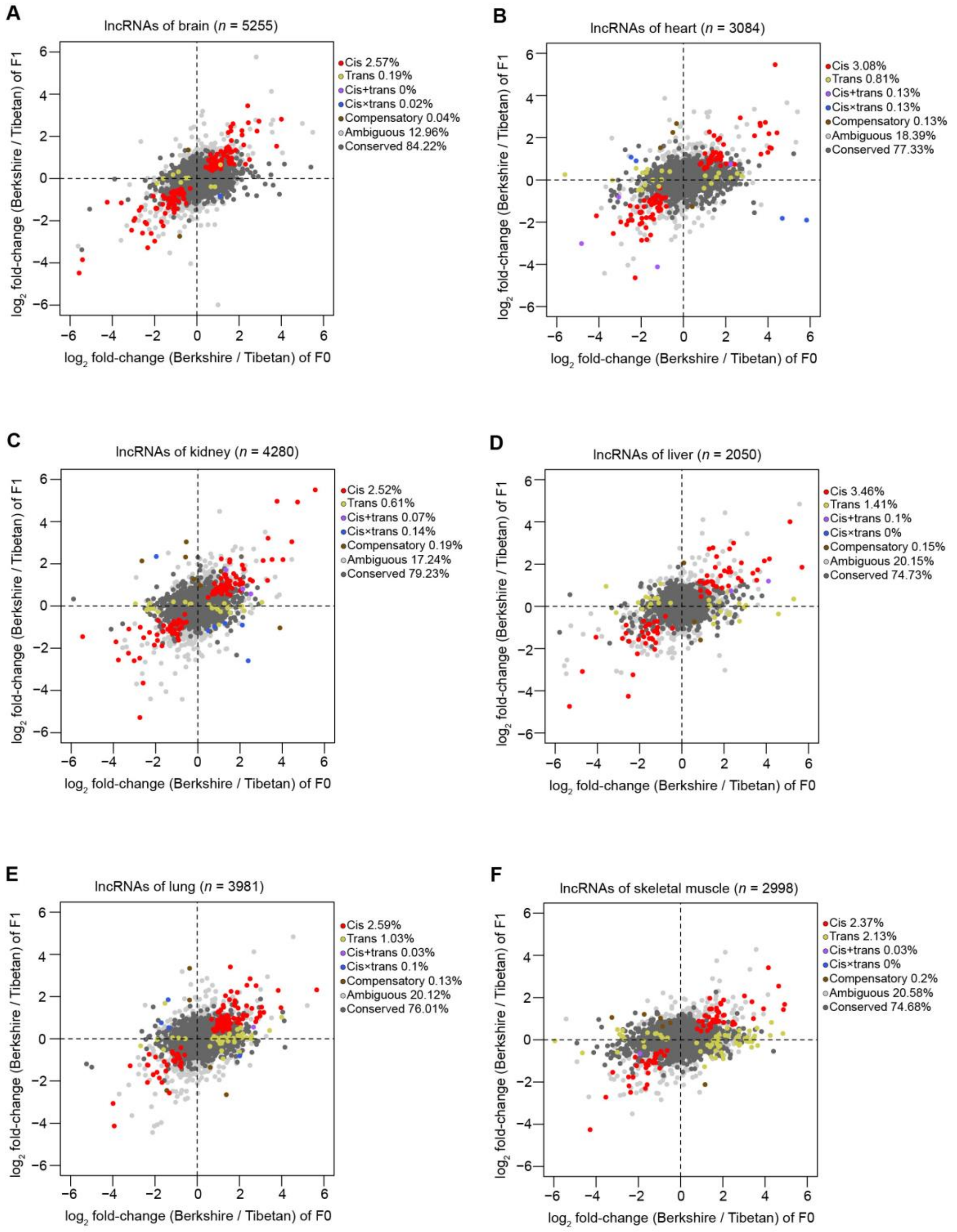
A
lncRNAs of brain (n = 5255)
Cis 2.57%
Trans 0.19%
Cis+trans 0%
Cis×trans 0.02%
Compensatory 0.04%
Ambiguous 12.96%
Conserved 84.22%
B
lncRNAs of heart (n = 3084)
Cis 3.08%
Trans 0.81%
Cis+trans 0.13%
Cis×trans 0.13%
Compensatory 0.13%
Ambiguous 18.39%
Conserved 77.33%
C
lncRNAs of kidney (n = 4280)
Cis 2.52%
Trans 0.61%
Cis+trans 0.07%
Cis×trans 0.14%
Compensatory 0.19%
Ambiguous 17.24%
Conserved 79.23%
D
lncRNAs of liver (n = 2050)
Cis 3.46%
Trans 1.41%
Cis+trans 0.1%
Cis×trans 0%
Compensatory 0.15%
Ambiguous 20.15%
Conserved 74.73%
E
lncRNAs of lung (n = 3981)
Cis 2.59%
Trans 1.03%
Cis+trans 0.03%
Cis×trans 0.1%
Compensatory 0.13%
Ambiguous 20.12%
Conserved 76.01%
F
lncRNAs of skeletal muscle (n = 2998)
Cis 2.37%
Trans 2.13%
Cis+trans 0.03%
Cis×trans 0%
Compensatory 0.2%
Ambiguous 20.58%
Conserved 74.68%
log2 fold-change (Berkshire / Tibetan) of F1
log2 fold-change (Berkshire / Tibetan) of F0
-6 -4 -2 0 2 4 6

G

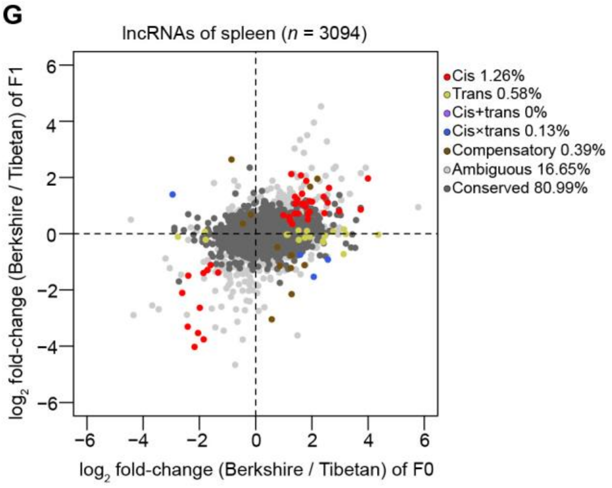

Figure S10. Classification of *cis*- and *trans*-regulated effects of lncRNAs in brain (**A**), heart (**B**), kidney (**C**), liver (**D**), lung (**E**), skeletal muscle (**F**) and spleen (**G**). The X and Y axis represents the $\log_2$ fold-change of expression (Berkshire / Tibetan) in F0 and F1 groups, respectively. The number of testable lncRNAs (top) and the ratio of each regulatory category (right) were shown for each tissue.

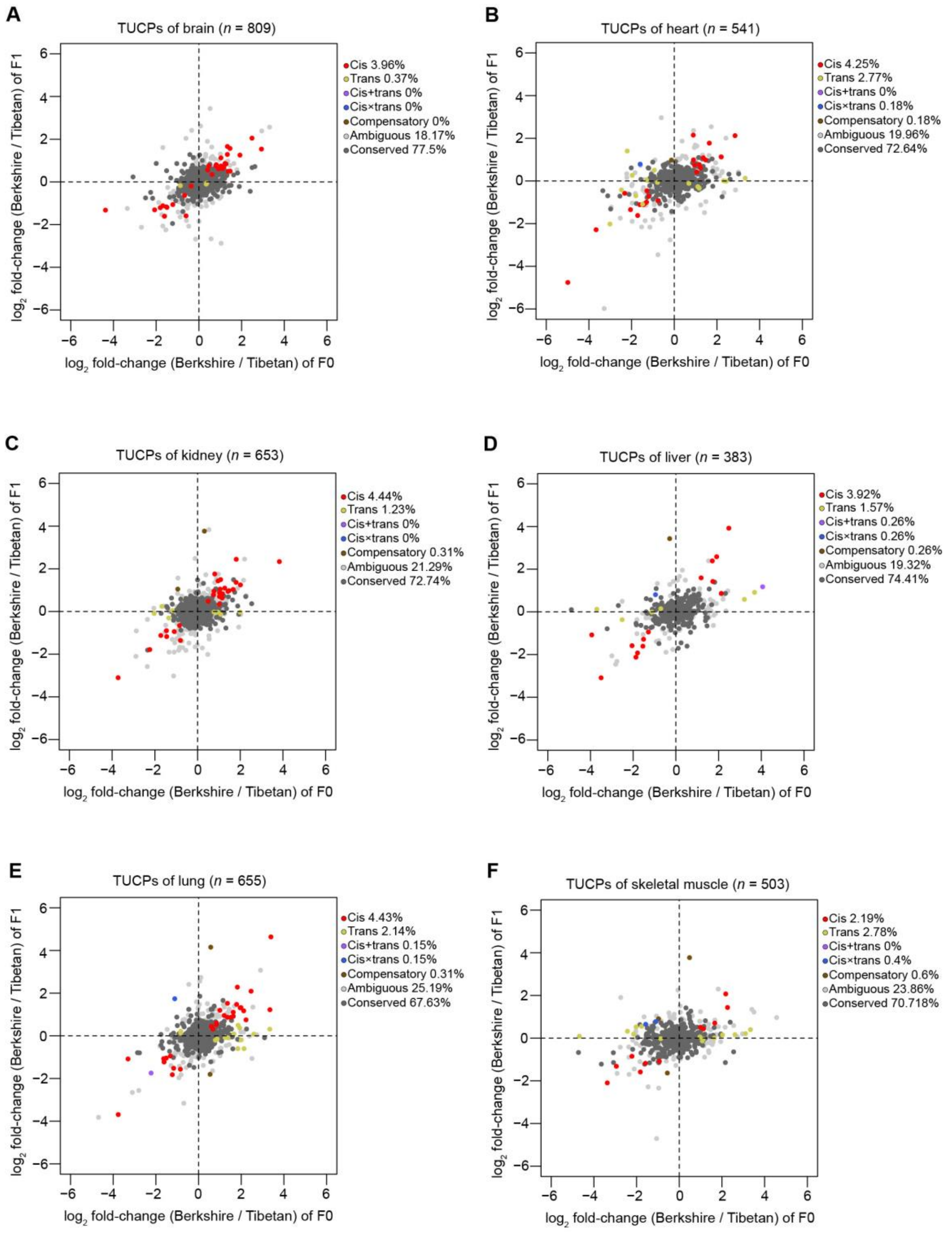

A
TUCPs of brain (n = 809)
Cis 3.96%
Trans 0.37%
Cis+trans 0%
Cis×trans 0%
Compensatory 0%
Ambiguous 18.17%
Conserved 77.5%
B
TUCPs of heart (n = 541)
Cis 4.25%
Trans 2.77%
Cis+trans 0%
Cis×trans 0.18%
Compensatory 0.18%
Ambiguous 19.96%
Conserved 72.64%
C
TUCPs of kidney (n = 653)
Cis 4.44%
Trans 1.23%
Cis+trans 0%
Cis×trans 0%
Compensatory 0.31%
Ambiguous 21.29%
Conserved 72.74%
D
TUCPs of liver (n = 383)
Cis 3.92%
Trans 1.57%
Cis+trans 0.26%
Cis×trans 0.26%
Compensatory 0.26%
Ambiguous 19.32%
Conserved 74.41%
E
TUCPs of lung (n = 655)
Cis 4.43%
Trans 2.14%
Cis+trans 0.15%
Cis×trans 0.15%
Compensatory 0.31%
Ambiguous 25.19%
Conserved 67.63%
F
TUCPs of skeletal muscle (n = 503)
Cis 2.19%
Trans 2.78%
Cis+trans 0%
Cis×trans 0.4%
Compensatory 0.6%
Ambiguous 23.86%
Conserved 70.718%
log2 fold-change (Berkshire / Tibetan) of F0
log2 fold-change (Berkshire / Tibetan) of F1
-6 -4 -2 0 2 4 6

G

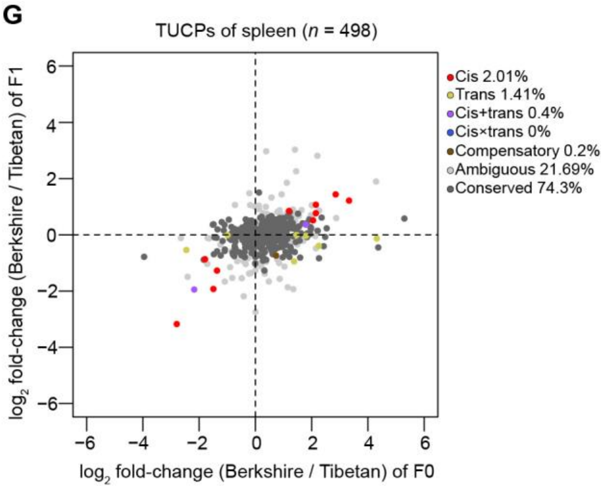

Figure S11. Classification of *cis*- and *trans*-regulated effects of TUCPs in brain (**A**), heart (**B**), kidney (**C**), liver (**D**), lung (**E**), skeletal muscle (**F**) and spleen (**G**). The X and Y axis represents the $\log_2$ fold-change of expression (Berkshire / Tibetan) in F0 and F1 groups, respectively. The number of testable TUCPs (top) and the ratio of each regulatory category (right) were shown for each tissue.

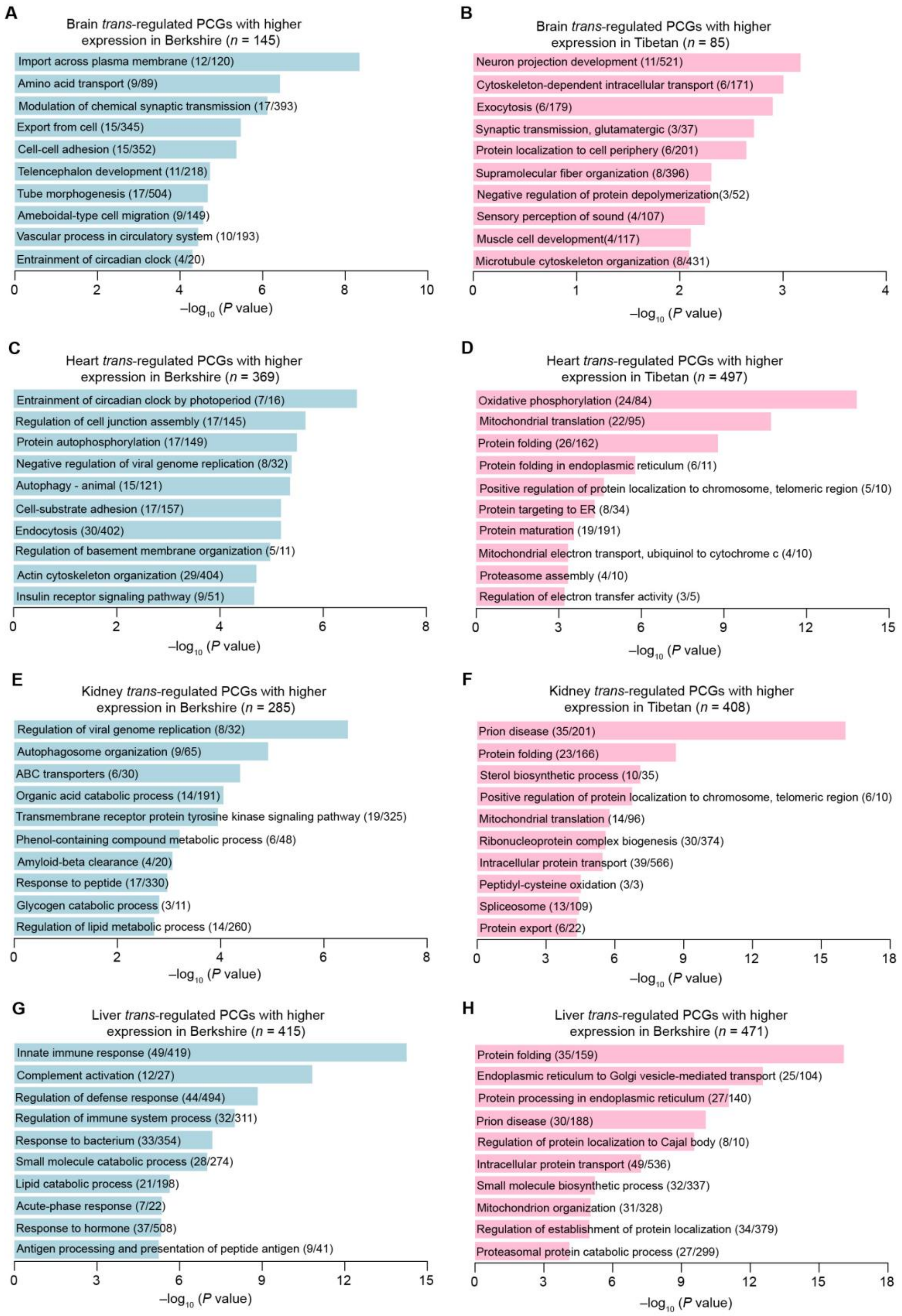

A
Brain trans-regulated PCGs with higher expression in Berkshire (n = 145)
Import across plasma membrane (12/120)
Amino acid transport (9/89)
Modulation of chemical synaptic transmission (17/393)
Export from cell (15/345)
Cell-cell adhesion (15/352)
Telencephalon development (11/218)
Tube morphogenesis (17/504)
Ameboidal-type cell migration (9/149)
Vascular process in circulatory system (10/193)
Entrainment of circadian clock (4/20)
0 2 4 6 8 10
–log10 (P value)
B
Brain trans-regulated PCGs with higher expression in Tibetan (n = 85)
Neuron projection development (11/521)
Cytoskeleton-dependent intracellular transport (6/171)
Exocytosis (6/179)
Synaptic transmission, glutamatergic (3/37)
Protein localization to cell periphery (6/201)
Supramolecular fiber organization (8/396)
Negative regulation of protein depolymerization(3/52)
Sensory perception of sound (4/107)
Muscle cell development(4/117)
Microtubule cytoskeleton organization (8/431)
0 1 2 3 4
–log10 (P value)
C
Heart trans-regulated PCGs with higher expression in Berkshire (n = 369)
Entrainment of circadian clock by photoperiod (7/16)
Regulation of cell junction assembly (17/145)
Protein autophosphorylation (17/149)
Negative regulation of viral genome replication (8/32)
Autophagy - animal (15/121)
Cell-substrate adhesion (17/157)
Endocytosis (30/402)
Regulation of basement membrane organization (5/11)
Actin cytoskeleton organization (29/404)
Insulin receptor signaling pathway (9/51)
0 2 4 6 8
–log10 (P value)
D
Heart trans-regulated PCGs with higher expression in Tibetan (n = 497)
Oxidative phosphorylation (24/84)
Mitochondrial translation (22/95)
Protein folding (26/162)
Protein folding in endoplasmic reticulum (6/11)
Positive regulation of protein localization to chromosome, telomeric region (5/10)
Protein targeting to ER (8/34)
Protein maturation (19/191)
Mitochondrial electron transport, ubiquinol to cytochrome c (4/10)
Proteasome assembly (4/10)
Regulation of electron transfer activity (3/5)
0 3 6 9 12 15
–log10 (P value)
E
Kidney trans-regulated PCGs with higher expression in Berkshire (n = 285)
Regulation of viral genome replication (8/32)
Autophagosome organization (9/65)
ABC transporters (6/30)
Organic acid catabolic process (14/191)
Transmembrane receptor protein tyrosine kinase signaling pathway (19/325)
Phenol-containing compound metabolic process (6/48)
Amyloid-beta clearance (4/20)
Response to peptide (17/330)
Glycogen catabolic process (3/11)
Regulation of lipid metabolic process (14/260)
0 2 4 6 8
–log10 (P value)
F
Kidney trans-regulated PCGs with higher expression in Tibetan (n = 408)
Prion disease (35/201)
Protein folding (23/166)
Sterol biosynthetic process (10/35)
Positive regulation of protein localization to chromosome, telomeric region (6/10)
Mitochondrial translation (14/96)
Ribonucleoprotein complex biogenesis (30/374)
Intracellular protein transport (39/566)
Peptidyl-cysteine oxidation (3/3)
Spliceosome (13/109)
Protein export (6/22)
0 3 6 9 12 15 18
–log10 (P value)
G
Liver trans-regulated PCGs with higher expression in Berkshire (n = 415)
Innate immune response (49/419)
Complement activation (12/27)
Regulation of defense response (44/494)
Regulation of immune system process (32/311)
Response to bacterium (33/354)
Small molecule catabolic process (28/274)
Lipid catabolic process (21/198)
Acute-phase response (7/22)
Response to hormone (37/508)
Antigen processing and presentation of peptide antigen (9/41)
0 3 6 9 12 15
–log10 (P value)
H
Liver trans-regulated PCGs with higher expression in Berkshire (n = 471)
Protein folding (35/159)
Endoplasmic reticulum to Golgi vesicle-mediated transport (25/104)
Protein processing in endoplasmic reticulum (27/140)
Prion disease (30/188)
Regulation of protein localization to Cajal body (8/10)
Intracellular protein transport (49/536)
Small molecule biosynthetic process (32/337)
Mitochondrion organization (31/328)
Regulation of establishment of protein localization (34/379)
Proteasomal protein catabolic process (27/299)
0 3 6 9 12 15 18
–log10 (P value)

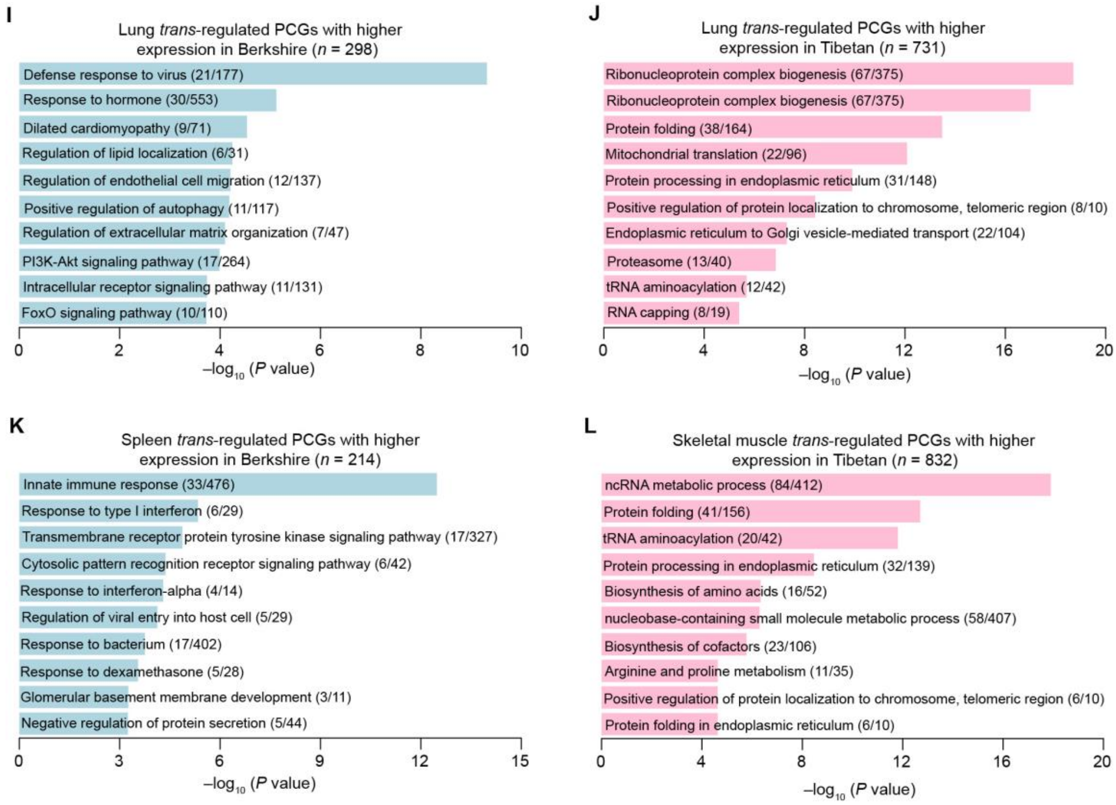

Figure S12. GO and KEGG enrichment for *trans*-regulated PCGs with higher expression in Berkshire (blue) and Tibetan (pink) for each tissue.

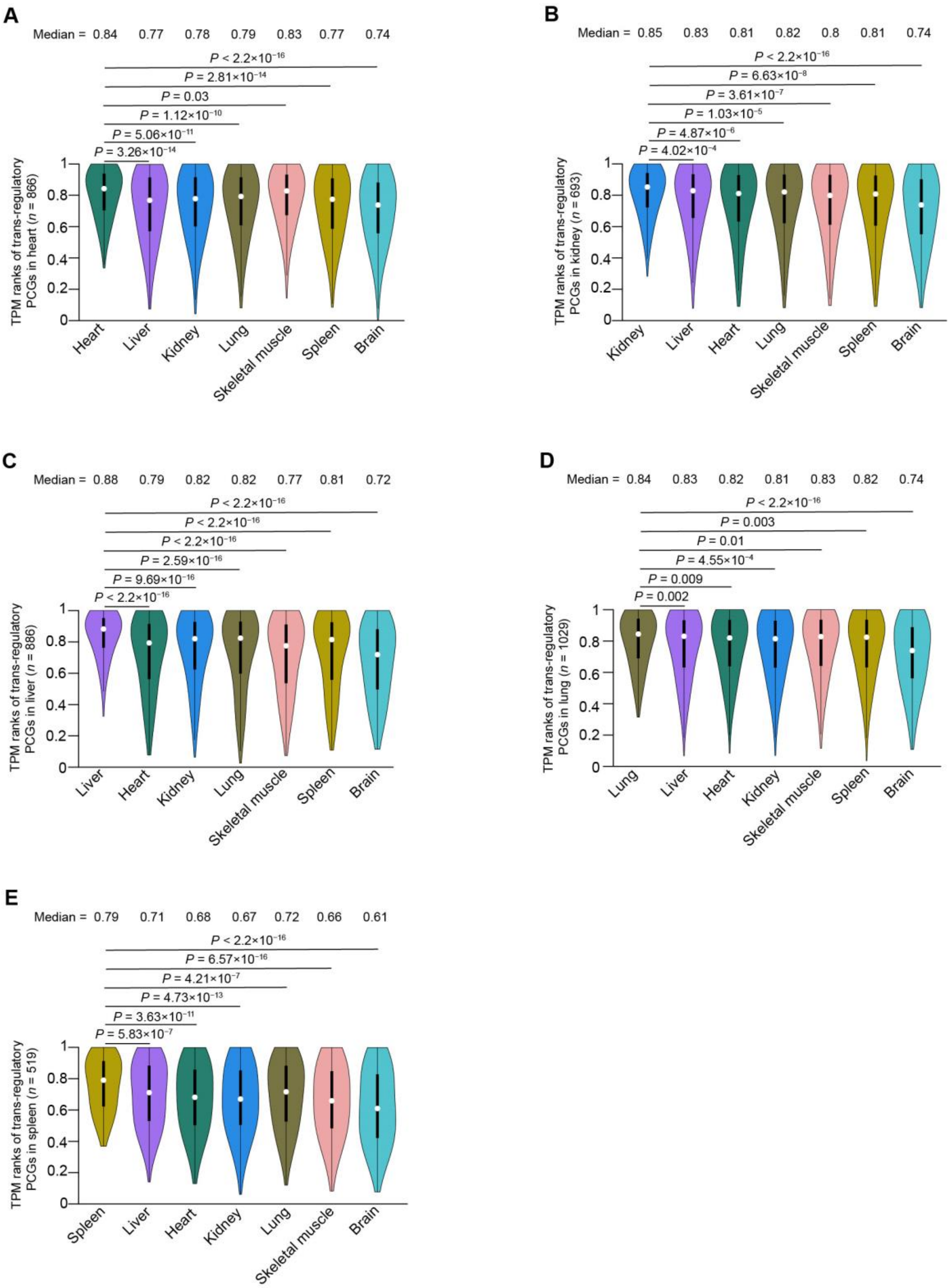

Figure S13. The TPM ranks of each tissue for *trans*-regulated PCGs in heart (**A**), kidney (**B**), liver (**C**), lung (**D**) and spleen (**E**). The ranks represented the normalized expression level in each tissue from low to high. The *P*-values were calculated by Wilcoxon rank-sum test.

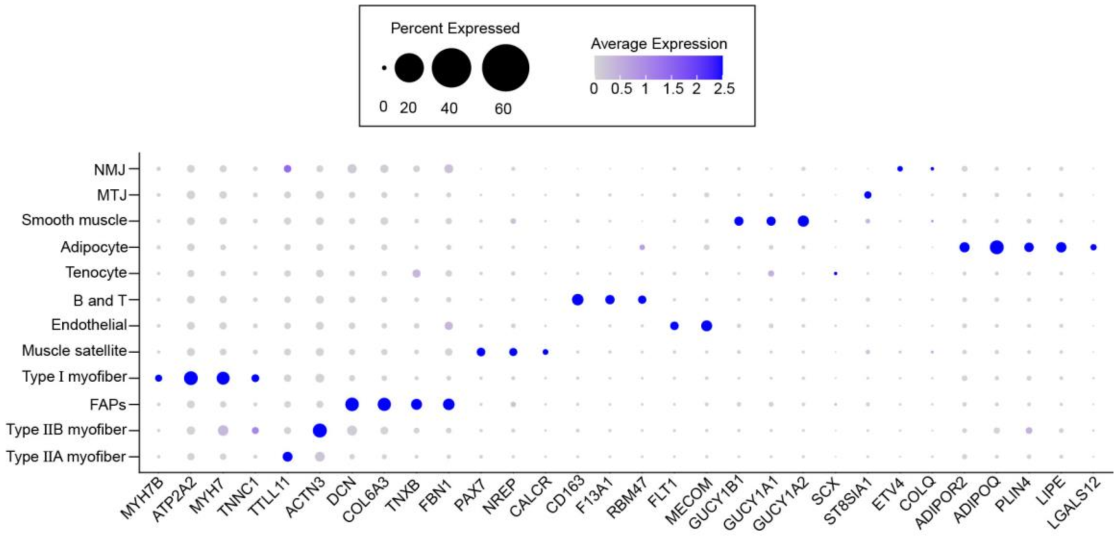

Figure S14. Classification of different cell types. Dot plot showing the average expression of specific marker genes in different cell types.

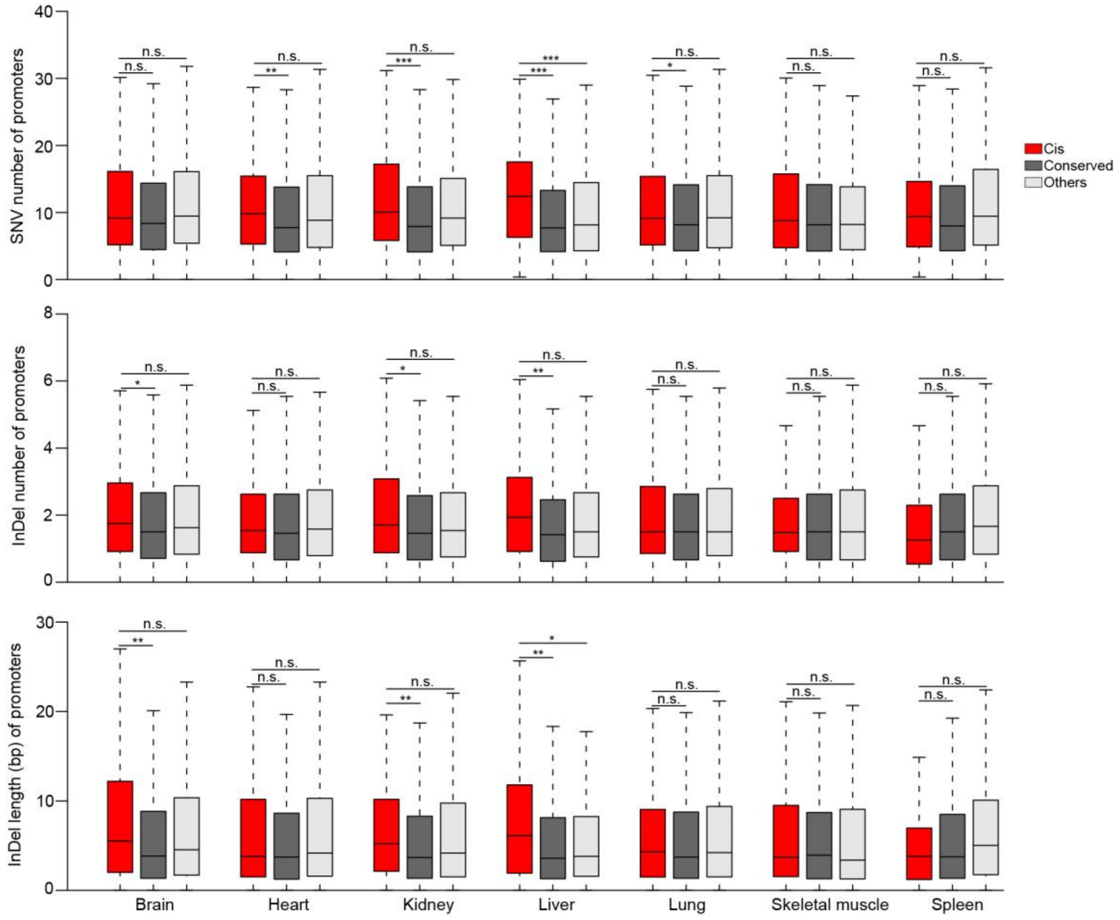

Figure S15. Distribution of SNV number (top), InDel number (middle) and Indel length (bp, bottom) of promoters in the combined of lncRNAs and TUCPs. The promoters were defined as upstream 2kb regions of TSS sites. The *trans*-regulated effects in lncRNAs and TUCPs were classified as others due to the limited number. Median of 9.71 SNVs in *cis* vs. 8.04 in conserved and 9.01 in others, $P < 0.001$ for liver; median of 1.58 InDels in *cis* vs. 1.46 in conserved and 1.54 in others, not significant; median of 4.54 bp InDles in *cis* vs. 3.75 in conserved and 4.21 in others, not significant.

The *P*-values were calculated by Wilcoxon rank-sum test (n.s. means: $P \geq 0.05$; * means: $0.01 < P < 0.05$; ** means: $0.001 < P < 0.01$; *** means: $P < 0.001$).

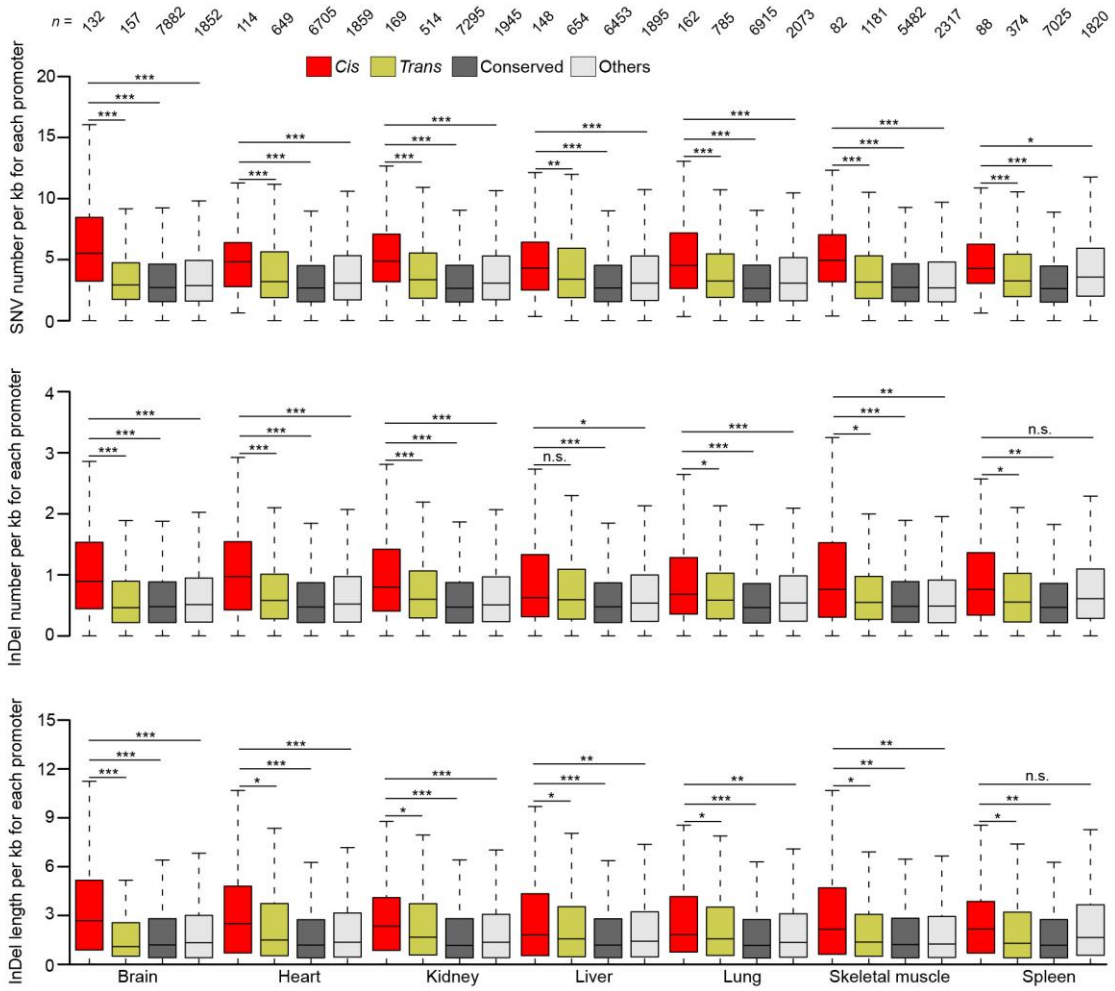

Figure S16. Distribution of the density for SNV number (top), InDel number (middle) and Indel length (bp, bottom) of the peak-annotated promoters in PCGs. The *P*-values were calculated by Wilcoxon rank-sum test (n.s. means: $P \geq 0.05$; * means: $0.01 < P < 0.05$; ** means: $0.001 < P < 0.01$; *** means: $P < 0.001$).

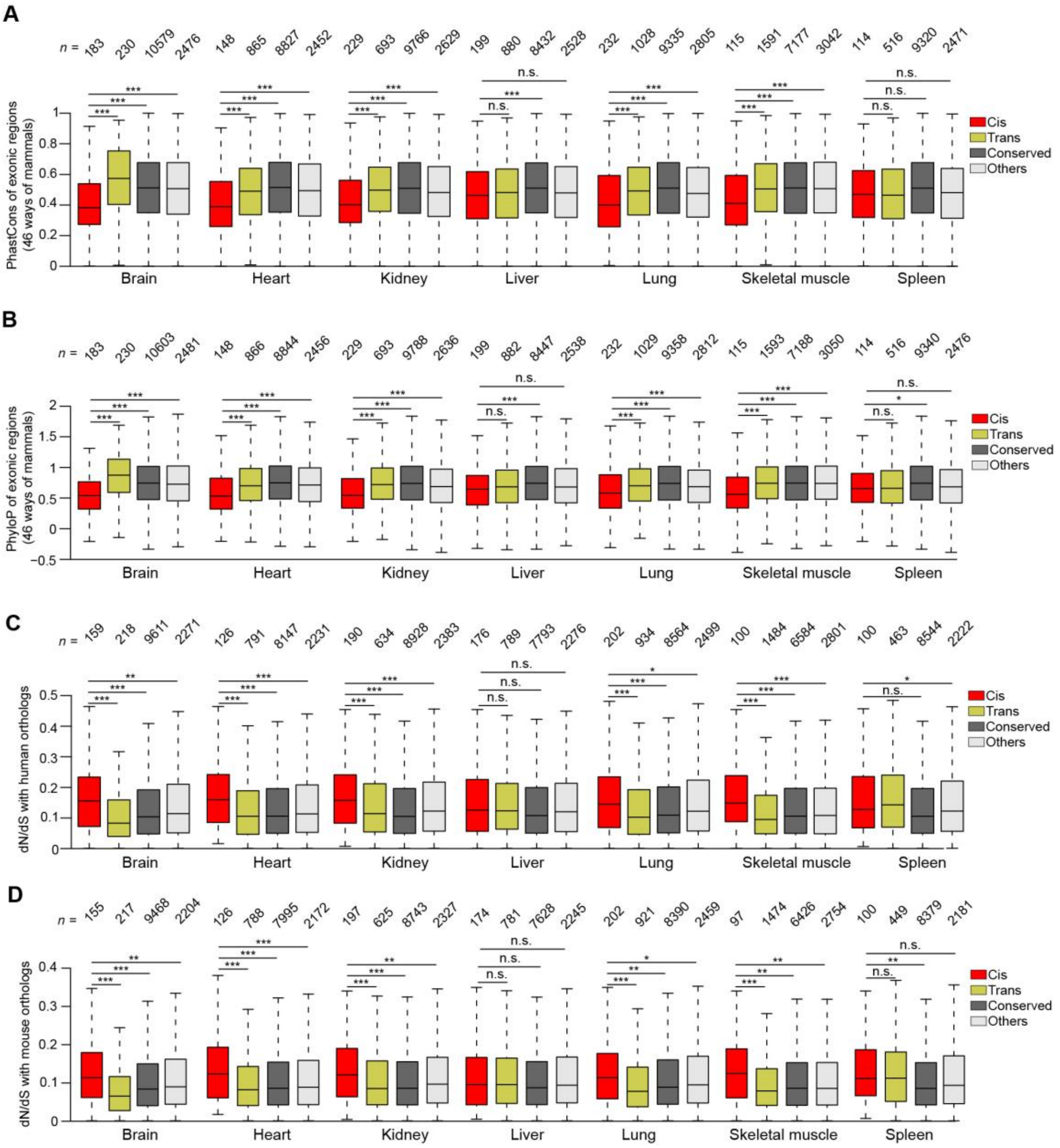

Figure S17. The conservation comparison between different regulatory categories for PCGs. (**A**) PhastCons values of PCG exonic regions were used to assess the conservation level between different regulatory categories (median of 0.42 in *cis* vs. 0.5 in *trans*, 0.51 in conserved and 0.49 in others, $P < 0.001$ except for liver and spleen). (**B**) PhyloP values of PCG exonic regions were used to assess the conservation level between different regulatory categories (median of 0.57 in *cis* vs. 0.72 in *trans*, 0.74 in conserved and 0.7 in others, $P < 0.001$ except for liver and spleen). (**C**) The dN/dS ratio of PCGs between pig and human for different regulatory categories (median of 0.14 in *cis* vs. 0.11 in *trans*, 0.11 in conserved and 0.12 in others, $P < 0.05$ except for

liver and spleen). (**D**) The dN/dS ratio of PCGs between pig and mouse for different regulatory categories (median of 0.11 in cis vs. 0.08 in trans, 0.09 in conserved and 0.09 in others, $P < 0.05$ except for liver and spleen).

The *P*-values were calculated by Wilcoxon rank-sum test (n.s. means: $P \geq 0.05$; * means: $0.01 < P < 0.05$; ** means: $0.001 < P < 0.01$; *** means: $P < 0.001$).
