# Supplementary material for: Allele-specific regulatory effects on the pig transcriptome
Source: Gigascience. 2023 Sep 30;12:giad076. doi: 10.1093/gigascience/giad076 (PMC10541795; doi:10.1093/gigascience/giad076)
Supplement: giad076_Response_to_Reviewer_Comments_Original_Submission [file giad076_response_to_reviewer_comments_original_submission.pdf]

Author's Response To Reviewer Comments

Dear Dr. Zhang,
We are thrilled to receive the positive responses from you. We would like to express our sincere gratitude for considering our manuscript for publication in GigaScience.
We deeply appreciate the insightful and constructive comments provided by the two reviewers, as well as your invaluable assistance in enhancing the manuscript. We have thoroughly reviewed both your comments and those of the reviewers, and we are confident that we have adequately addressed all the questions and concerns raised. Please find our detailed responses in the attaching file ‘Response_letter.pdf’, addressing them point by point. We hope that these responses meet your expectations.
