# Supplementary material for: Allele-specific regulatory effects on the pig transcriptome
Source: Gigascience. 2023 Sep 30;12:giad076. doi: 10.1093/gigascience/giad076 (PMC10541795; doi:10.1093/gigascience/giad076)
Supplement: giad076_Reviewer_1_Report_Original_Submission — Xuewen Xu -- 4/8/2023 Reviewed [file giad076_reviewer_1_report_original_submission.pdf]

Reviewer Report

Title: Allele-specific regulatory effects on the pig transcriptome

Version: Original Submission **Date:** 4/8/2023

Reviewer name: Xuewen Xu

Reviewer Comments to Author:

The authors analyzed allele specific expression using a reciprocal cross model, and distinguish the ASE genes into imprinting and sequence-dependent types. The result is interesting and valuable, but the following questions should be considered.1.L43 "promotes" should be "promoters".2.L82-L92, It is not necessary to repeat the introduction of materials and main results in this part.3.L150-151 It is not clear how to build N-masking genomes using phased SNVs. Please add references and describe the detail method building the N-masking genome. And ASE is sensitive to mapping bias, could you please provide the evaluation result of the mapping bias?4.L166-L183, Although the authors providing a reference to support the classification of expression regulatory categories between breeds, it is still unclear why it could be classified seven categories, and each category represents what kind of character and biological significance. Generally for gene expression, cis-regulation indicate a variant controlling the gene expression of the same chromosome, trans-regulation indicate a variant controlling the gene expression of another chromosome, including two aspects, the target gene on another homologous chromosome or on non-homologous chromosome of the variant. But in the present study, the classification is different from the traditional classification. Confusingly, in the section of discussion, the authors discussed the trans-regulatory variants and cis-regulatory variants, it seems to be back to the traditional classification. So the classification and its biological significance should be explained in detail and discussed further.5.L217 Add version information of Metascape online tool.6.L297, 'the majority showed evidence', the majority of what?7.L382-383 In FAANG (Functional Annotation of Animal Genomes), the promoter-related regions of different tissues have been identified based on different histone peaks in pigs (Pan et al. 2021). The use of annotated promoter-related regions is more representative rather than a hard condition (2kb upstream regions of TSS).8.L450-451, "higher proportion of trans-regulatory effects" is not related to artificial selection, the conclusion is questionable. Please revise it.9.L539 changes "Ï‡-test" to "Ï‡2-test".

Level of Interest

Please indicate how interesting you found the manuscript: Choose an item.

Quality of Written English

Please indicate the quality of language in the manuscript: Choose an item.

Declaration of Competing Interests

Please complete a declaration of competing interests, considering the following questions:

- Have you in the past five years received reimbursements, fees, funding, or salary from an organisation that may in any way gain or lose financially from the publication of this manuscript, either now or in the future?
- Do you hold any stocks or shares in an organisation that may in any way gain or lose financially from the publication of this manuscript, either now or in the future?
- Do you hold or are you currently applying for any patents relating to the content of the manuscript?
- Have you received reimbursements, fees, funding, or salary from an organization that holds or has applied for patents relating to the content of the manuscript?
- Do you have any other financial competing interests?
- Do you have any non-financial competing interests in relation to this paper?

If you can answer no to all of the above, write 'I declare that I have no competing interests' below. If your reply is yes to any, please give details below.

I declare that I have no competing interests

I agree to the open peer review policy of the journal. I understand that my name will be included on my report to the authors and, if the manuscript is accepted for publication, my named report including any attachments I upload will be posted on the website along with the authors' responses. I agree for my report to be made available under an Open Access Creative Commons CC-BY license (http://creativecommons.org/licenses/by/4.0/). I understand that any comments which I do not wish to be included in my named report can be included as confidential comments to the editors, which will not be published.

Choose an item.

To further support our reviewers, we have joined with Publons, where you can gain additional credit to further highlight your hard work (see: https://publons.com/journal/530/gigascience). On publication of this paper, your review will be automatically added to Publons, you can then choose whether or not to claim your Publons credit. I understand this statement.

Yes Choose an item.
