# Supplementary material for: Allele-specific regulatory effects on the pig transcriptome
Source: Gigascience. 2023 Sep 30;12:giad076. doi: 10.1093/gigascience/giad076 (PMC10541795; doi:10.1093/gigascience/giad076)
Supplement: giad076_Reviewer_2_Report_Original_Submission — Martien Groenen -- 4/11/2023 Reviewed [file giad076_reviewer_2_report_original_submission.pdf]

Reviewer Report

Title: Allele-specific regulatory effects on the pig transcriptome

Version: Original Submission **Date:** 4/11/2023

Reviewer name: Martien Groenen

Reviewer Comments to Author:

The manuscript presents a well-designed study based on reciprocal crosses between two divergent pig breeds. The authors show that a cross between European and Chinese breeds, which represent ~1 million years of divergence, is a powerful approach to study ASE and imprinting in this species. Although the results are presented clearly, there is an imbalance in the structure and much of the results section is comprised of information better suited in the Material and Methods or in the Discussion sections. E.g., Lines 224-233 are partly repetitive and most of this information better fits in the Material and Methods section while lines 310-342 are already more Discussion rather than Results.In line 242 it is stated that a total coverage of ~126-fold was obtained for the whole genome sequencing. I think it is more informative to indicate the average sequence coverage obtained for each of individuals sequenced. I was also very much confused by supplementary figure S2, that suggests that each of the individuals was sequenced at ~120-fold coverage, something that seems highly unlikely. If 125x indeed refers to the total coverage, then the average coverage per individual is only 3x, which seems rather low to be able to unequivocally identify heterozygous sites.Figure 2G: Please explain the expression pattern shown for INPP5F. For the brain RNAseq coverage is seen for all exons, while in brain this is only seen for the last exon.I find the reasoning in lines 336-342 rather strange. Wouldn't one expect a worse innate immunity in these breeds that are kept under high environmental xenobiotic pressure. Furthermore, this section tends to be rather "storytelling". Related to that the GO enrichment analyses. The results for these are highly biased towards genes that are expressed in a given tissue, and always will point towards functions of importance to that tissue done". Instead of using all PCG's in the porcine genome as background only the genes that are expressed in a specific tissue need to be used (so excluding the PCG's not expressed).It is not mentioned how many individuals were used for each of the two breeds for the single nuclei experiments.Lines 384-388: These results are biased. In regions that overall show a higher sequence variation, the chances of being able to distinguish the two alternative transcripts from the two alleles is higher. Therefore, the conclusion in line 393-394 that this shows that cis-regulated genes are less conserved is jumping to conclusions without solid support.The authors tend to make too strong statements based on their results. E.g. lines 442-443: I do not agree with this statement. The results provide a better description of genomic imprinting, but they do not greatly expand the current understanding of genomic imprinting. Likewise, the statement made in lines 452-459 is not supported by the results.Minor commentsLine 38: Insert "are" between "that" and "regulated"Line 43: "promotors" instead of "promotes"Line 82: Insert "the" between "on" and "pig"Line 239-240: Replace "we sequenced whole genome sequencing data" by "we obtained whole genome sequencing data"Throughout the paper I suggest o use "trans-regulated" instead od "trans-regulatory"Line 312: "pressure" instead of "pressions"Line 344: Delete "in the testable tissues"Line 386: Replace "to any other" by "to the other"

and "category" by "categories"Line 398: I suggest to replace "Interestingly" by "As expected"Lines 405-406; This is a rather obvious statement and shown numerous times in published eQTL studies.Line 408: Please rephrase "a tissue-dependent manner of regulatory effects on gene expression"Line 429-432: I would make this statement more general: In general, this high heterozygous ratio demonstrates European and Asian pigs, like Berkshire and Tibetan, are ideal to build reciprocal cross models to study allele-specific expression differences between breeds.

Level of Interest

Please indicate how interesting you found the manuscript: Choose an item.

Quality of Written English

Please indicate the quality of language in the manuscript: Choose an item.

Declaration of Competing Interests

Please complete a declaration of competing interests, considering the following questions:

- Have you in the past five years received reimbursements, fees, funding, or salary from an organisation that may in any way gain or lose financially from the publication of this manuscript, either now or in the future?
- Do you hold any stocks or shares in an organisation that may in any way gain or lose financially from the publication of this manuscript, either now or in the future?
- Do you hold or are you currently applying for any patents relating to the content of the manuscript?
- Have you received reimbursements, fees, funding, or salary from an organization that holds or has applied for patents relating to the content of the manuscript?
- Do you have any other financial competing interests?
- Do you have any non-financial competing interests in relation to this paper?

If you can answer no to all of the above, write 'I declare that I have no competing interests' below. If your reply is yes to any, please give details below.

I declare that I have no competing interests

I agree to the open peer review policy of the journal. I understand that my name will be included on my report to the authors and, if the manuscript is accepted for publication, my named report including any attachments I upload will be posted on the website along with the authors' responses. I agree for my report to be made available under an Open Access Creative Commons CC-BY license (http://creativecommons.org/licenses/by/4.0/). I understand that any comments which I do not wish to

be included in my named report can be included as confidential comments to the editors, which will not be published.

Choose an item.

To further support our reviewers, we have joined with Publons, where you can gain additional credit to further highlight your hard work (see: https://publons.com/journal/530/gigascience). On publication of this paper, your review will be automatically added to Publons, you can then choose whether or not to claim your Publons credit. I understand this statement.

Yes Choose an item.
